# Synthesis of core@shell catalysts guided by Tammann temperature

**DOI:** 10.1038/s41467-024-44705-5

**Published:** 2024-01-10

**Authors:** Pei Xiong, Zhihang Xu, Tai-Sing Wu, Tong Yang, Qiong Lei, Jiangtong Li, Guangchao Li, Ming Yang, Yun-Liang Soo, Robert David Bennett, Shu Ping Lau, Shik Chi Edman Tsang, Ye Zhu, Molly Meng-Jung Li

**Affiliations:** 1 Department of Applied Physics, The Hong Kong Polytechnic University, Hong Kong, China; 2 National Synchrotron Radiation Research Center, Hsinchu, 30076 Taiwan; 3 Department of Physics, National Tsing Hua University, Hsinchu, 30013 Taiwan; 4 CSIRO Energy, Clayton Laboratories, Clayton South, VIC 3168 Australia; 5 Wolfson Catalysis Centre, Department of Chemistry, University of Oxford, Oxford, OX1 3QR UK

**Keywords:** Materials for energy and catalysis, Nanoscale materials, Materials chemistry

## Abstract

Designing high-performance thermal catalysts with stable catalytic sites is an important challenge. Conventional wisdom holds that strong metal-support interactions can benefit the catalyst performance, but there is a knowledge gap in generalizing this effect across different metals. Here, we have successfully developed a generalizable strong metal-support interaction strategy guided by Tammann temperatures of materials, enabling functional oxide encapsulation of transition metal nanocatalysts. As an illustrative example, Co@BaAl_2_O_4_ core@shell is synthesized and tracked in real-time through in-situ microscopy and spectroscopy, revealing an unconventional strong metal-support interaction encapsulation mechanism. Notably, Co@BaAl_2_O_4_ exhibits exceptional activity relative to previously reported core@shell catalysts, displaying excellent long-term stability during high-temperature chemical reactions and overcoming the durability and reusability limitations of conventional supported catalysts. This pioneering design and widely applicable approach has been validated to guide the encapsulation of various transition metal nanoparticles for environmental tolerance functionalities, offering great potential to advance energy, catalysis, and environmental fields.

## Abstract (web summary)

The authors report a synthetic strategy to create core@shell catalysts using strong metal-support interactions and low-Tammann-temperature compounds. The resulting materials are highly stable and may be useful in industrial applications.

## Introduction

Transition metal nanoparticles (M_T_ NPs) exhibit excellent activities for many important catalytic reactions, such as methane (CH_4_) or ammonia (NH_3_) conversions^1–5^, yet stability drawbacks hinder their practical applications. This is because the highly active surface atoms are generally thermodynamically unstable, leading to severe NPs sintering via secondary nucleation, recrystallization, ripening, particle migration, and coalescence^6–8^. To preserve the NPs’ stability, different approaches have been developed. Among them, the core@shell architecture offers an advantageous structure by isolating the active nanoparticle cores with outer shells to prevent sintering during high-temperature catalytic reactions^9,10^. The most commonly used core@shell synthesis method is to physically confine the NPs in porous materials (e.g., silica, alumina, zeolites, and so forth.)^11,12^. Despite various choices and vast sources of confining materials, the limitation of active site blockage by the inert supports is often observed, thereby diminishing the catalytic performance^13^.

Alternatively, strong metal-support interactions (SMSI) have been engineered to produce core@shell structures for stabilizing and modifying active metal NPs. For example, on the important industrial Cu/ZnO/Al_2_O_3_ methanol synthesis catalyst, it is found that the partially reduced zinc oxides can interact with metallic Cu NPs via the SMSI effect^14^, forming a protective layer to prevent the sintering of Cu NPs. Due to the unique geometric and sintering-resistant interfacial interactions, various SMSI metal and support compositions have been explored and studied^15^. It is observed that at the metal-support interfaces, the reducible metal oxide substrates (e.g., TiO_2_, V_2_O_3_, CeO_2_, and Ta_2_O_5_) can be thermally activated and migrated onto metal NPs surface (typically platinum group metals like Pt, Pd, and so on) under hydrogen (H_2_) atmosphere at high temperature, forming complete encapsulation of the NPs by the oxides^15–17^. These SMSI metal@support configurations not only dramatically improve the thermodynamic stability of the NPs against sintering but also impart tunable versatility of the catalytic properties via modulating the core@shell interface^18–20^.

Unfortunately, the high temperature required in the thermal activation step of SMSI restricts the application of this strategy to NPs with low melting points. For those with inherently low melting temperatures, such as Fe, Co, Ni, Cu, Au, and so on, the NPs tend to sinter before the encapsulation occurs^21^. Hence, to achieve successful encapsulation of M_T_ NPs through SMSI, it is critical to activate the supporting metal oxides with sufficient mobility before the M_T_ NPs sinter. Several successes have been reported, such as HCO_x_-adsorbate-mediated SMSI encapsulation on TiO_2_- and Nb_2_O_5_-supported Rh NPs^22^, CO_2_-enhanced redox-inert MgO encapsulation on Au NPs^23^, and the adsorbate-induced SMSI reconstruction of the commercial Cu/ZnO/Al_2_O_3_ that maximizes the activity and stability^24^. However, these methods are highly material-specific and thus hard to be extended to other systems. Therefore, novel generalizable core@shell synthesis strategies are highly demanded.

In this work, we establish a general and straightforward approach for achieving thermal-induced SMSI encapsulation on M_T_ NPs. The approach involves utilizing the empirical Tammann temperature (T_Tam_) phenomenon, which describes the atoms in the solid-state materials becoming loosened with higher mobility and reactivity at temperatures higher than c.a. 1/2 of the melting point^25^. We leverage the advantage of the low T_Tam_ compounds that can be thermally activated and reacted at relatively low temperatures. Once a coating of low T_Tam_ compounds is formed on M_T_ NPs, further overlayer functionalization can be realized through a solid-state reaction, resulting in the formation of the highly thermally stable protective layer of the catalyst support.

## Results and Discussion

### Synthesis rationale for thermal encapsulation based on T_Tam_

**Fig. 1:**
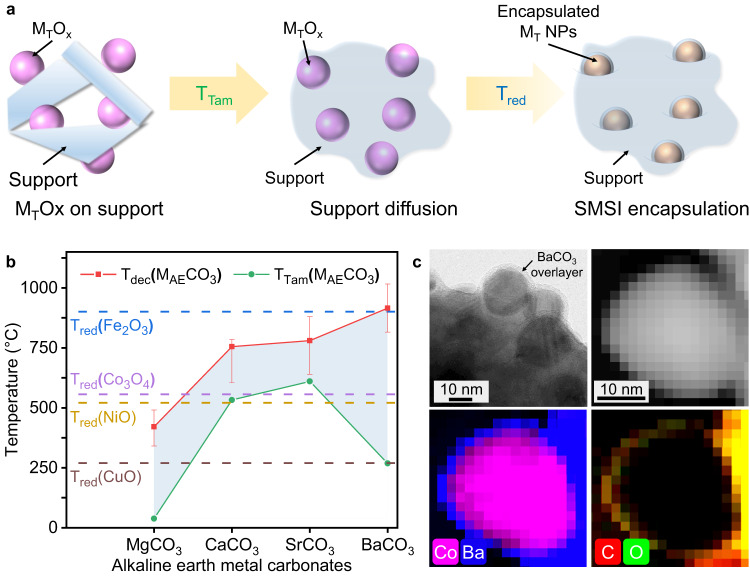
Synthesis rationale for thermal encapsulation based on Tammann temperature (T_Tam_). **a** Schematic illustration of the designed synthesis rationale for this thermal-induced encapsulation strategy guided by Tammann temperature; M_T_O_x_ indicates transition metal nanoparticles oxides, and M_T_ NPs indicates transition metal nanoparticles. **b** Schematic of the estimated suitable temperature range (or encapsulable window, shadowed in blue) to enable encapsulation on in-situ formed M_T_ NPs with alkaline earth metal carbonates (M_AE_CO_3_) through thermal-induced strong metal-support interaction (SMSI); T_Tam_(M_AE_CO_3_) indicates Tammann temperature of M_AE_CO_3_, T_dec_(M_AE_CO_3_) indicates the decomposition temperature of M_AE_CO_3_, and T_red_ indicates the reduction temperature of M_T_O_x_; the upper and lower limits of the error bars for the decomposition temperature are taken respectively from the starting and ending temperatures of the decomposition peak of the substance. **c** Transmission electron microscopy (TEM), scanning transmission electron microscopy (STEM) images and electron energy loss spectroscopy (EELS) element distribution maps of Co@BaCO_3_ (additional Co@BaCO_3_ EELS elemental mapping analysis can be found in Supplementary Fig. 8). Source data are provided as a Source Data file.

Herein, we synthesize M_T_ NPs by in-situ reduction from the corresponding M_T_ oxides (M_T_O_x_) to avoid the troublesome multi-step processes and handling of active M_T_ NPs materials. To establish SMSI encapsulation, we refer to the T_Tam_ as an indicator to select suitable encapsulating materials, i.e., supports, for the targeted metal catalysts. More specifically, as shown in Fig. 1a, the T_Tam_ of the encapsulating materials, denoted as T_Tam_(support), should be lower than the reduction temperature (T_red_) of M_T_O_x_, denoted as T_red_(M_T_O_x_). According to this rule, the support can facilitate more efficient diffusion, acting as a mobile media to establish contact with the M_T_O_x_ at T_Tam_(support). During the formation of M_T_ NP at T_red_(M_T_O_x_), encapsulation can take place due to the tendency to reduce the surface energy of M_T_ NPs. This process helps prevent the excessive growth of M_T_ particles. Meanwhile, the decomposition temperature (T_dec_) of the encapsulating materials, denoted as T_dec_(support), must be higher than the T_red_(M_T_O_x_) to avoid the loss of high mobility due to the phase/structure change. Based on these considerations, if one support material can fit in the simple rule of T_Tam_(support) <T_red_(M_T_O_x_) <T_dec_(support), then it is expected to exhibit high mobility to achieve SMSI encapsulation on M_T_ NPs at T_red_(M_T_O_x_).

As the proof-of-concept demonstration, we choose low T_Tam_ alkaline earth metal (common catalyst promoters) in the carbonate form (M_AE_CO_3_) as the candidate support. The phase-changing temperatures of the common non-noble M_T_O_x_ (M_T_ = Fe, Co, Ni, Cu) and M_AE_CO_3_ (M_AE_ = Mg, Ca, Sr, Ba) combinations are mapped in Fig. 1b. The T_dec_(M_AE_CO_3_) and T_red_(M_T_O_x_) are measured by H_2_ temperature-programmed decomposition and reduction experiments, respectively (see details in Supplementary Figs. 6 and 7, Supplementary Table 1), while the T_Tam_(M_AE_CO_3_) are retrieved from literature^21,26–28^. In Fig. 1b, the region sandwiched by T_dec_(M_AE_CO_3_) and T_Tam_(M_AE_CO_3_) (shadowed in blue) indicates the suitable temperature window (or encapsulable window) to theoretically allow for the facile SMSI encapsulation on the M_T_ NPs if the corresponding T_red_(M_T_O_x_) falls into this range. A test synthesis on the Co and BaCO_3_ combination has been attempted to prove our concept. We obtain uniformly distributed Co NPs encapsulated in BaCO_3_ (i.e., Co@BaCO_3_) through one-step thermal treatment at the temperature around T_red_(Co_3_O_4_) in H_2_ atmosphere (see details in Fig. 1c, Supplementary Fig. 8 and Supplementary Table 3). This result demonstrates the viability of our proposed strategy for the convenient thermal encapsulation of M_T_ NPs guided by Tammann temperatures.

### One-step metal encapsulation and overlayer functionalization

**Fig. 2:**
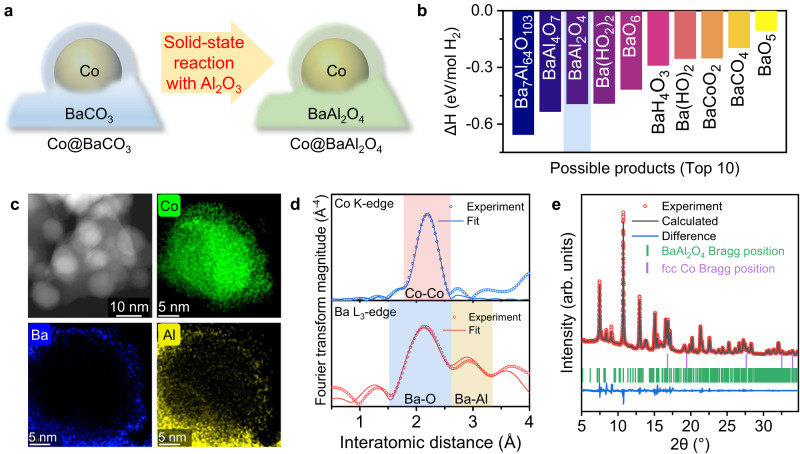
One-step metal encapsulation and overlayer functionalization. **a** Schematic illustration of the subsequent overlayer functionalization step following the thermal-induced strong metal-support interaction (SMSI) encapsulation. **b** Rapid computational screening of reaction enthalpy changes (ΔH) for forming possible products from solid-state reactions between BaCO_3_, Al_2_O_3_ and Co_3_O_4_ in H_2_. ΔH were obtained from the Materials Project database. The lower the ΔH, the more stable the reaction product. **c** Scanning transmission electron microscopy (STEM) image and energy dispersive X-ray spectroscopy (EDS) element distribution maps of Co@BaAl_2_O_4_ (More details in Supplementary Fig. 11d). **d** Extended X-ray absorption fine structure (EXAFS) fitting curves in R-space for the Co K-edge and Ba L_3_-edge of Co@BaAl_2_O_4_. **e** Rietveld refinement of the synchrotron X-ray diffraction (SXRD) pattern (λ = 0.5904 Å) of Co@BaAl_2_O_4_. Source data are provided as a Source Data file.

To take full advantage of the high reactivity of the low-T_Tam_ M_AE_CO_3_, we propose further functionalizing the BaCO_3_ overlayer during the Co NP formation. Considering alkaline earth metal aluminates (i.e., (M_AE_)_x_Al_y_O_z_) are classic catalyst support materials with high thermal resistance, good hydrothermal stability, and prominent interactions with the active species^29,30^, we attempt to introduce Al_2_O_3_ into the encapsulation system to engineer the overlayer functionalization (Fig. 2a). By employing rapid computational sorting of the formation energies of different aluminate products from the solid-state reaction between BaCO_3_ and Al_2_O_3_ (Fig. 2b and Supplementary Fig. 9), we identify that the most suitable overlayer aluminate composition is BaAl_2_O_4_, and reject the more stable BaAl_4_O_7_ and Ba_7_Al_64_O_103_ because they require harsh synthesis conditions (>1100 °C)^31,32^.

To realize Co NP formation, BaCO_3_ encapsulation, and BaAl_2_O_4_ formation in a single thermal treatment, we start with mixing the Co, Ba and Al metal precursors with a composition of 40Co:20Ba:40Al through an automatically pH-controlled co-precipitation method, followed by temperature- and flow-controlled air-calcination (Supplementary Fig. 1). After the thermal treatment in H_2_, the formation of Co NPs and BaAl_2_O_4_ can be confirmed by the X-ray diffraction (XRD) patterns (Supplementary Fig. 10a). Scanning transmission electron microscopy (STEM) and energy dispersive X-ray spectroscopy (EDS) mapping (Fig. 2c and Supplementary Fig. 11d) clearly show the encapsulation structure with the well-rounded BaAl_2_O_4_ overlayer in close contact with the Co NPs. The encapsulated Co NPs show relatively uniform size distributions in the range of 10~15 nm, in line with the crystal size derived from XRD (Supplementary Fig. 10b and Supplementary Table 4). Transmission electron microscopy (TEM) images and corresponding fast Fourier transform (FFT) patterns of Co@BaAl_2_O_4_ reveal the fcc Co and BaAl_2_O_4_ lattice fringes (Supplementary Fig. 11), which is fully consistent with the Co K-edge and Ba L_3_-edge extended X-ray absorption fine structure (EXAFS) (Fig. 2d, Supplementary Figs. 12 and 13, and Supplementary Tables 5 to 8) and the synchrotron X-ray diffraction (SXRD) results (Fig. 2e).

Interestingly, noticeable oxygen vacancies in the BaAl_2_O_4_ overlayer of Co@BaAl_2_O_4_ can be detected by X-ray photoelectron spectroscopy (XPS) and electron paramagnetic resonance (EPR) spectroscopy (Supplementary Fig. 14a, b). In addition, Ar adsorption-desorption analyses reveal the porous structure of Co@BaAl_2_O_4_ (Supplementary Fig. 14c, d), presumably due to the presence of oxygen vacancies when synthesizing BaAl_2_O_4_ under H_2_ environment. This porous feature is particularly important for catalysis as they impart the good permeability of core@shell structure for the reactant molecules to access the active metal core while maintaining high stability from encapsulation.

It is important to note that the observed Co@BaAl_2_O_4_ encapsulation cannot be realized by direct synthesis using BaAl_2_O_4_ (see details in Supplementary Fig. 15 and Supplementary Table 9), reflecting the necessity of employing low T_Tam_ BaCO_3_ for our proposed SMSI encapsulation process. In addition, severe Fe or Cu NP coalescence is observed in the control experiments involving M_T_O_x_-M_AE_CO_3_ combinations where the T_red_(M_T_O_x_) falls either outside or on the edge of the encapsulable window, further validating our proposed thermal encapsulation and sintering prevention strategy guided by Tammann temperature (Supplementary Figs. 16 and 17).

### Real-time observation for the encapsulation mechanism

**Fig. 3:**
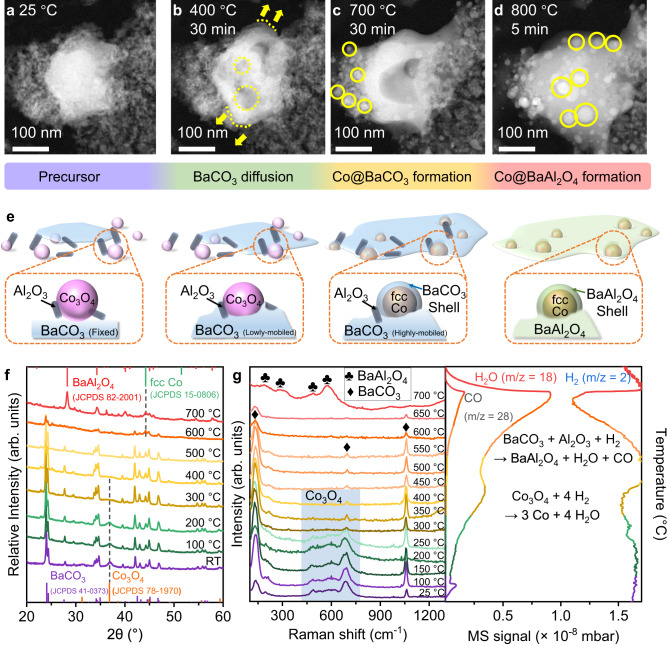
Real-time observation for the encapsulation mechanism. In-situ scanning transmission electron microscopy (STEM) images at the initial state at room temperature (**a**), BaCO_3_ diffusion stage when the temperature is above T_Tam_(BaCO_3_) (**b**) (the yellow dotted lines represent the expansion of the BaCO_3_ boundary, and the dashed yellow circles show the vacancies inside the BaCO_3_), Co nanoparticle (NP) formation stage when the temperature is above the reduction temperature (T_red_) of Co_3_O_4_ (**c**) (the yellow circles indicate the Co nucleation and growth), and Co@BaAl_2_O_4_ formation stage after the solid-state reaction between BaCO_3_ and Al_2_O_3_ (**d**). **e** Schematic illustration of the mechanism for this thermal-induced encapsulation strategy guided by Tammann temperature. **f** X-ray diffraction (XRD) patterns of the 40Co:20Ba:40Al reduced at different temperatures ranging from room temperature to 700 °C. **g** In-situ Raman spectra of the 40Co:20Ba:40Al reduced at different temperatures ranging from room temperature to 700 °C, and mass spectrometry (MS) signals of the outlet gas monitored during the H_2_ reduction process (tracking m/z = 2 for H_2_, m/z = 18 for H_2_O and m/z = 28 for CO). Source data are provided as a Source Data file.

The Co@BaAl_2_O_4_ synthesis process is further investigated to explore the encapsulation formation pathway and mechanism. Starting with the 40Co:20Ba:40Al precursor, the elemental composition analyses indicate a similar atomic ratio of Co, Ba, and Al at c.a. 2:1:2 at different stages of the synthesis process (i.e., as-precipitated, as-calcined, and thermally treated in H_2_, Supplementary Fig. 19 and Supplementary Table 11). Yet distinct morphologies are revealed by TEM, where the encapsulation is only formed after the thermal treatment in H_2_ (Supplementary Fig. 20). Direct real-time visualization of the encapsulation process during the H_2_ thermal treatment is then conducted using in-situ atmospheric STEM equipped with an environmental cell (see Supplementary Fig. 4 for experiment setup and details). At 25 °C, the calcined precursor is initially a mixture of plate-like Co_3_O_4_ and rod-like Al_2_O_3_ NPs in contact with much larger bulk of BaCO_3_ (Supplementary Fig. 21). STEM image indicates a clear boundary between the dark Co_3_O_4_-Al_2_O_3_ mixture and the bright BaCO_3_ bulk particle (Fig. 3a). When the temperature is ramped to above 400 °C (Fig. 3b and Supplementary Movie 1), the boundary of the BaCO_3_ particle blurs and some darker pores (marked by dashed circles) appear in the interior, which can be attributed to the increased lattice mobility and subsequent outward diffusion as evidenced by the boundary propagation (the dotted lines). Raising the temperature to 500 °C leads to more obvious diffusion of BaCO_3_ with dark pores growing inside, agreeing well with the expanding Ba element distribution observed from EDS elemental mapping (Supplementary Fig. 22a, b). In contrast, no obvious morphology change is observed in the Co/Al-containing area until the bright spheres emerge at 600 °C and become more identifiable at 700 °C (Fig. 3c and Supplementary Movie 2), indicating the in-situ formation of Co NPs from reducing Co_3_O_4_. The Co NPs are surrounded by highly mobile or liquified BaCO_3_, forming the initial encapsulation. When the cell is heated from 700 to 800 °C, a dynamic reaction can be seen with BaCO_3_ decomposition at the expense of the Al_2_O_3_ rods in proximity, implying the solid-state formation of the functional BaAl_2_O_4_ overlayer (Fig. 3d, Supplementary Fig. 22c and Supplementary Movie 3), as confirmed by selected area electron diffraction (SAED) (Supplementary Fig. 22d). The observed morphology evolution is summarized in Fig. 3e, in full accordance with our proposed SMSI-induced encapsulation strategy guided by Tammann temperature.

XRD and in-situ Raman spectroscopy equipped with online MS (see details in Supplementary Fig. 3) are also employed to supplement the real-time phase evolution and the solid-state reactions. At the initial stage, typical XRD patterns of BaCO_3_ and Co_3_O_4_ are recorded (Fig. 3f), with the absence of Al_2_O_3_ fingerprint peaks, presumably overwhelmed by the signals of Co_3_O_4_ or the nano crystallinity of gamma phase Al_2_O_3_ (Supplementary Fig. 21d). As the temperature of thermal treatment increases to 400 °C, the characteristic peaks of Co_3_O_4_ at 31.27° and 36.84° fade with the simultaneous appearance of two new peaks at 44.22° and 51.52°, which can be assigned to the fcc Co. At 700 °C, the characteristic diffraction signal of BaCO_3_ is replaced by that of BaAl_2_O_4_, indicating the completion of the solid-state reaction between BaCO_3_ and Al_2_O_3_. Similarly, in-situ Raman spectroscopy reveals the gradual disappearance of Co_3_O_4_ fingerprints around ~490, ~525, ~610, and ~690 cm^-1^ before 400 °C^33^, and the replacement of BaCO_3_ fingerprints^34^ around ~139, ~698, and ~1060 cm^–1^ by those of BaAl_2_O_4_ around ~193, ~271, ~491, ~581, and ~680 cm^-1^ at 700 °C (Fig. 3g)^35^. Online MS is used to monitor the outlet gas during the thermal treatment under H_2_ flow. It can be observed that Co_3_O_4_ is reduced during 300~500 °C, with H_2_ consumed and H_2_O released. As the temperature is further increased to 700 °C, both CO and H_2_O peaks rise rapidly while consuming H_2_, which is consistent with the solid-state reaction between BaCO_3_ and Al_2_O_3_. In other words, these results echo the observed phase change process in the in-situ STEM. Note that the discrepancies in some phase-changing temperatures recorded by different characterization techniques are due to the distinct in-situ conditions, which do not affect the trend of the identified phase evolution and encapsulation mechanism.

All the evidence collectively indicates that (i) low T_Tam_ BaCO_3_ exhibits sufficient mobility and can migrate onto the in-situ formed Co NPs, and (ii) BaCO_3_ could readily react with Al_2_O_3_ to form BaAl_2_O_4_ as the final encapsulation overlayer (i.e., BaCO_3_ + Al_2_O_3_ + H_2_ → BaAl_2_O_4_ + CO + H_2_O), thanks to the high reactivity of low-T_Tam_ BaCO_3_ in the H_2_ atmosphere at a higher temperature. As a result, the complete encapsulation is realized on the Co NP surface with BaAl_2_O_4_ by forming a well-defined core@shell configuration.

### Excellent catalytic performance of encapsulated nanocatalyst

**Fig. 4:**
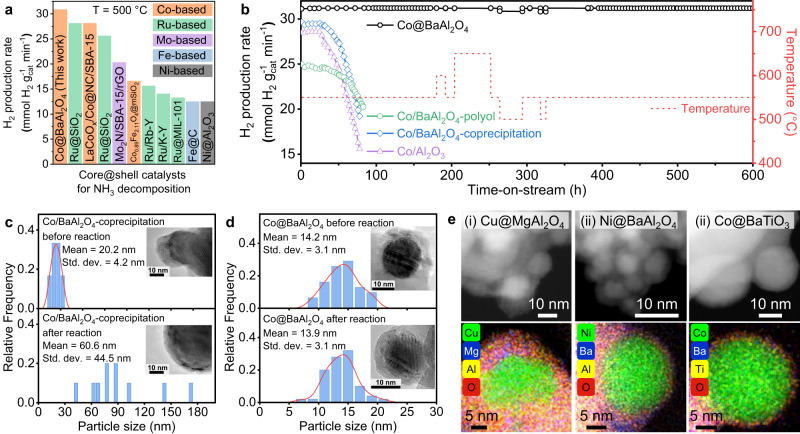
Excellent catalytic performance of encapsulated nanocatalyst. **a** Comparison of H_2_ production rate at 500 °C between the Co@BaAl_2_O_4_ and the selected core@shell catalysts used for NH_3_ decomposition reaction (details see Supplementary Table 11). **b** H_2_ production rate according to time-on-stream during the NH_3_ decomposition reaction over the core@shell Co@BaAl_2_O_4_ (grey blank balls), and the non-encapsulated catalysts including Co/Al_2_O_3_ (blue solid balls), and Co/BaAl_2_O_4_ samples prepared by co-precipitation (purple solid balls) and polyol method (green solid balls). The performance was evaluated at weight hourly space velocity (WHSV) of 30,000 mL g_cat_^–1^ h^–1^. **c** Co NP size distribution histograms of the non-encapsulated Co/BaAl_2_O_4_-coprecipitation before and after 80-hour NH_3_ decomposition reaction, inset shows the transmission electron microscopy (TEM) images of corresponding samples. **d** Co NP size distribution histograms of core@shell Co@BaAl_2_O_4_ before and after 80-hour NH_3_ decomposition reaction, inset shows the TEM images of corresponding samples. **e** scanning transmission electron microscopy (STEM) images and energy dispersive X-ray spectroscopy (EDS) element distribution maps of Cu@MgAl_2_O_4_, Ni@BaAl_2_O_4_, and Co@BaTiO_3_. Source data are provided as a Source Data file.

To assess the impact of BaAl_2_O_4_ encapsulation on the catalytic performance of the Co NPs at high temperatures, a series of Co nanocatalysts, both with and without encapsulation, are evaluated for their performance in thermal catalytic NH_3_ decomposition reaction. Typically, this reaction requires a temperature of approximately 650~800 °C for non-noble metal catalysts to achieve an NH_3_ conversion of over 80% without sacrificing the weight hourly space velocity (WHSV)^36,37^. Surprisingly, the Co@BaAl_2_O_4_ achieves almost complete conversion (~99%) at a much lower temperature of only 500 °C, at a high WHSV of 30,000 mL g_cat_^–1^ h^–1^ (Supplementary Fig. 25 and Supplementary Table 12), obtaining the H_2_ production rate of 30.9 mmol H_2_ g_cat_^–1^ min^–1^ and making it the best core@shell catalysts ever reported for NH_3_ decomposition (Fig. 4a). Compared to the state-of-the-art NH_3_ decomposition catalysts, the encapsulated Co@BaAl_2_O_4_ also represents one of the most effective catalysts in both non-noble and noble catalysts records (Supplementary Fig. 26 and Supplementary Table 13).

The most significant benefit of the core@shell catalyst configuration is demonstrated in long-term performance evaluation. After 50 hours of time-on-stream at 550 °C, the H_2_ production of the non-encapsulated catalysts, Co NPs supported on Al_2_O_3_ (Co/Al_2_O_3_) and on BaAl_2_O_4_ (synthesis details see Supplementary Fig. 15), significantly decreases, indicating poor long-term activity due to the severe sintering of Co particles in high-temperature reaction condition (Fig. 4b, c, Supplementary Figs. 27 and 28, and Supplementary Tables 14 and 15). In sharp contrast, the Co@BaAl_2_O_4_ nanocatalyst maintains a constantly high H_2_ production rate over 600 hours under identical testing condition (Fig. 4b). No significant change in morphology is observed for the post-reaction Co@BaAl_2_O_4_ (Fig. 4d, Supplementary Fig. 29, and Supplementary Table 16), indicating its superior thermochemical stability due to the advantageous core@shell structure. The EXAFS of the post-reaction Co@BaAl_2_O_4_ reveals the distinct Co and BaAl_2_O_4_ phases (Supplementary Fig. 29d, Supplementary Tables 17 and 18), suggesting no migration of Co species at the metal-support interfaces during the long-term testing. Remarkably, over a five-month-long experiment, the activity of Co@BaAl_2_O_4_ does not decrease despite intentional temperature fluctuations, system ONs/OFFs, and catalyst offload/reload events (Supplementary Fig. 30), demonstrating its excellent environmental tolerance and reusability for practical applications.

Furthermore, Co@BaAl_2_O_4_ demonstrates promising outcomes in the CH_4_ dry-reforming reaction, which typically requires even higher temperatures (>750 °C)^3^. As shown in Supplementary Fig. 31, Co@BaAl_2_O_4_ achieves the CH_4_ and CO_2_ conversion rates of 81.8% and 90.1%, respectively, nearing the thermodynamic equilibrium value and sustaining the performance for over 60 hours. In comparison to the benchmark catalysts reported in literature (Supplementary Table 19), the high efficiency and excellent stability identify Co@BaAl_2_O_4_ as a promising catalyst for the CH_4_ dry-reforming reaction. These results jointly manifest that our unconventional encapsulation strategy not only effectively stabilizes the nanocatalyst against the common sintering issue but also provides further opportunities to modify the performance for good catalytic applicability. Currently, the origin of the exceptional performance of Co@BaAl_2_O_4_ in the above-mentioned thermal catalytic reactions is under careful investigation, with a focus on the notable synergistic effects of metal–support interfaces in the core@shell structure.

### The broad applicability of the core@shell synthesis platform

We have successfully demonstrated that low-T_Tam_ compounds can facilitate thermal-induced SMSI core@shell construction, and subsequent solid-state reactions for overlayer functionalization enable thermal catalytic applicability. Furthermore, to showcase the general applicability of our proposed approach, we also test other M_T_ and M_AE_CO_3_ combinations in the encapsulable window (shadowed in blue in Fig. 1b). The results have validated the effectiveness of our thermal encapsulation and sintering prevention strategy guided by Tammann temperature (Fig. 4e, Supplementary Figs. 18, 32 and 33, and Supplementary Table 20). More convincingly, the replacement of Al_2_O_3_ with TiO_2_ for the overlayer solid-state reaction enables us to achieve BaTiO_3_ encapsulation on Co NPs (Fig. 4e, Supplementary Fig. 34, and Supplementary Table 20), demonstrating the broad applicability of our strategy across various material matrices (Supplementary Fig. 35).

All these results demonstrate the potential of the Tammann temperature-guided SMSI strategy as a design and synthesis platform for the facile encapsulation of M_T_ NPs. The tunability of both M_T_ core and functional oxide overlayer shows promise for fine-tuning the performance of encapsulated nanocatalysts. It is anticipated that other low-T_Tam_ metal compounds, such as chloride, sulfate, nitrate, and many more, can also be applied to construct size-controlled encapsulation structures for different transition metal NPs, either from in-situ reduction or pre-made metal NPs. Computational calculations can assist with rapid composition selections from a broad material matrix for overlayer solid-state reactions, thus unlocking a wide range of possibilities for metal oxides (or non-oxides) to act as NP protectors/modifiers. Overall, this generalizable thermal encapsulation methodology enables future explorations and predictions for rational and dynamic tuning of nanocatalysts to meet diverse requirements in broad energy and environmental applications.

## Methods

### Synthesis and thermal treatment of catalyst precursors

The precursor mixtures, mM_T_:nM_AE_:2nAl, were synthesised by an automatic pH-controlled co-precipitation method. For a typical preparation procedure, an aqueous solution (50 mL) containing M_T_, M_AE_ and Al cations with a target molar ratio was prepared by dissolving the corresponding metal nitrates (M_T_ = transition metal; M_AE_ = alkaline earth metal) in deionised (DI) water. At room temperature, the mix-metal solution was added dropwise into a stirred tank reactor (capacity of 500 mL~2 L) with a 0.5 M Na_2_CO_3_ (100 mL) solution under feeding rates of 0.1~2.0 mL min^–1^ regulated by a syringe pump. The mixture was stirred vigorously to ensure efficient mixing. At the same time, the pH of the precipitating solution was carefully maintained at a constant by the dropwise addition of a 4.0 M NaOH solution using another syringe pump. The pH value of the solution should be controlled carefully to form M_AE_CO_3_ rather than M_AE_(OH)_2_ precipitates. This research used a pH of 12.5 for obtaining BaCO_3_ and a pH of 9.0 for MgCO_3_. Once all the pre-measured solutions were added to the tank, the liquid was aged for 16 hours, and then the mixture was filtered and washed with DI water until the pH was close to 7.0. The obtained wet cake solid sample was re-dispersed in 200 mL acetone and stirred at room temperature for 2 hours. Following that, the resultant solid was vacuum filtered, washed thoroughly with acetone and dried overnight in a vacuum oven at room temperature. The precursors are named by their nominal mixed-metal ratios, denoted as mM_T_:nM_AE_:2nAl, where m and n represent the nominal molar ratios of M_T_ and M_AE_ in the co-precipitation process, respectively. After getting the mM_T_:nM_AE_:2nAl precipitate, 100 mg of the samples were calcined in the air (denoted as C(temp.)-mM_T_:nM_AE_:2nAl) and thermally treated in H_2_/Ar (5/95, v/v) at specified temperatures to finally form the possible encapsulation structure, obtaining above 50 mg products denoted as C(temp.)-R(temp.)-mM_T_:nM_AE_:2nAl. C(temp.) and R(temp.) indicate the temperatures of air-calcination and H_2_ treatment processes, and the highest temperature (°C) reached in each step is listed in the brackets. Note: For convenience, those samples which have been observed to be successful are denoted as M_T_@M_AE_Al_2_O_4_. Supplementary Fig. 1 is the schematic representation of the steps involved in this synthesis method.

### Material characterizations

Phases and crystallographic structures of the mentioned samples were characterised by synchrotron X-ray diffraction (SXRD), which were collected using the Powder Diffraction (PD) beamline at the Australian Synchrotron (AS), with a photon energy of 21.0005 keV (λ = 0.5904 Å). The microstructure and phase information of the samples were characterised by scanning transmission electron microscopy (STEM) in high-angle annular dark field (HAADF) mode and transmission electron microscopy (TEM), using double-Cs-corrected STEM (Spectra 300, TFS, USA) equipped with Super-X energy-dispersive X-ray spectroscopy (EDX) and STEM/TEM (JEM-2100F, JEOL, Japan) combined with a Gatan Enfina electron spectrometer (USA). X-ray absorption fine structure (XAFS) measurements were performed in fluorescence mode using a Lytel detector at beamline BL01C of Taiwan Light Source, National Synchrotron Radiation Research Center (NSRRC). A Si(111) double crystal monochromator (DCM) was used to scan the photon energy. Direct visualisation of the encapsulation process of Co@BaAl_2_O_4_ was achieved using the in-situ atmospheric STEM study (JEOL JEM-2100F). During the in-situ STEM observation, a Protochips atmosphere gas holder was applied, in which the flowing gas can be injected, and the temperature can be controlled. In this research, 380 Torr H_2_/N_2_ (5/5, v/v) with 0.1 mL/min flow rate were used, and the temperature was set from 25 to 800 °C. In addition, the structure evolution during the H_2_ thermal treatment process was observed by in-situ Raman. About 5 mg samples were loaded into the in-situ Raman cell, with H_2_/Ar (5/95, v/v) passing through the samples. A series of Raman spectra were obtained at different temperatures, from room temperature to 700 °C with an interval of 50 °C. Each temperature was set dwelling for 40 min, and the spectra were collected at the 30^th^ minute to get a steady state. In-situ Raman spectra were recorded on a WITEC confocal microscopy system with a laser diode at 532 nm. A 50× objective lens was used to focus the laser on the sample, and the laser spot size was 1 μm.

### NH_3_ decomposition performance testing method and setup

To measure the catalytic NH_3_ decomposition performance of designed catalysts, inside a quartz tube (diameter of 4.5 mm), c.a. 50 mg sieved (45–80 mesh) sample was sandwiched between two layers of quartz wool with a thermocouple placed in contact with the sample. Then high-purity NH_3_ (≥99.99%) was passed through the catalyst bed with the flow rate controlled by a mass flow controller. The weight hourly space velocity (WHSV) was set as 30,000 mL g_cat_^-1^ h^-1^ at atmospheric pressure. The concentrations of outlet N_2_, H_2_ and NH_3_ after the reaction were measured online by MS (HPR-20 EGA, Hiden), which was equipped with a quadrupole probe and a secondary electron multiplier detector (850 eV). The accuracy of product analyses was further verified by back titration (Supplementary Fig. 24b). The measured temperatures range from 450 to 650 °C with 50 °C as an interval, and a steady state was reached by maintaining each temperature for 60 min.

The NH_3_ conversion was calculated using the following Eq. (1)

1 $${{{{{{\rm{NH}}}}}}}_{3}\,{{{{{\rm{conversion}}}}}}=\frac{{\left[{{{{{{\rm{NH}}}}}}}_{3}\right]}_{{{{{{\rm{inlet}}}}}}}-{\left[{{{{{{\rm{NH}}}}}}}_{3}\right]}_{{{{{{\rm{outlet}}}}}}}}{\left(1+{\left[{{{{{{\rm{NH}}}}}}}_{3}\right]}_{{{{{{\rm{outlet}}}}}}}\right)\times {\left[{{{{{{\rm{NH}}}}}}}_{3}\right]}_{{{{{{\rm{inlet}}}}}}}}\times 100\%$$

where [NH_3_]_inlet_ and [NH_3_]_outlet_ refer to the measured concentrations of NH_3_ fed into and flowing out of the reactor.

The H_2_ production rate, with the unit of mmol H_2_ g_cat_^−1^ min^−1^, was calculated from the NH_3_ conversion (*X*_NH3_) by the Eq. (2) below:

2 $${{{{{{\rm{H}}}}}}}_{2}\,{{{{{\rm{production\; rate}}}}}}=\frac{{{{{{\rm{WHSV}}}}}}\times {X}_{{{{{{{\rm{NH}}}}}}}_{3}}\times 1.5}{{{{{{{\rm{V}}}}}}}_{{{{{{\rm{m}}}}}}}\times 60}$$

where WHSV is the weight hourly space velocity (30,000 mL g_cat_^−1^ h^−1^), *X*_NH3_ is the conversion of NH_3_, and V_m_ is the molar volume of gas at 25 °C and 1 atm (24 mL mmol^−1^).

### Supplementary information

Supplementary Information

Peer Review File

Description of Additional Supplementary Files

Supplementary Movie 1

Supplementary Movie 2

Supplementary Movie 3

### Source data

Source Data

## Data Availability

Source data are provided with this paper. The authors declare that the data supporting the findings of this study are available within the paper, its supplementary information files, and the Figshare repository (10.6084/m9.figshare.24647937). Source data are provided with this paper.
