# Supplementary material for: Synthesis of core@shell catalysts guided by Tammann temperature
Source: Nat Commun. 2024 Jan 10;15:420. doi: 10.1038/s41467-024-44705-5 (PMC10782006; doi:10.1038/s41467-024-44705-5)
Supplement: Supplementary file 1 — Supplementary Information [file 41467_2024_44705_MOESM1_ESM.pdf]

Supplementary Information for

Synthesis of core@shell catalysts guided by Tammann temperature

Pei Xiong†, Zhihang Xu†, Tai-Sing Wu, Tong Yang, Qiong Lei, Jiangtong Li, Guangchao Li, Ming Yang, Yun-Liang Soo, Robert David Bennett, Shu Ping Lau, Shik Chi Edman Tsang*, Ye Zhu* and Molly Meng-Jung Li*

*Corresponding author: edman.tsang@chem.ox.ac.uk (S.C.E.T); yezhu@polyu.edu.hk (Y.Z.); molly.li@polyu.edu.hk (M.M.J.L).

†These authors contributed equally to this work: Pei Xiong, Zhihang Xu.

This Supplementary Information includes:

List of Abbreviations
Supplementary Methods
Supplementary Figures 1 to 35
Supplementary Tables 1 to 20

List of Abbreviations

T_{Tam}	Tammann temperature
T_{red}	Reduction temperature
T_{dec}	Decomposition temperature
M_T	Transition metal
NPs	Nanoparticles
M_TO_x	Transition metal oxide
M_{AE}	Alkaline earth metal
$M_{AE}CO_3$	Alkaline earth metal carbonate
$(M_{AE})_xAl_yO_z$	Alkaline earth metal aluminates
SMSI	Strong metal-support interaction
XRD	X-ray diffraction
STEM	Scanning transmission electron microscopy
TEM	Transmission electron microscopy
EDS	Energy dispersive X-ray spectroscopy
EELS	Electron energy loss spectroscopy
FFT	Fast Fourier transform
XANES	X-ray absorption near edge structure
EXAFS	Extended X-ray absorption fine structure
SXRD	Synchrotron X-ray diffraction
XPS	X-ray photoelectron spectroscopy
EPR	Electron paramagnetic resonance
WHSV	Weight hourly space velocity

Supplementary Methods

I. Materials and instruments

Iron nitrate nonahydrate ($Fe(NO_3)_3{\cdot}9H_2O$, 99.0%), cobalt nitrate hexahydrate ($Co(NO_3)_2{\cdot}6H_2O$, 99.0%), nickel nitrate hexahydrate ($Ni(NO_3)_2{\cdot}6H_2O$, 99.0%), copper nitrate ($Cu(NO_3)_2$, 99.0%), magnesium carbonate ($MgCO_3$, AR), calcium carbonate ($CaCO_3$, AR), strontium carbonate ($SrCO_3$, AR), barium carbonate ($BaCO_3$, AR), sodium carbonate (Na_2CO_3, 99.0%) and sodium hydroxide (NaOH, 96.0%) were purchased from Shanghai Aladdin Bio-Chem Technology Co., Ltd. Barium nitrate ($Ba(NO_3)_2$, 99.0%) and aluminium nitrate nonahydrate ($Al(NO_3)_3{\cdot}9H_2O$, 99.0%) were purchased from Sigma-Aldrich. Nano iron oxide (Fe_2O_3, 99.0%), nano cobalt oxide (Co_3O_4, 99.0%), nano nickel oxide (NiO, 99%) and nano copper oxide (CuO, 99%) were purchased from Shanghai Macklin Biochemical Co., Ltd. All chemicals with analytical-grade purity were used directly without further treatment unless otherwise noted. All solutions were prepared with deionised (DI) water. Anhydrous grade ammonia (NH_3, $\geq$ 99.99%) and H_2/Ar (5/95, v/v) were purchased from Scientific Gas Engineering Co., Ltd.

II. Synthesis of catalyst precursors mM_T:nM_{AE}:2nAl: co-precipitation synthesis method and thermal treatment

The precursor mixtures, mM_T:nM_{AE}:2nAl, were synthesised by an automatic pH-controlled co-precipitation method. For a typical preparation procedure, an aqueous solution (50 mL) containing M_T, M_{AE} and Al cations with a target molar ratio was prepared by dissolving the corresponding metal nitrates (M_T = transition metal; M_{AE} = alkaline earth metal) in deionised (DI) water. Specifically, for each material combination, the precursor solution concentration is as follows: (i) 40Co:20Ba:40Al – 0.10 M $Co(NO_3)_2$ + 0.05 M $Ba(NO_3)_2$+ 0.10 M $Al(NO_3)_3$; (ii) 40Ni:20Ba:40Al – 0.10 M $Ni(NO_3)_2$ + 0.05 M $Ba(NO_3)_2$+ 0.10 M $Al(NO_3)_3$; (iii) 40Cu:20Mg:40Al – 0.10 M $Cu(NO_3)_2$ + 0.05 M $Mg(NO_3)_2$+ 0.10 M $Al(NO_3)_3$; (iv)

20Co:20Ba:60Ti – 0.05 M $Co(NO_3)_2$ + 0.05 M $Ba(NO_3)_2$+ 0.15 M $TiCl_3$. At room temperature, the mix-metal solution was added dropwise into a stirred tank reactor (capacity of 500 mL ~ 2 L) with 0.5 M Na_2CO_3 (100 mL) solution under feeding rates of 0.1 ~ 2.0 mL min^{-1} regulated by a syringe pump. The mixture was stirred vigorously to ensure efficient mixing. At the same time, the pH of the precipitating solution was carefully maintained at a constant by the dropwise addition of a 4.0 M NaOH solution using another syringe pump. The pH value of the solution should be controlled carefully to form $M_{AE}CO_3$ rather than $M_{AE}(OH)_2$ precipitates. This research used a pH of 12.5 for obtaining $BaCO_3$ and a pH of 9.0 for $MgCO_3$. Once all the pre-measured solutions were added to the tank, the liquid was aged for 16 hours, and then the mixture was filtered and washed with DI water until the pH was close to 7.0. The obtained wet cake solid sample was re-dispersed in 200 mL acetone and stirred at room temperature for 2 hours. Following that, the resultant solid was vacuum filtered, washed thoroughly with acetone and dried overnight in a vacuum oven at room temperature. The precursors are named by their nominal mixed-metal ratios, donated as mM_T:nM_{AE}:2nAl, where m and n represent the nominal molar ratios of M_T and M_{AE} in the co-precipitation process, respectively. After getting the mM_T:nM_{AE}:2nAl precipitate, 100 mg of the samples were calcined in the air (donated as C(temp.)-mM_T:nM_{AE}:2nAl) and thermally treated in H_2/Ar (5/95, v/v) at specified temperatures to finally form the possible encapsulation structure, obtaining 50 to 70 mg products donated as C(temp.)-R(temp.)-mM_T:nM_{AE}:2nAl. C(temp.) and R(temp.) indicate the temperatures of air-calcination and H_2 treatment processes, and the highest temperature (°C) reached in each step is listed in the brackets. Note: For convenience, those samples which have been observed to be successful are donated as M_T@$M_{AE}Al_2O_4$. Supplementary Fig. 1 is the schematic representation of the steps involved in this synthesis method.

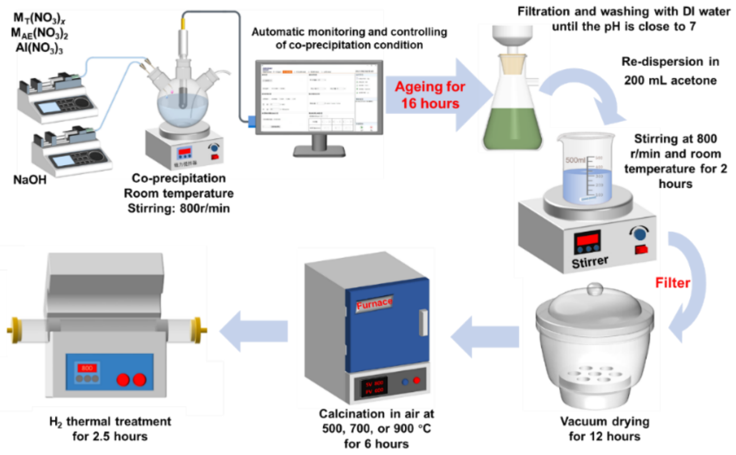

Supplementary Figure 1. Precursors preparation via co-precipitation synthesis method and thermal treatment. Schematic representation of the mM_T:nM_{AE}:2nAl precursor synthesis procedure and subsequent thermal treatment process.

III. Measurement of $M_{AE}CO_3$ decomposition temperature (T_{dec})

Temperature-programmed decomposition measurements of commercial alkaline earth metal carbonates ($M_{AE}CO_3$, M_{AE} = Mg, Ca, Sr, and Ba) were conducted using a tube furnace (GSL-1100X, Kejing) combined with a quadrupole mass spectrometer (MS, HPR-20 EGA, Hiden). Inside the quartz tube, c.a. 50 mg of the $M_{AE}CO_3$ sample was sandwiched between two layers of quartz wool with a thermocouple in contact with the sample. The quartz tube was then inserted into the tube furnace for argon (Ar) pretreatment, with the Ar flow rate of 10 mL min^{-1}. The temperature was raised from 25 to 50 °C with a ramp rate of 5 °C min^{-1}, then held at 50 °C for 30 min. The Ar pretreatment cleaned the catalyst surface by removing adsorbed

ambient gas molecules. After the pretreatment, to decompose $M_{AE}CO_3$, H_2/Ar (5/95, v/v) gas flowed through the quartz tube with the flow rate of 10 mL min^{-1}, and the temperature was raised from 25 to 800 °C (950 °C for $BaCO_3$ and $BaCO_3$ + Co_3O_4) with a ramp rate of 5 °C min^{-1}. The outlet gas products, including H_2O (m/z = 18), CO (m/z = 28) and CO_2 (m/z = 44), were recorded by MS as a function of temperature.

IV. Measurement of M_TO_x reduction temperature (T_{red})

Temperature-programmed reduction measurements of nanosized commercial metal oxides (M_TO_x, M_T = Fe, Co, Ni, Cu) were conducted using a tube furnace (GSL-1100X, Kejing) combined with a quadrupole MS (HPR-20 EGA, Hiden). Inside the quartz tube, c.a. 50 mg of the M_TO_x sample was sandwiched between two layers of quartz wool with a thermocouple in contact with the sample. The quartz tube was inserted into the tube furnace for Ar pretreatment, with the Ar flow rate of 10 mL min^{-1}. The temperature was raised from 25 to 50 °C with a ramp rate of 5 °C min^{-1}, then held at 50 °C for 30 min. The Ar pretreatment cleaned the catalyst surface by removing adsorbed ambient gas molecules. After the pretreatment, to reduce M_TO_x, H_2/Ar (5/95, v/v) gas flowed through the quartz tube with the flow rate of 10 mL min^{-1}, and the temperature was raised from 25 to 800 °C (950 °C for Fe_2O_3) with a ramp rate of 5 °C min^{-1}. M_TO_x was reduced to M_T NPs, with H_2O produced during the heating treatment. The gas product H_2O (m/z = 18) were recorded by MS as a function of temperature.

V. Theoretical estimation of solid-state reaction energy

Reaction energies for forming different alkaline earth metal aluminates ($M_{AE}Al_2O_4$) from $M_{AE}CO_3$ and Al_2O_3 were estimated using the reaction calculator module in the Materials Project. Details can be found in the descriptions of Supplementary Fig. 9.

VI. Material characterisations

(1) X-ray diffraction (XRD)

Phases and crystallographic structures of the mentioned samples were characterised by powder X-ray diffraction (XRD) using a parallel-beam XRD instrument (Rigaku SmartLab 9kW - Advance, with Cu Kα of wavelength 1.5406 Å). For the measurements of the reduced and post-reaction samples, the samples were protected in the reaction tube with both ends sealed using closed ball valves, and were directly transferred into the inert gas glove box. In the inert gas glove box, the samples were taken from the reaction tube and loaded into a quartz holder, which was then covered by Kapton film (Supplementary Fig. 2). In this air-tight XRD holder, the air-sensitive samples were kept isolated from the air and can be directly measured by the XRD facility.

The crystallite size (D) was estimated by XRD using the Debye-Scherrer equation ($D = \frac{k\lambda}{\beta\cos\theta}$), where k is the shape coefficient for the reciprocal lattice point and shape coefficient for crystal in the direct space (k = 0.9), λ is the wavelength of the incident radiation, β is the full width at half-maximum (FWHM) of the peak, and θ is the Bragg angle.

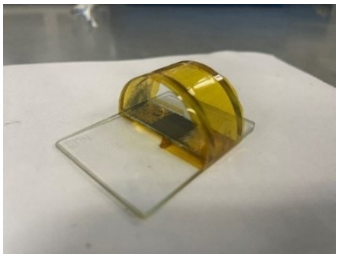

Supplementary Figure 2. XRD measurement of air-sensitive samples. Air-tight XRD holder for air-sensitive samples.

(2) Synchrotron X-ray diffraction (SXRD)

SXRD patterns were collected using the Powder Diffraction (PD) beamline at the Australian Synchrotron (AS), which was set to provide a photon energy of 21.0005 keV (λ = 0.5904 Å). Each capillary was mounted on a crystallographic goniometer head and rotated at 15 rpm. Patterns were measured using Debye-Scherrer geometry with a goniometer diameter = 152 cm, over the 2θ range of 1.01398° to 80.8344° and with an effective step size of 0.00375°. The SXRD incident and diffracted beams were fully polarized.

Each sample was measured twice using 450-second acquisitions, with the Mythen detector being offset by 0.5° for the second data acquisition. The two off-set patterns were merged using the program PDViPeR. An additional pattern was measured for a NIST SRM-660b LaB_6 powder under the same conditions to determine the instrument wavelength, the $2\theta_0$ correction and the instrument FWHM widths for the Bragg peaks.

The diffraction patterns were analysed by Rietveld refinement methods based on TOPAS (V6) to obtain structural details. The Thompson-Cox-Hastings pseudo-Voigt peak function was applied to describe the diffraction peaks. The scale factor and lattice parameters were allowed to be refined for all the diffraction patterns. The quality of the Rietveld refinements of synchrotron data has been assured with a low goodness-of-fit (GoF) factor, a low weighted profile factor (Rwp) and a well-fitted pattern with an acceptable temperature factor (Beq) within experimental errors.

(3) Transmission electron microscopy (TEM)

The microstructure and phase information of the samples were characterised by scanning transmission electron microscopy (STEM) in high-angle annular dark field (HAADF) mode and transmission electron microscopy (TEM), using double-Cs-corrected STEM (Spectra 300, TFS, USA) equipped with Super-X energy-dispersive X-ray spectroscopy (EDS) and STEM/TEM (JEM-2100F, JEOL, Japan) combined with a Gatan Enfina electron spectrometer (USA). TEM samples were prepared by sonicating a suitable amount of material in 1 mL ethanol for 180 seconds before the dropwise addition of the solution onto the copper grids. For the reduced and post-reaction samples, the samples were protected in the reaction tube with both ends sealed using closed ball valves and were directly transferred into the inert gas glove box, where the samples can be extracted from the reaction tube and dispersed in ethanol solution for further deposition onto copper grids.

(4) Scanning electron microscopy with energy dispersive X-ray analyses (SEM-EDS)

Scanning electron microscopy (SEM) images were recorded on a Tescan VEGA3 Model GMH microscope at an accelerating voltage of 5 kV. The atomic ratios for metal elements including Co, Ba, and Al of mentioned samples were measured by EDS.

(5) Electron paramagnetic resonance (EPR)

The electron paramagnetic resonance (EPR) analyse was performed by a Bruker EMX Plus spectrometer equipped with a dual-mode cavity (ER 4116DM). The microwave frequency and power were 9.83 GHz and 3.17 mW, respectively. The weight of the sample was 50.0 mg.

(6) X-ray absorption fine structure (XAFS)

X-ray absorption fine structure (XAFS) measurements were performed in fluorescence mode using a Lytel detector at beamline BL01C of Taiwan Light Source, National Synchrotron Radiation Research Center (NSRRC). A Si(111) double crystal monochromator (DCM) was used to scan the photon energy. To ascertain the reproducibility of the experimental data, at least two scans were collected and compared for each sample. X-ray absorption near edge

structure (XANES) data were background-subtracted and normalised using the AUTOBK routine in Athena software. Quantitative information on the radial distribution of neighbouring atoms surrounding Co and Ba atoms was derived from the extended absorption fine structure (EXAFS) data. An established data reduction method was used to extract the EXAFS χ-functions from the raw experimental data using the IFEFFIT software. For the wavelet transform, k^3-weighted EXAFS spectra were used. The Morlet wavelet mother function was used for the wavelet transform (WT) of all spectra within the k-range of 3 ~ 13 $Å^{-1}$ for Co K-edge and 3 ~ 9 $Å^{-1}$ for Ba L_3-edge, respectively. The selection of this wavelet was governed by the fast oscillatory part localised in a Gaussian envelope, making its real and imaginary parts similar to an EXAFS spectrum.

(7) X-ray photoelectron spectroscopy (XPS)

X-ray photoelectron spectroscopy (XPS) experiments were carried out using a Thermo Scientific Nexsa instrument. The instrument was equipped with an electron flood and scanning ion gun. The adventitious carbonaceous C1*s* line (284.8 eV) was used to calibrate the binding energy (BE). The XPS spectra were deconvoluted using Gaussian-Lorentzian functions after the Shirley background subtraction, with the integrated peak areas being used to estimate the surface chemical compositions.

(8) In-situ Raman Spectroscopy

During a typical in-situ Raman measurement, c.a. 5 mg samples were loaded into the in-situ Raman cell, with H_2/Ar (5/95, v/v) passing through the samples. A series of Raman spectra were obtained at different temperatures, from room temperature to 700 °C with an interval of 50 °C, to investigate the phase transformation during the H_2 thermal treatment process. Each temperature was set dwelling for 40 min, and the spectra were collected at the 30th min to get a steady state. In-situ Raman spectra were recorded on a WITEC confocal microscopy system with a laser diode at 532 nm. A 50× objective lens was used to focus the laser on the sample, and the laser spot size was 1 μm. The Raman measurements were performed under an accumulation time of 60 s and an accumulation number of 10 times by illuminating 3 mW laser power. Supplementary Fig. 3 shows the in-situ Raman setup. Equipped with a quadrupole MS (HPR-20 EGA, Hiden), the composition and concentration of the outlet gas were analysed, with H_2 (m/z = 2), H_2O (m/z = 18) and CO (m/z = 28) recorded as a function of temperature.

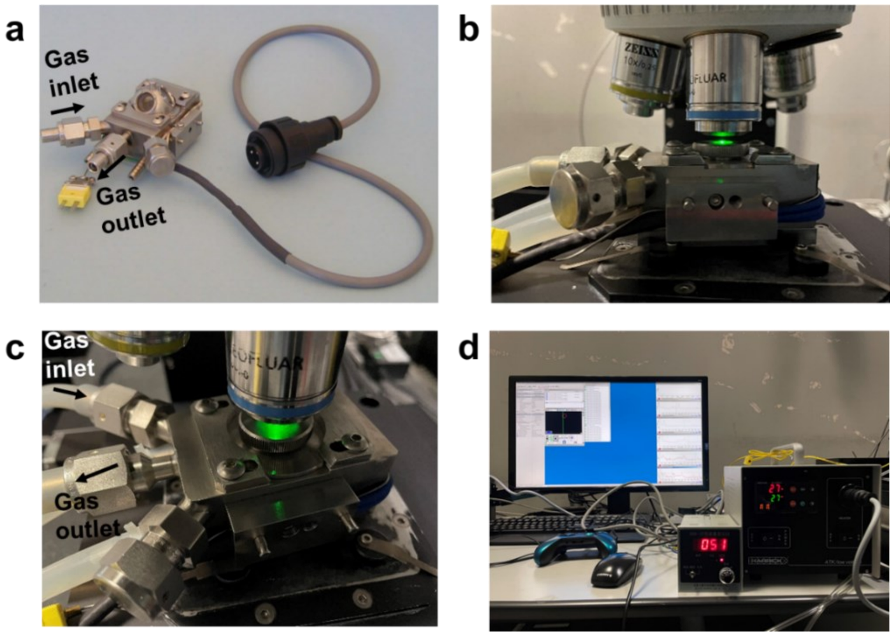

Supplementary Figure 3. In-situ Raman setup. (a) in-situ Raman high-temperature reaction chamber (Harrick Scientific Products Inc.), with gas flow directions marked. **(b-c)** Experimental setup for the in-situ example showing the gas cell mounted on the stage of a Raman imaging microscope. **(d)** Temperature and flow control units of the in-situ Raman setup.

(9) In-situ STEM

Direct visualisation of the encapsulation process of Co@$BaAl_2O_4$ was achieved using the in-situ atmospheric STEM study (JEOL JEM-2100F). During the in-situ STEM observation, a Protochips atmosphere gas holder was applied (Supplementary Fig. 4a), in which the flowing gas can be injected, and the temperature can be controlled. In this experiment: 380 Torr H_2/N_2 (5/5, v/v) with 0.1 mL/min flow rate were used, and the temperature was set from 25 to 800 °C.

The effect of the electron beam was carefully evaluated by comparing the sample state in the observation region with that out of the observation region. The initial precursor, C(500)-40Co:20Ba:40Al, was firstly prepared on the bottom cell and characterised by STEM and EDS before heating the in-situ cell. The bottom cell was then assembled in the in-situ holder, followed by injecting an H_2/N_2 mixture gas into it. The temperature was gradually increased to 300, 400, 500, 600, 700, and 800 °C at a rate of 20 °C/min. Each target temperature was kept for 30 min for a thorough reaction. Supplementary Fig. 4 shows the in-situ STEM setup. The introduction of gas into the vacuum environment of the TEM is made possible by injecting and sealing the gas in the space between two windowed silicon slabs (the E-chips product from Protochips, shown in Supplementary Fig. 4b). The windowed slabs have notches near their centres and are covered with amorphous silicon nitride thin films of thickness 80 nm that serve as windows[1].

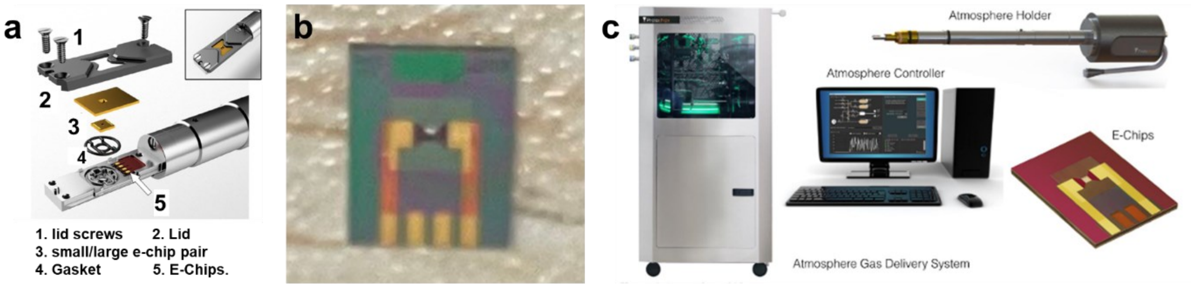

Supplementary Figure 4. In-situ STEM setup. (a) Schematic showing the assembly of the Protochips Atmosphere gas holder. **(b)** An E-chip used in the Protochips Atmosphere gas holder. **(c)** Atmosphere system for the in-situ STEM experiment.

VII. NH_3 decomposition performance testing method and setup

Supplementary Fig. 5 is the schematic of the NH_3 decomposition performance testing setup. To measure the catalytic NH_3 decomposition performance of designed catalysts, inside a quartz tube (diameter of 4.5 mm), c.a. 50 mg sieved (45-80 mesh) sample was sandwiched between two layers of quartz wool with a thermocouple placed in contact with the sample. Then high-purity NH_3 (≥ 99.99%) was passed through the catalyst bed with the flow rate controlled by a mass flow controller. The weight hourly space velocity (WHSV) was set as 30,000 mL g_{cat}^{-1} h^{-1} at atmospheric pressure. The concentrations of outlet N_2, H_2 and NH_3 after the reaction were measured online by MS (HPR-20 EGA, Hiden), which was equipped with a quadrupole probe and a secondary electron multiplier detector (850 eV). The accuracy of product analyses was further verified by back titration (Supplementary Fig. 24b). The experimental details of the back titration methods have been reported in previous studies by

our group[2]. The measured temperatures range from 450 to 650 °C with 50 °C as an interval, and a steady state was reached by maintaining each temperature for 60 min.

The NH_3 conversion was calculated using the following Equation (1)

$$NH_3 \text{ conversion } = \frac{[NH_3]_{inlet} - [NH_3]_{outlet}}{(1+[NH_3]_{outlet}) \times [NH_3]_{inlet}} \times 100\% \quad (1)$$

where $[NH_3]_{inlet}$ and $[NH_3]_{outlet}$ refer to the measured concentrations of NH_3 fed into and flowing out of the reactor[3].

The H_2 production rate, with the unit of mmol H_2 g_{cat}^{-1} min^{-1}, was calculated from the NH_3 conversion (X_{NH3}) by the Equation (2) below[4]:

$$H_2 \text{ production rate} = \frac{WHSV \cdot X_{NH_3} \cdot 1.5}{V_m \times 60} \quad (2)$$

where WHSV is the weight hourly space velocity (30,000 mL g_{cat}^{-1} h^{-1}), X_{NH3} is the conversion of NH_3, and V_m is the molar volume of gas at 25 °C and 1 atm (24 mL $mmol^{-1}$).

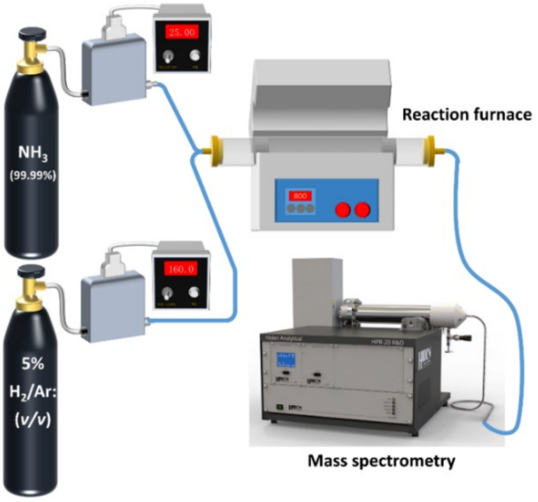

Supplementary Figure 5. Schematic of the NH_3 decomposition performance testing setup.

VIII. Methane dry reforming performance testing

Catalytic methane (CH_4) dry reforming activity testing was performed under atmospheric pressure in a fixed-bed quartz reactor (5 mm inner diameter). Before each reaction, 100 mg of catalyst was placed in the reactor tube between quartz wool plugs and reduced in situ at 700 °C under flowing H_2/Ar (5/95, v/v, 60 mL min^{-1}) for 2.5 h. Dry reforming of CH_4 was then undertaken at 750 °C using a mixture of CO_2/CH_4 in Ar (45/45/10, v/v/v, 53 mL min^{-1} total flow rate) to generate a weight hourly space velocity (WHSV) of 31,765 mL g_{cat}^{-1} h^{-1}. Reactant and product concentrations were analysed online by a MS (QGA, Hiden) equipped with a quadrupole probe and a secondary electron multiplier detector. CH_4 and CO_2 conversions were calculated respectively according to the following Equations (3) and (4):

$$CH_4 \text{ conversion } = \frac{F_{inlet} \cdot [CH_4]_{inlet} - F_{outlet} \cdot [CH_4]_{outlet}}{F_{inlet} \cdot [CH_4]_{inlet}} \times 100\% \quad (3)$$

$$CO_2 \text{ conversion } = \frac{F_{inlet} \cdot [CO_2]_{inlet} - F_{outlet} \cdot [CO_2]_{outlet}}{F_{inlet} \cdot [CO_2]_{inlet}} \times 100\% \quad (4)$$

Where $[CH_4]$ and $[CO_2]$ are the respective mole fractions of CH_4 and CO_2 in the stream, and F_{inlet} and F_{outlet} represent the total gas flow rate (mL min^{-1}) of the inlet and outlet, respectively. The selectivity of H_2 and CO are presented by the following Equations (5) and (6):

$$H_2 \text{ selectivity } = \frac{\text{moles of } H_2 \text{ produced}}{2 \times \text{moles of } H_2 \text{ converted}} \times 100\% \tag{5}$$

$$CO \text{ selectivity } = \frac{\text{moles of } CO \text{ produced}}{[\text{moles of } CH_4 + \text{moles of } CO_2] \text{ converted}} \times 100\% \tag{6}$$

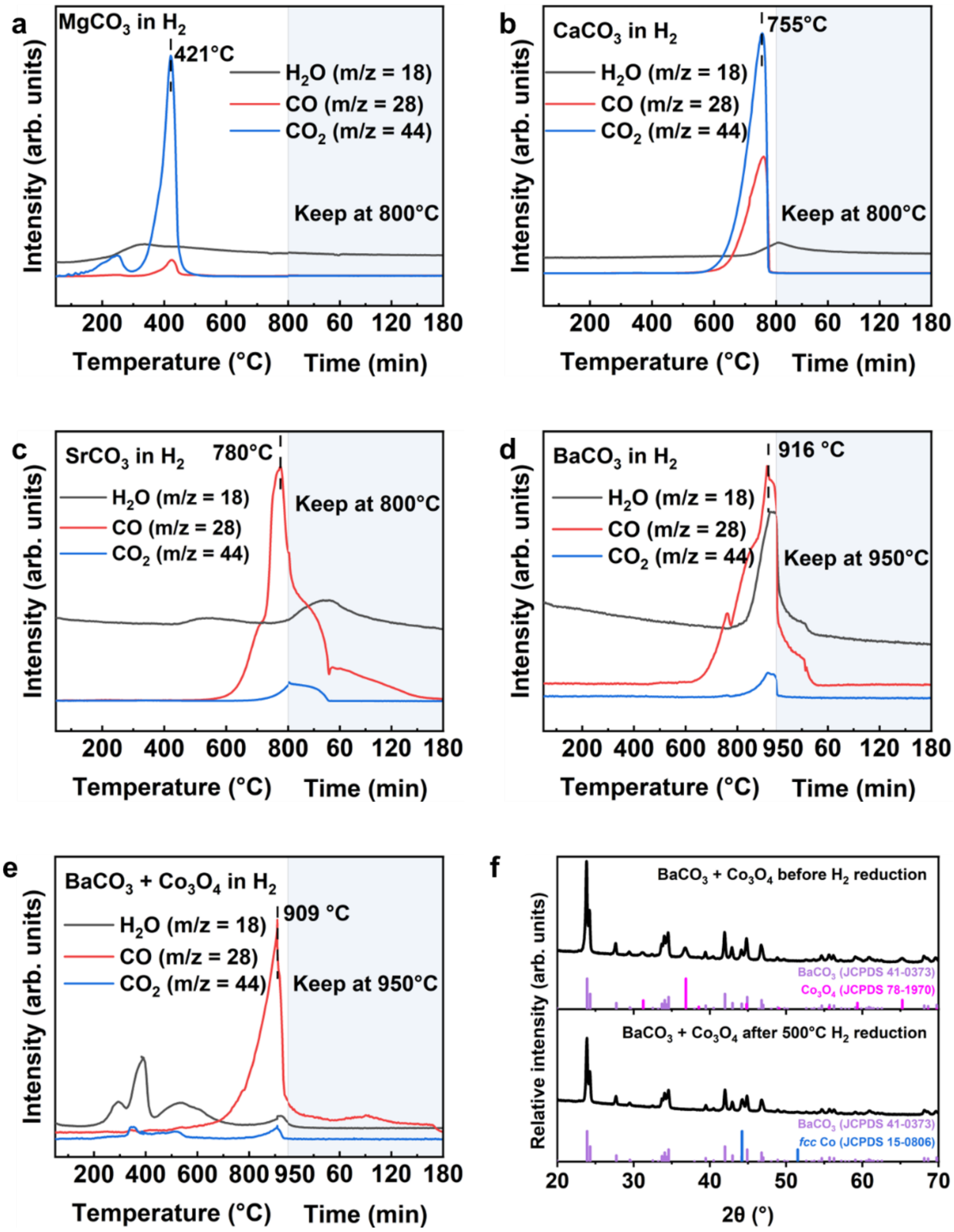

Supplementary Figure 6. Determination of the decomposition temperature (T_{dec}) of $M_{AE}CO_3$. MS signals during the H_2 temperature-programmed decomposition of **(a)** $MgCO_3$, **(b)** $CaCO_3$, **(c)** $SrCO_3$, **(d)** $BaCO_3$, and **(e)** $BaCO_3$ + Co_3O_4. When it reached 800 °C, the temperature was kept at 800 °C for 3 hours (950 °C for $BaCO_3$). The unshadow areas (x = 50 to 800 °C) show the MS signals as a function of temperature. The blue-shadow areas (x = 0 to 180 min) show the MS signals as a function of the retention time at 800°C (950 °C for $BaCO_3$, and $BaCO_3$ + Co_3O_4). The maximum of each decomposition profile is determined as the corresponding $T_{dec}(M_{AE}CO_3)$[5,6]. **(f)** XRD patterns of $BaCO_3$ + Co_3O_4 mixture precursors before reduction (upper panel) and after 500 °C H_2 reduction (lower panel). Source data are provided as a Source Data file.

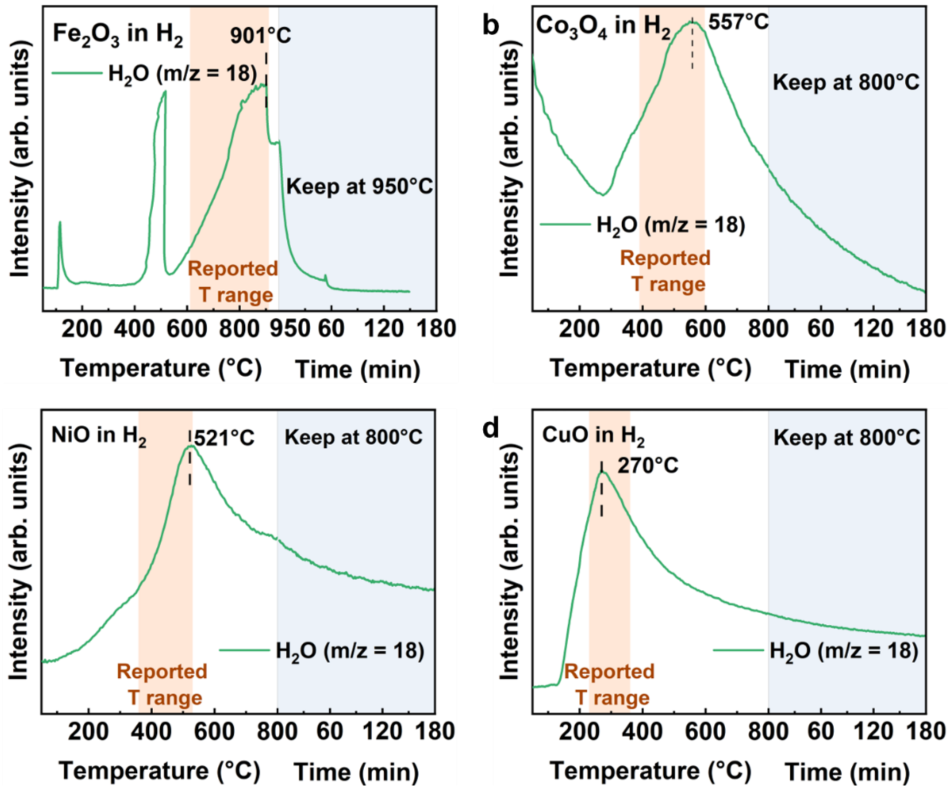

Supplementary Figure 7. Determination of the reduction temperature (T_{red}) of M_TO_x. MS signals during the H_2 temperature-programmed reduction of **(a)** Fe_2O_3, **(b)** Co_3O_4, **(c)** NiO, and **(d)** CuO. When it reached 800 °C, the temperature was kept at 800 °C for 3 hours (950 °C for Fe_2O_3). The unshadow areas (x = 50 to 800 °C) show the MS signals as a function of temperature. The blue-shadow areas (x = 0 to 180 min) show the MS signals as a function of the retention time at 800°C (950 °C for Fe_2O_3). The orange-shadow areas are the literature-reported H_2 temperature-programmed reduction temperature ranges, indicating that our measured values are all in reasonable ranges according to previous reports (Detailed reported data can be found in Supplementary Table 2). The maximum of each reduction profile is determined as the corresponding $T_{red}(M_TO_x)$[5,6]. Source data are provided as a Source Data file.

Supplementary Table 1

Melting points (T_{melt}) and Tammann temperatures (T_{Tam}) of $M_{AE}CO_3$ (M_{AE} = Mg, Ca, Sr, Ba) and M_T (M_T = Fe, Co, Ni, Cu) retrieved from literature. The experimental decomposition temperatures (T_{dec}) of $M_{AE}CO_3$ and reduction temperatures (T_{red}) of M_TO_x.

	T_{melt}($M_{AE}CO_3$ or M_T), °C	T_{Tam}($M_{AE}CO_3$ or M_T)*, °C	T_{dec}($M_{AE}CO_3$), °C (under H_2/Ar)	T_{red}(M_TO_x), °C (under H_2/Ar)
$MgCO_3$	350	38 [7]	421	
$CaCO_3$	1339	533 [8]	755	
$SrCO_3$	1494	610 [8]	780	
$BaCO_3$	811	269 [9]	916	
Fe	1535	631 [10]		
Co	1480	604 [10]		
Ni	1452	590 [10]		
Cu	1083	405 [10]		
Fe_2O_3				901
Co_3O_4				557
NiO				521
CuO				270

*The crystalline lattice of a solid compound maintains constant vibrational motion at normal room temperature. When the temperature increases, the ions' motion amplitude increases until they reach the melting point and transition into a liquid phase. Tammann temperature (T_{Tam}) is an absolute temperature at which atoms in the crystal lattice of a solid bulk material become 'loosened' and, therefore, more reactive or susceptible to diffusion by other molecules. T_{Tam} is reported as approximately half of a compound's absolute melting point (T_{melt})[11].

Supplementary Table 2

Reduction temperatures of M_TO_x to M_T NPs in H_2 temperature-programmed reduction spectra retrieved from literature.

**Metals**	**T_{peak}, °C**	**Sample weight, mg**	**Gas**	**Heating rate, °C/min**	**References**
Fe_2O_3 to Fe	600	20	5%H_2 + Ar	10	12
	610	/	50%H_2 + N_2	10	13
	650	/	/	/	14
	660	30	10%H_2 + Ar	2	15
	690	50	5%H_2 + Ar	10	16
	700	25	5.8%H_2 + Ar	5	17
	710	50	10%H_2 + Ar	10	18
	740	50	20%H_2 + N_2	10	19
	780	50	5%H_2 + Ar	10	20
	820	50	5%H_2 + N_2	5	21
	900	50	10%H_2 + He	10	22
Co_3O_4 to Co	390		5%H_2 + He	10	23
	400		5%H_2 + Ar	10	24
	402	50	10%H_2 + Ar	10	25
	410	10	2%H_2 + Ar	10	26
	420	20	5%H_2 + Ar	10	12
	420		5%H_2 + Ar	10	27
	450	50	10%H_2 + N_2	10	28
	470	100	5%H_2 + He	10	29
	485	60	5%H_2 + Ar	10	30
	490	100	10%H_2 + Ar	10	31
	493	20	5%H_2 + Ar	5	32
	500	100	5%H_2 + Ar	10	33
	542		10%H_2 + Ar	5	34
	547	100	10%H_2 + Ar	10	35
	597	100	10%H_2 + Ar	10	36
NiO to Ni	358		10%H_2 + Ar	10	37
	359		5%H_2 + He	10	23
	375	20	10%H_2 + Ar	10	38

Metals	T_{peak}, °C	Sample weight, mg	Gas	Heating rate, °C/min	References
	380	10	5%H_2 + Ar	5	39
	380	100	5%H_2 + Ar	10	40
	400	20	5%H_2 + Ar	10	12
	420	100	5%H_2 + Ar	10	41
	450		10%H_2 + Ar		42
	500	50	5%H_2 + He	10	43
	530	50	5%H_2 + Ar	10	44
	540	50	5%H_2 + He	10	45
CuO to Cu	230		5%H_2 + He		23
	240		5%H_2 + Ar	10	46
	240		5%H_2 + N_2		47
	240				48
	245	50	10%H_2 + Ar	10	49
	257	30	5%H_2 + N_2	10	50
	260	200	10%H_2 + N_2	10	51
	299	100	10%H_2 + N_2	10	52
	313		5%H_2 + Ar		53
	320	50	9.9%H_2 + He	10	54
	320	10	5%H_2 + Ar	5	39
	320		5%H_2 + Ar	10	55
	326	30	5%H_2 + N_2	10	50
	340		10%H_2 + Ar	10	56
	350	80	5%H_2 + He	5	57
	356	10	2%H_2 + Ar	10	26
	360	30	5%H_2 + N_2	10	50

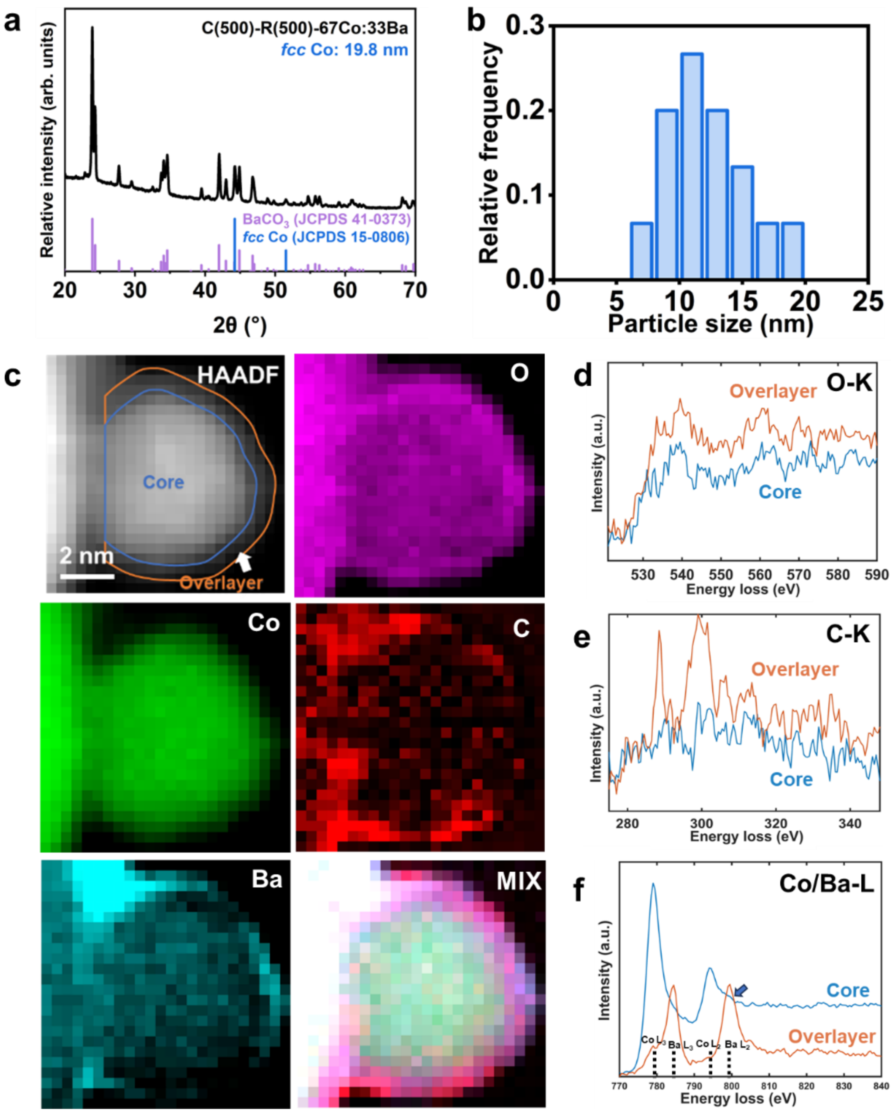

Supplementary Figure 8. Proof-of-concept demonstration by constructing Co@$BaCO_3$ encapsulation configuration. (a) XRD pattern of C(500)-R(500)-67Co:33Ba, **(b)** Co NP size distribution measured from low magnification TEM images of C(500)-R(500)-67Co:33Ba. **(c)** Additional set of STEM and EELS element distribution maps of C(500)-R(500)-67Co:33Ba, including Co, Ba, O and C elements. **(d-f)** The EELS spectra of O-K, C-K, and Co-L, Ba-L edges extracted from the core and the overlayer. Source data are provided as a Source Data file.

Supplementary Table 3

Estimation of Co NP crystallite size in C(500)-R(500)-67Co:33Ba

Sample	Phase (Peak selected for crystallite size calculation)	Co NP crystallite size from the Debye-Scherrer equation		Co NP size distribution from TEM, nm
		FWHM, °	Co NP crystallite size, nm	
C(500)-R(500)-67Co:37Ba	Co (111)	0.434	19.8	10 ~ 15

67Co:33Ba precursor was synthesized by co-precipitating $Co(NO_3)_2$ and $Ba(NO_3)_2$ solutions in $BaCO_3$ solutions, followed by calcination in air at 500 °C and thermal treatment in

H_2 at 500 °C. The final obtained product was donated as C(500)-R(500)-67Co:33Ba. Supplementary Fig. 8a shows the XRD pattern of C(500)-R(500)-67Co:33Ba, in which $BaCO_3$ and fcc Co are observed, indicating that Co_3O_4 has been reduced to fcc Co while keeping $BaCO_3$ undecomposed.

Apart from the EELS mapping of one core@shell particle in Fig. 1c, to provide more convincing evidence, we have included another set of EELS mapping data for the SMSI Co@$BaCO_3$ (Supplementary Fig. 8c). The color-coded maps clearly illustrate the distribution of Ba, O, and C elements in the overlayer surrounding the core Co particle. To further validate the presence of C and O elements in the covering overlayer of the core Co NPs, we extracted EELS spectra from the marked locations (Supplementary Fig. 8c) in both the core and the shell, as shown in Supplementary Fig. 8d-e. These extracted spectra, including the O K-edge and C K-edge spectra, demonstrate the presence of both O and C elements in both the core covering overlayer and the shell regions. The observed O and C elements are from $BaCO_3$ structure, as the XRD data in Supplementary Fig. 8a indicate the presence of $BaCO_3$ and fcc Co as the only observed compounds in this sample. Additionally, as depicted in Supplementary Fig. 8f for the Ba L-edge and Co L-edge, it is evident that the core exhibits a high intensity Co signal with a weak Ba signal represented by a small hump observed beside the Co L_2-edge, as indicated by the arrow. These findings collectively confirm the presence of a $BaCO_3$ overlayer encapsulating the Co NPs.

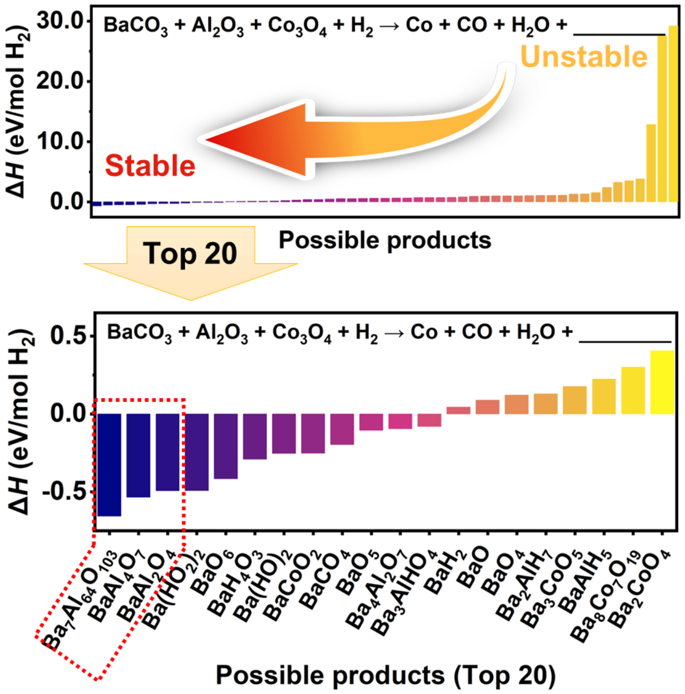

Supplementary Figure 9. High-throughput screening of the possible products from solid-state reactions. All (upper panel) and top 20 (lower panel) possible products and corresponding reaction enthalpy change (ΔH) of the solid-state reactions between $BaCO_3$ + Al_2O_3 + Co_3O_4 + H_2 (Detailed data can be found in Source Data files). Source data are provided as a Source Data file.

Reaction enthalpy change (ΔH) of forming different products from the solid-state reactions between $BaCO_3$ and Al_2O_3, with Co_3O_4 and H_2 coexisting in the system, were theoretically examined by the reaction calculator module in Materials Project[58].

Before H_2 thermal treatment, a mix-metal precursor containing $BaCO_3$, Al_2O_3, and Co_3O_4 was obtained after calcination in the air. Therefore, we set the reactants as $BaCO_3$ + Al_2O_3 + Co_3O_4 + H_2 (because the solid-state reaction is under an H_2 atmosphere), while setting the possible products as all compounds containing Ba element and any one or more of Co, Al, C, O, and H. Searched from the Materials Project database, there are 53 possible different products. Then in the reaction calculator module, the reaction enthalpy change (ΔH) for obtaining each of the above 53 possible products was queried and sorted, as shown in Supplementary Fig. 9. The stability of the corresponding products is evaluated by comparing the value of ΔH. The lower the ΔH, the more stable the product. Supplementary Fig. 9 indicates the most stable three products are $Ba_7Al_{64}O_{103}$, $BaAl_4O_7$, and $BaAl_2O_4$. Given that the high reported synthesis temperatures of $BaAl_4O_7$ (c.a. 1100°C)[59] and $Ba_7Al_{64}O_{103}$ (higher than 1100°C, via solid-state reaction of $BaAl_4O_7$ and Al_2O_3)[60], we put our focus on $BaAl_2O_4$ with Ba/Al = 1/2, which has a milder synthesis condition[61] and thus more suitable for the one-pot synthesis of the target Co@$BaAl_2O_4$ encapsulation structure combined with the Tammann temperature guide.

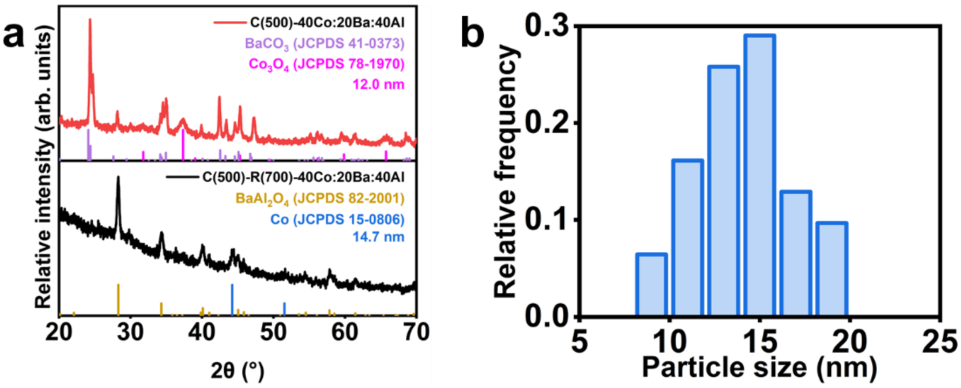

Supplementary Figure 10. Structure and configuration identification of Co@$BaAl_2O_4$ encapsulation structure. (a) Upper panels: XRD pattern of C(500)-40Co:20Ba:40Al. Lower panels: XRD pattern of C(500)-R(700)-40Co:20Ba:40Al. It can be observed that Co_3O_4 has been reduced to Co NPs during the H_2 thermal treatment, and $BaAl_2O_4$ is formed via the solid-state reaction between $BaCO_3$ and Al_2O_3. **(b)** Size distributions of Co NPs measured from low magnification TEM images of C(500)-R(700)-40Co:20Ba:40Al. Source data are provided as a Source Data file.

Supplementary Table 4
Estimation of Co NP crystallite sizes.

Sample	Phase (Peak selected for crystallite size calculation)	Co NP crystallite size from the Debye-Scherrer equation		Co NP size distribution from TEM, nm
		FWHM, °	Co NP crystallite size, nm	
C(500)-R(700)-40Co:20Ba:40Al	Co (111)	0.613	14.7	10 ~ 15

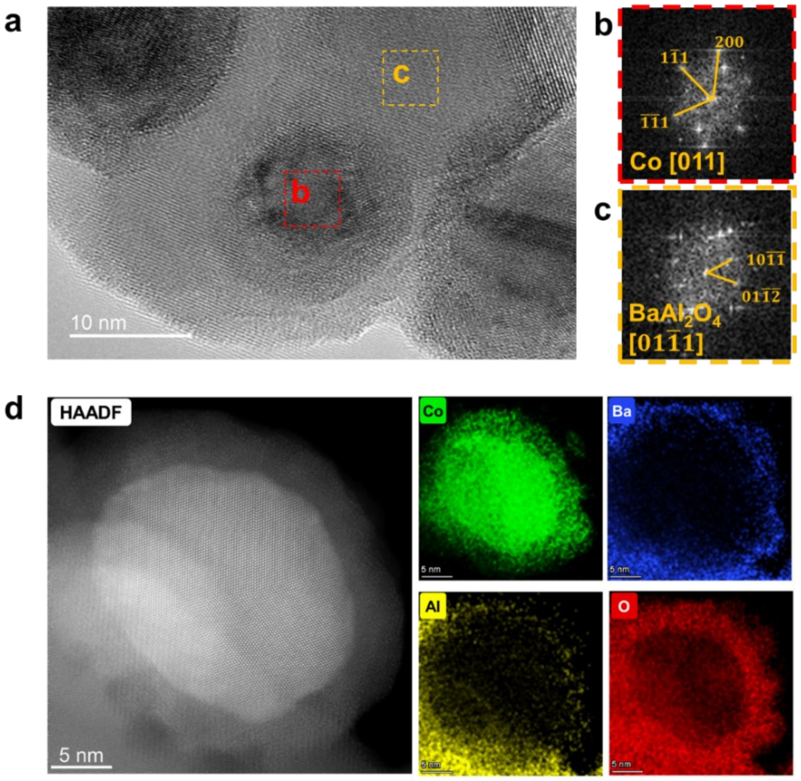

Supplementary Figure 11. Morphology and structural investigation of Co@BaAl$_2$O$_4$ from TEM. (a) High-resolution TEM image of Co@BaAl$_2$O$_4$. The red and yellow dashed boxes denote the regions in which the fast Fourier transform (FFT) was performed. **(b)** FFT pattern calculated from the red dashed box, which shows the fcc Co phase diffraction pattern along [011] zone axis. **(c)** FFT pattern calculated from the yellow dashed box, which shows the BaAl$_2$O$_4$ phase diffraction pattern along [01$\bar{1}$1] zone axis. **(d)** Atomic-scale STEM images and EDS element distribution maps (including Co, Ba, Al, and O elements) of Co@BaAl$_2$O$_4$.

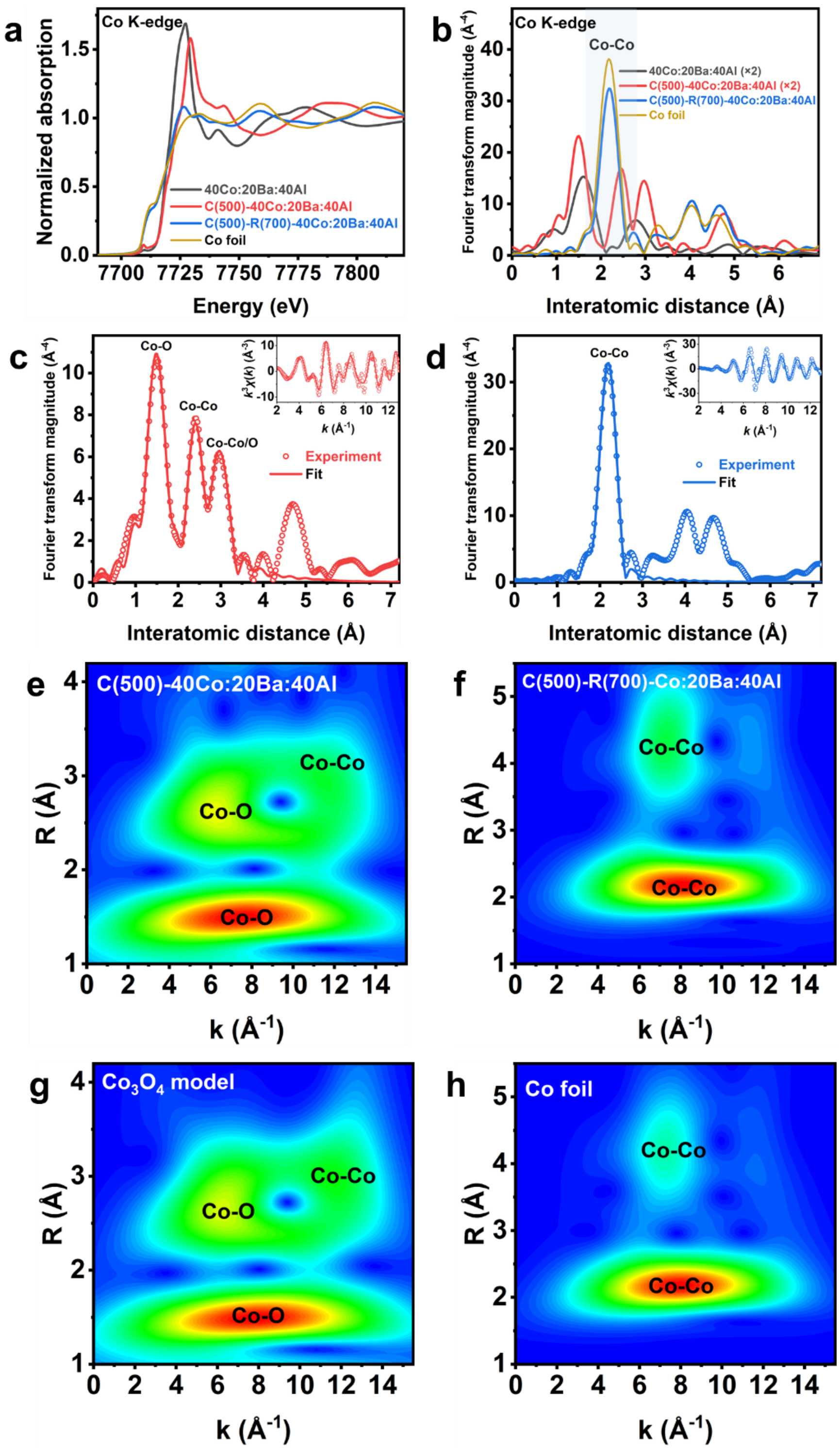

Supplementary Figure 12. Electronic properties and local structures of Co in Co@BaAl$_2$O$_4$. (a) Co K-edge XANES spectra of 40Co:20Ba:40Al, C(500)-40Co:20Ba:40Al, and C(500)-R(700)-40Co:20Ba:40Al, along with the Co foil for comparison purpose. **(b)** k^3-weighted Co K-edge Fourier transform (FT) EXAFS of 40Co:20Ba:40Al, C(500)-40Co:20Ba:40Al, and C(500)-R(700)-40Co:20Ba:40Al. **(c)** Co K-edge EXAFS fitting curves of C(500)-40Co:20Ba:40Al. **(d)** Co K-edge EXAFS fitting curves of C(500)-R(700)-40Co:20Ba:40Al. **(e-h)** WT representations of the Co K-edge EXAFS signals for: **(e)** C(500)-40Co:20Ba:40Al, **(f)** C(500)-R(700)-40Co:20Ba:40Al, **(g)** theoretical Co_3O_4 model, and **(h)** Co foil reference. Source data are provided as a Source Data file.

Supplementary Table 5

Co K-edge EXAFS fitting parameters of C(500)-40Co:20Ba:40Al and C(500)-R(700)-40Co:20Ba:40Al

Sample	Bond	CN	R, Å	D-W factor (σ^2), Å^2	ΔE_0, eV	R-factor, %
C(500)-40Co:20Ba:40Al	Co-O	4.3 (3)	1.91 (1)	0.003 (1)	-6.9	0.7
	Co-Co	2.0 (2)	2.87 (1)	0.003 (1)	-4.3	
	Co-Co	7.1 (5)	3.38 (1)	0.005 (2)		
	Co-O	2.4 (5)	3.50 (4)	0.007 (2)	-6.9	
	Co-Co	3.5 (4)	3.54 (1)	0.004 (1)	-4.3	
	Co-O	4.6 (3)	3.64 (2)	0.003 (2)	-6.9	
C(500)-R(700)-40Co:20Ba:40Al	Co-Co	8.4 (4)	2.48 (1)	0.006 (1)	7.8	1.9

*Co K-edge EXAFS fitting results, in which CN is the average coordination number, R is the distance from the absorber atom, and σ^2 the Debye–Waller factor. R-factor denotes the quality factor of the fitting, and ΔE_0 the energy shift from the absorption edge energy E_0.

Supplementary Table 6

Coordination environments of the absorbing Co atoms in theoretical Co_3O_4 and Co metal model

Theoretical structure	Bond	CN	R, Å
The average coordination environment of Co atoms in Co_3O_4	Co-O	5.3	1.92-1.93
	Co-Co	4.0	2.86
	Co-Co	8.0	3.35
	Co-O	4.0	3.39
	Co-Co	1.3	3.50
	Co-O	4.0	3.56
Co metal	Co-Co	12	2.50

Supplementary Fig. 12a shows the XANES features of the samples at different stages of the synthesis process (i.e., as-precipitated, as-calcined, and thermally treated in H_2). Before H_2 thermal treatment, the C(500)-40Co:20Ba:40Al shows a much higher white-line (WL) intensity compared to standard Co foil. After H_2 thermal treatment, the XANES profile for C(500)-R(700)-40Co:20Ba:40Al shifts to lower energy compared to C(500)-40Co:20Ba:40Al, becoming similar to the Co foil standard. These results indicate the formation of metallic Co from Co_3O_4 reduction during H_2 thermal treatment. Supplementary Fig. 12b shows the Fourier transforms (FT) magnitude of the Co K-edge EXAFS for the samples at different stages of the synthesis process, along with the Co foil for comparison purpose. The FT magnitude peak at 2.5 Å attributing to Co-Co only exists in C(500)-R(700)-40Co:20Ba:40Al while being absent in 40Co:20Ba:40Al and C(500)-40Co:20Ba:40Al. These results prove that Co NPs can only be formed after the H_2 thermal treatment. As shown in Supplementary Fig. 12c and Supplementary Table 5, the EXAFS spectrum of C(500)-40Co:20Ba:40Al can be well-fitted with three groups of paths, corresponding to Co-O (coordination numbers, CN = 4.3) at 1.91 Å, Co-Co (CN = 2.0) at 2.87 Å, and Co-Co/O at 3.38-3.64 Å, which agrees well the theoretical Co_3O_4 model (Supplementary Table 6). In addition, the EXAFS spectrum of C(500)-R(700)-40Co:20Ba:40Al can be well-fitted using only the Co-Co path, proving the sole existence of metallic Co for Co species after H_2 thermal treatment (Supplementary Fig. 12d and Supplementary Table 5).

The change of the local structures of the absorbing Co atoms can be observed more intuitively from wavelet transform (WT) representations of the Co K-edge EXAFS signals. It can be clearly observed that C(500)-40Co:20Ba:40Al has an entirely consistent spectrum with the theoretical Co_3O_4 model (Supplementary Fig. 12e and S12g), whereas C(500)-R(700)-40Co:20Ba:40Al has an entirely consistent spectrum with Co foil (Supplementary Fig. 12f-h), indicating the reduction of Co_3O_4 to Co metal during the H_2 thermal treatment. This result is consistent with the structural changes observed from the above XRD results (Supplementary Fig. 10a).

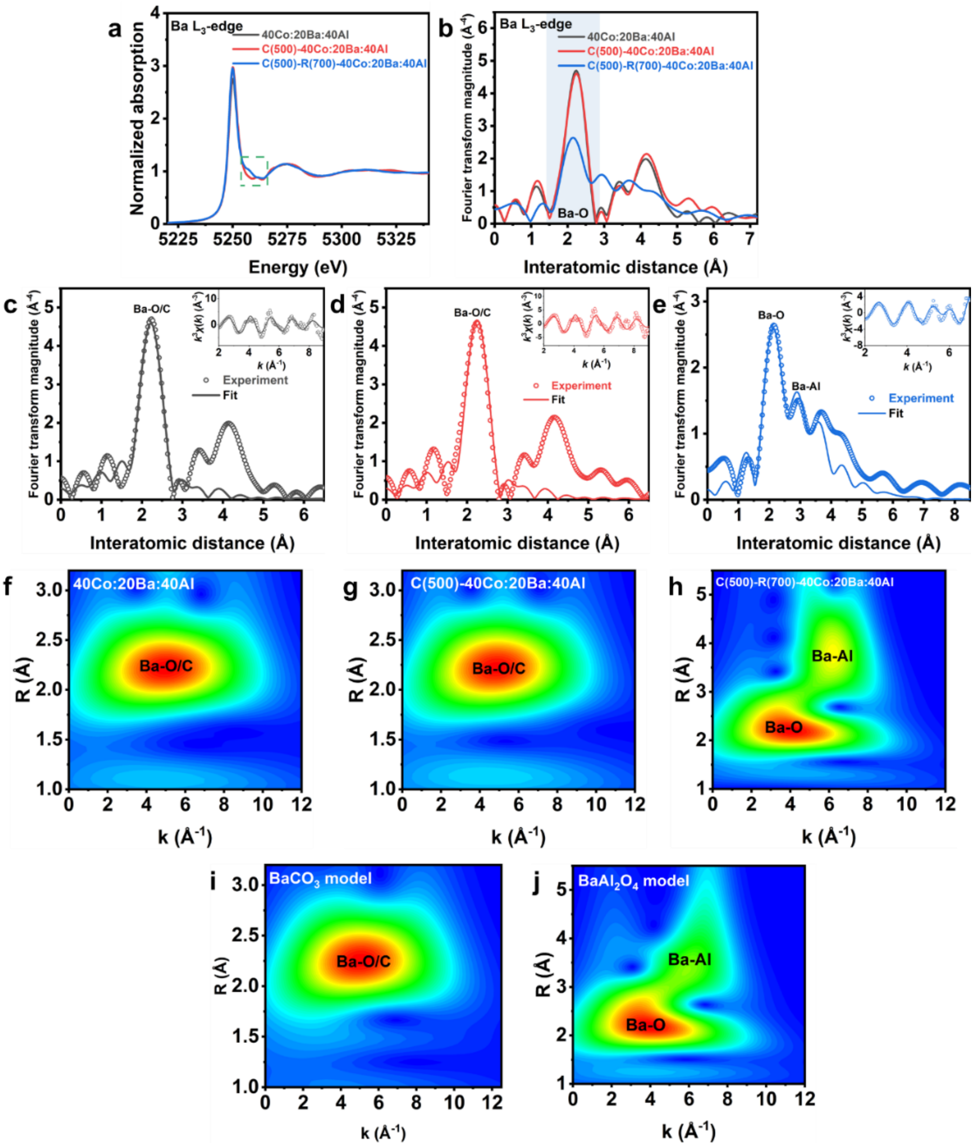

Supplementary Figure 13. Electronic properties and local structures of Ba in Co@BaAl$_2$O$_4$. (a) Ba L$_3$-edge XANES spectra of 40Co:20Ba:40Al, C(500)-40Co:20Ba:40Al, and C(500)-R(700)-40Co:20Ba:40Al. **(b)** k^3-weighted Ba L$_3$-edge Fourier transform (FT) EXAFS of 40Co:20Ba:40Al, C(500)-40Co:20Ba:40Al, and C(500)-R(700)-40Co:20Ba:40Al. **(c)** Ba L$_3$-edge EXAFS fitting curves of 40Co:20Ba:40Al. **(d)** Ba L$_3$-edge EXAFS fitting curves of C(500)-40Co:20Ba:40Al. **(e)** Ba L$_3$-edge EXAFS fitting curves of C(500)-R(700)-40Co:20Ba:40Al. **(f-j)** WT representations of the Ba L$_3$-edge EXAFS signals for: **(f)** 40Co:20Ba:40Al, **(g)** C(500)-40Co:20Ba:40Al, **(h)** C(500)-R(700)-40Co:20Ba:40Al, **(i)** theoretical $BaCO_3$ model, and **(j)** theoretical $BaAl_2O_4$ model. Source data are provided as a Source Data file.

Supplementary Table 7

Ba L$_3$-edge EXAFS fitting parameters of 40Co:20Ba:40Al, C(500)-40Co:20Ba:40Al, and C(500)-R(700)-40Co:20Ba:40Al.

Sample	Bond	CN	R, Å	D-W factor (σ^2), Å^2	ΔE$_0$, eV	R-factor, %
40Co:20Ba:40Al	Ba-O	4.4 (4)	2.73 (1)	0.003 (1)	7.7	0.8
	Ba-O	5.6 (5)	2.90 (1)	0.009 (2)		
	Ba-C	3.2 (4)	3.15 (3)	0.009 (2)	-9.3	

C(500)-40Co:20Ba:40Al	Ba-O	5.6 (4)	2.75 (1)	0.004 (2)	7.8	1.9
	Ba-O	5.6 (4)	2.90 (3)	0.008 (3)		
	Ba-C	3.7 (3)	3.16 (2)	0.009 (3)	-9.2	
C(500)-R(700)-40Co:20Ba:40Al	Ba-O	6.5 (4)	2.82 (2)	0.011 (1)	6.3	1.1
	Ba-O	1.2 (3)	3.09 (3)	0.012 (4)		
	Ba-Al	5.4 (4)	3.52 (1)	0.006 (1)	7.2	
	Ba-Al	5.9 (6)	3.90 (1)	0.013 (3)		

*Ba L_3-edge EXAFS fitting results, in which CN is the average coordination number, R is the distance from the absorber atom, and σ^2 the Debye–Waller factor. R-factor denotes the quality factor of the fitting, and ΔE_0 the energy shift from the absorption edge energy E_0.

Supplementary Table 8

Coordination environments of the absorbing Ba atoms in theoretical $BaCO_3$ and $BaAl_2O_4$ models.

Theoretical structure	Bond	CN	R, Å
$BaCO_3$	Ba-O	3.0	2.75
	Ba-O	6.0	2.80-2.90
	Ba-C	3.0	3.21
$BaAl_2O_4$	Ba-O	6.0	2.80
	Ba-O	3.0	3.01
	Ba-Al	6.0	3.48
	Ba-Al	6.0	3.99

Supplementary Fig. 13a shows the Ba L_3-edge XANES spectra of 40Co:20Ba:40Al, C(500)-40Co:20Ba:40Al, and C(500)-R(700)-40Co:20Ba:40Al. It can be observed that the edge positions remain unchanged between the three samples, indicating the Ba valence does not change much after air calcination and H_2 thermal treatment. In addition, the Ba L_3-edge XANES spectrum of C(500)-R(700)-40Co:20Ba:40Al shows a distinctive peak between 5250 and 5285 eV (Supplementary Fig. 13a), in line with the reported XANES features of $BaAl_2O_4$ structure. As Vrankić et al. reported[62], distinct differences between pure $BaAl_2O_4$ and pure $BaCO_3$ spectra are visible in the energy range between 5250 and 5270 eV, i.e., $BaCO_3$ has an absorption minimum at about 5255 eV and 5264 eV, where $BaAl_2O_4$ features a visible stronger intensity in this energy range. As can be seen in this comparison, the Ba L_3-edge XANES spectrum for 40Co:20Ba:40Al and C(500)-40Co:20Ba:40Al samples follow the spectrum of the pure $BaCO_3$ sample, while C(500)-R(700)-40Co:20Ba:40Al follows that of $BaAl_2O_4$. This observation is consistent with the phase transformation observed from the XRD results of the samples in different stages (Supplementary Fig. 10a).

From the Ba L_3-edge EXAFS spectrum at R space, it can be observed that the spectra of 40Co:20Ba:40Al and C(500)-40Co:20Ba:40Al are similar but much different from C(500)-R(700)-40Co:20Ba:40Al (Supplementary Fig. 13b). Ba L_3-edge EXAFS fitting was further conducted to identify the Ba local structures. The EXAFS spectra of 40Co:20Ba:40Al and C(500)-40Co:20Ba:40Al samples can be fitted well using the theoretical $BaCO_3$ model, with $\Delta R \leq 0.1$ nm and R-factor < 2% (Supplementary Fig. 13c-d, Supplementary Tables 7 and 8). And the EXAFS spectrum of C(500)-R(700)-40Co:20Ba:40Al can be well-fitted using the theoretical $BaAl_2O_4$ model (Supplementary Fig. 13e, Supplementary Tables 7 and 8).

The structure change can be observed more intuitively from Ba L_3-edge WT EXAFS. It can be clearly seen that 40Co:20Ba:40Al and C(500)-40Co:20Ba:40Al samples have entirely consistent spectra with the theoretical $BaCO_3$ model (Supplementary Figs. 13f, 13g, and 13i). In contrast, the sample C(500)-R(700)-40Co:20Ba:40Al has a consistent spectrum with the theoretical $BaAl_2O_4$ model (Supplementary Fig. 13h and 13j), indicating the phase evolution from $BaCO_3$ to $BaAl_2O_4$ during H_2 thermal treatment. This result is consistent with the structural changes observed from the above XRD results (Supplementary Fig. 10a).

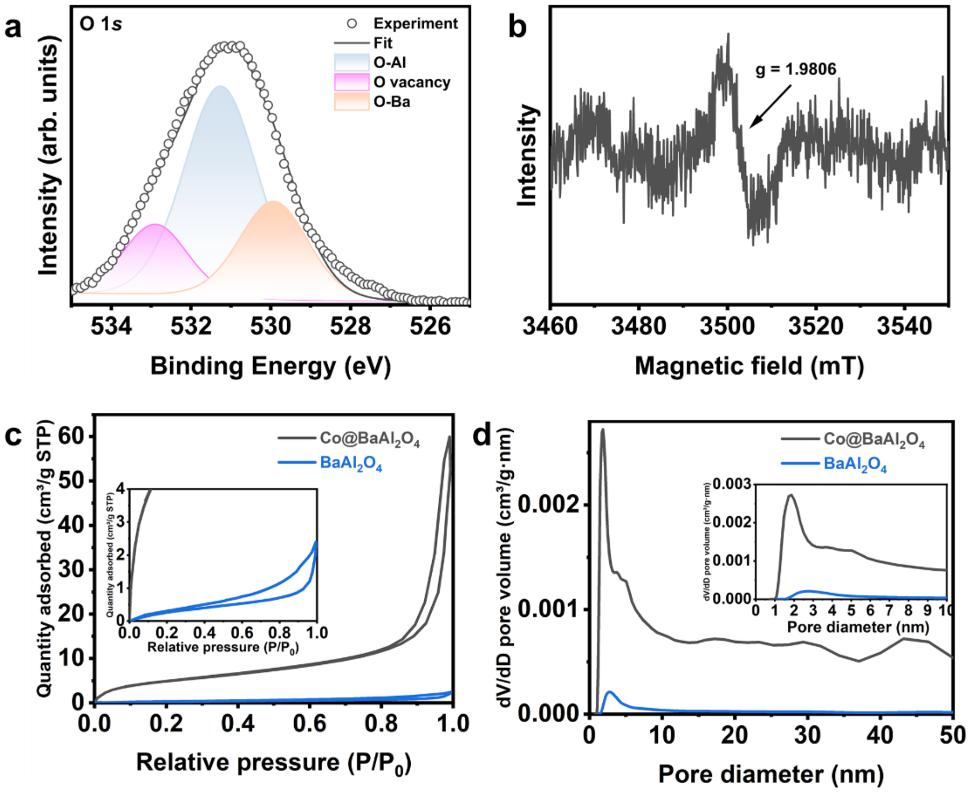

Supplementary Figure 14. Oxygen vacancies in the $BaAl_2O_4$ overlayer of Co@$BaAl_2O_4$. **(a)** XPS spectra of the O1*s* core level for Co@$BaAl_2O_4$, which shows a peak at binding energies of 532.2 eV attributing to the oxygen vacancies in the $BaAl_2O_4$ lattice[63]. **(b)** EPR spectra of $BaAl_2O_4$, which shows a signal with the splitting factor values (g) of 1.9806, corresponding to oxygen vacancies with a single trapped electron (V_O^+). **(c)** Ar adsorption-desorption isotherms of Co@$BaAl_2O_4$ and pristine $BaAl_2O_4$. **(d)** The density functional theory (DFT) pore size distribution curves of Co@$BaAl_2O_4$ and pristine $BaAl_2O_4$. Source data are provided as a Source Data file.

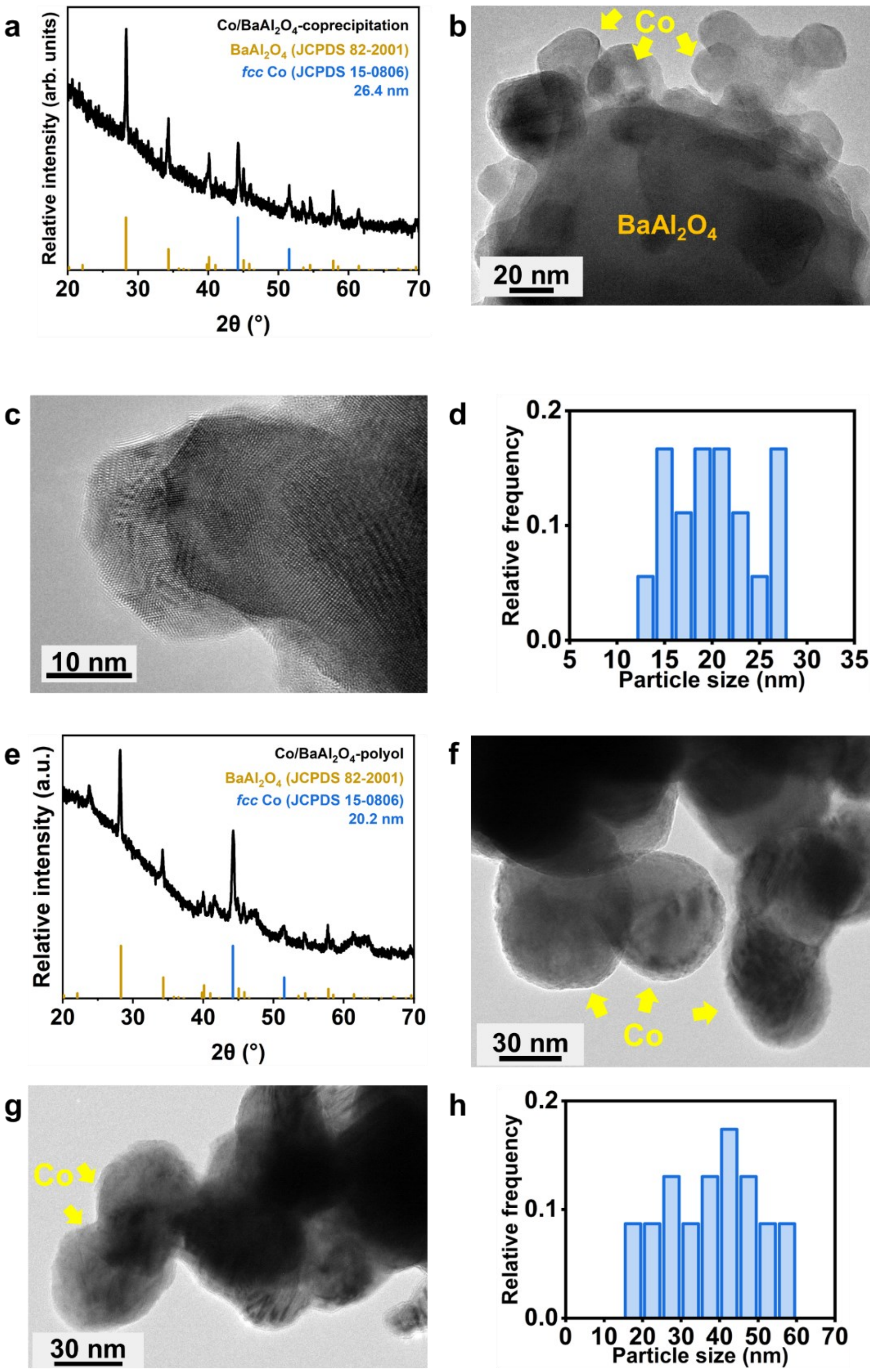

Supplementary Figure 15. Necessity of low-T_{Tam} $M_{AE}CO_3$ as the facilitator. (a-d) Structure and configuration identification of $Co/BaAl_2O_4$-coprecipitation: **(a)** XRD pattern of $Co/BaAl_2O_4$-coprecipitation, **(b-c)** TEM images of $Co/BaAl_2O_4$-coprecipitation, and **(d)** Co NP size distribution of $Co/BaAl_2O_4$-coprecipitation measured from low magnification TEM images. **(e-h)** Structure and configuration identification of $Co/BaAl_2O_4$-polyol: **(e)** XRD pattern of $Co/BaAl_2O_4$-polyol, **(f-g)** TEM images of $Co/BaAl_2O_4$-polyol, and **(h)** Co NP size distribution of $Co/BaAl_2O_4$-polyol measured from low magnification TEM images. Source data are provided as a Source Data file.

Supplementary Table 9

Estimation of Co NP crystallite sizes in $Co/BaAl_2O_4$-coprecipitation and $Co/BaAl_2O_4$-polyol.

Sample	Phase (Peak selected for crystallite size calculation)	Co NP crystallite size from the Debye-Scherrer equation		Co NP size distribution from TEM, nm
		FWHM, °	Co NP crystallite size, nm	
$Co/BaAl_2O_4$-coprecipitation	Co (111)	0.325	26.4	15 ~ 28 nm
$Co/BaAl_2O_4$-polyol	Co (111)	0.424	20.2	18 ~ 60 nm

It was reported in the literature that $BaAl_2O_4$ can be directly synthesised by calcinating $BaCO_3$ and Al_2O_3 in the air over 900 °C[64]; therefore, we directly calcined the mixture precursor 40Co:20Ba:40Al at 900 °C in the air to directly form $BaAl_2O_4$, followed by H_2 thermal treatment at 700 °C to reduce Co_3O_4 to Co NPs, and the obtained product was denoted as $Co/BaAl_2O_4$-coprecipitation. As shown in Supplementary Fig. 15a, Co and $BaAl_2O_4$ phases can be observed after H_2 thermal treatment, but cannot form an encapsulation configuration (Supplementary Fig. 15b-c). It should be noted that, in this synthesis process, the low-T_{Tam} $BaCO_3$ has been completely converted to $BaAl_2O_4$ upon high-temperature air calcination (900 °C), which cannot have enough mobility to encapsulate the in-situ formed Co NPs, therefore cannot form encapsulation structure. This result demonstrates the critical role of mobile $BaCO_3$ in forming the Co@$BaCO_3$ encapsulation structure.

In addition, we prepared $BaAl_2O_4$ by calcinating $Ba(NO_3)_2$ and Al_2O_3 in the air[61], and then incorporated Co NPs onto $BaAl_2O_4$ via the polyol method[65]. The obtained sample was then thermal treated under H_2 at 700 °C to investigate if the encapsulation can be generated on $BaAl_2O_4$-supported Co NPs upon thermal treatment in H_2. The H_2 thermal treated sample was donated as $Co/BaAl_2O_4$-polyol, in which Co and $BaAl_2O_4$ phases can be observed from XRD (Supplementary Fig. 15e) while no encapsulation structure is observed from the TEM images (Supplementary Fig. 15f-g). This observation proves that the conventional SMSI cannot be generated by reducing the $BaAl_2O_4$ support under H_2 even at a high temperature at 700 °C.

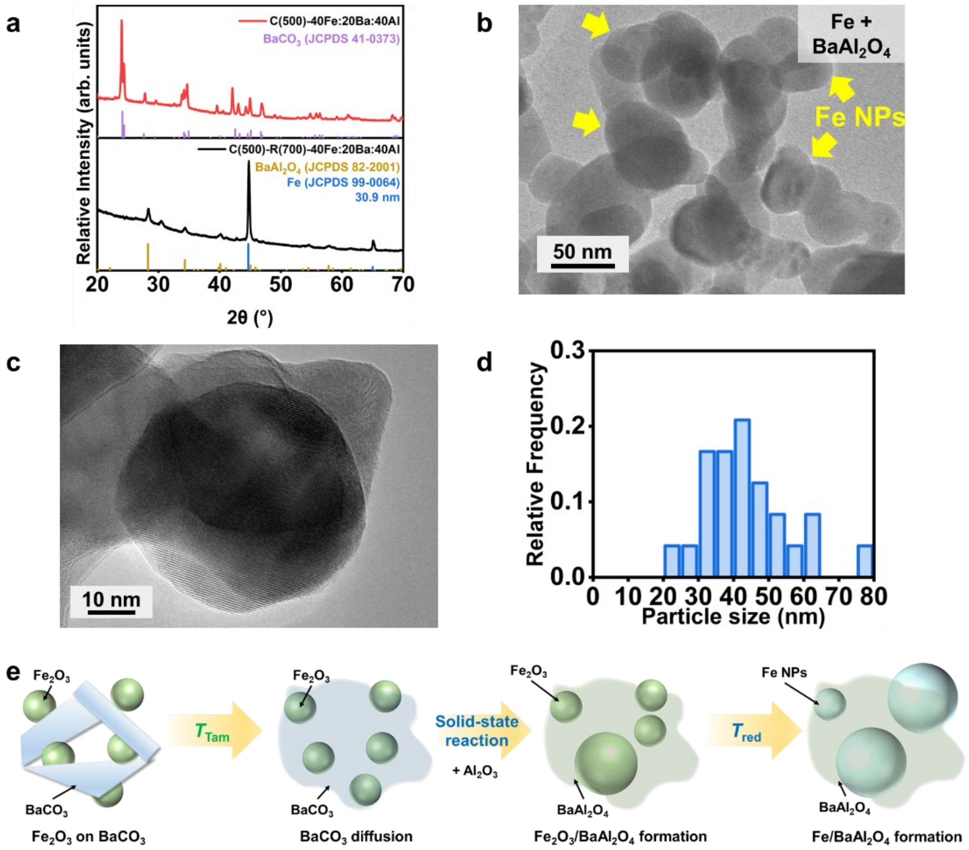

Supplementary Figure 16. Control sample which falls either outside or on the edge of the encapsulable window: Fe_2O_3 + $BaCO_3$ + Al_2O_3. (a) XRD patterns of C(500)-40Fe:20Ba:40Al precursor and final obtained C(500)-R(700)-40Fe:20Ba:40Al. **(b-c)** TEM images of final obtained C(500)-R(700)-40Fe:20Ba:40Al. **(d)** Fe NP size distribution measured from low-magnitude TEM images. **(e)** Schematic illustration of the reasons for the failure of the Fe@$BaAl_2O_4$ core@shell structure synthesis from Fe_2O_3 + $BaCO_3$ + Al_2O_3 mixture precursors. Source data are provided as a Source Data file.

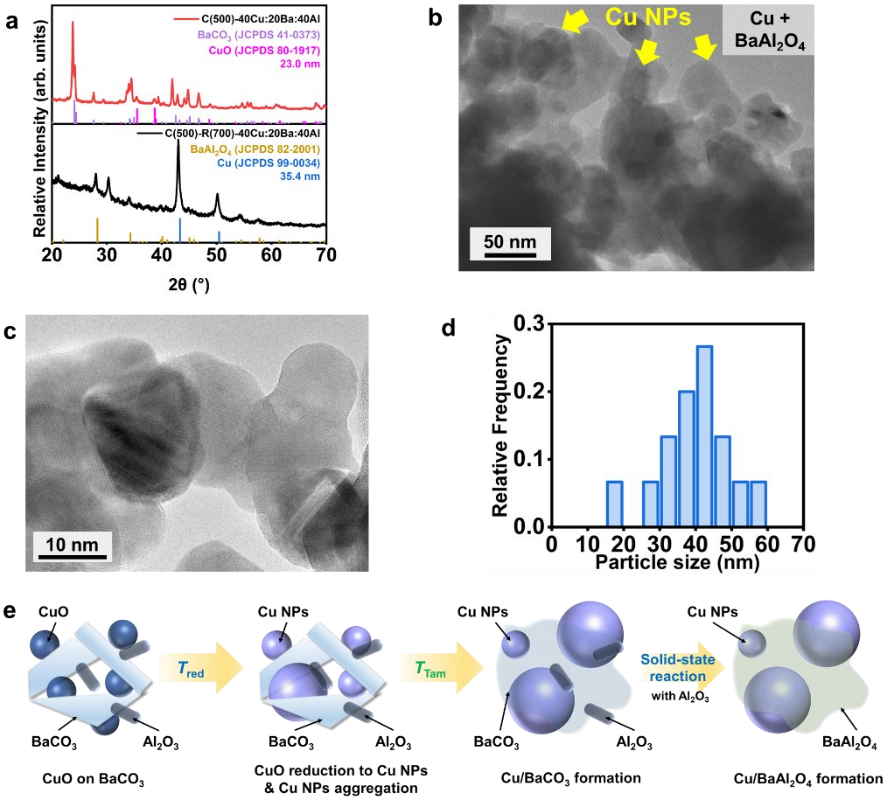

Supplementary Figure 17. Control sample which falls either outside or on the edge of the encapsulable window: CuO + $BaCO_3$ + Al_2O_3. (a) XRD patterns of C(500)-40Cu:20Ba:40Al precursor and final obtained C(500)-R(700)-40Cu:20Ba:40Al. **(b-c)** TEM images of final obtained C(500)-R(700)-40Cu:20Ba:40Al. **(d)** Cu NP size distribution measured from low-magnitude TEM images. **(e)** Schematic illustration of the reasons for the failure of the Cu@$BaAl_2O_4$ core@shell structure synthesis from CuO + $BaCO_3$ + Al_2O_3 mixture precursors. Source data are provided as a Source Data file.

To validate the synthesis rationale, we conducted experiments involving M_TO_x-$M_{AE}CO_3$ combinations where the $T_{red}(M_TO_x)$ falls either outside or on the edge of the encapsulation window. Specifically, we examined the Fe_2O_3-$BaCO_3$ and CuO-$BaCO_3$ combinations. In both cases, after thermally treating mixtures of 40Fe:20Ba:40Al and 40Cu:20Ba:40Al in a H_2 atmosphere, we observed no encapsulation, and the resulting Fe and Cu NPs displayed displayed severe coalescence and broad size distributions (Supplementary Figs. 16a-d and 17a-d).

For the Fe_2O_3 + $BaCO_3$, it's important to note that $BaCO_3$ decomposes into high-T_{Tam} BaO before Fe NPs can form from Fe_2O_3. Consequently, Fe NPs undergo substantial sintering without the protective encapsulation effect provided by $BaCO_3$ (Supplementary Fig. 16). In the case of CuO + $BaCO_3$, on the other hand, when the temperature reached the T_{red} of CuO (~250 °C), which is still lower than the T_{Tam} of $BaCO_3$ (269 °C), the limited mobility of $BaCO_3$ is insufficient to form an encapsulation layer on Cu NPs. This also results in significant sintering of Cu NPs (Supplementary Fig. 17).

These control experiments serve as valuable cross-check examples, affirming the validity of our synthesis rationale for the new thermal encapsulation strategy guided by Tammann temperature.

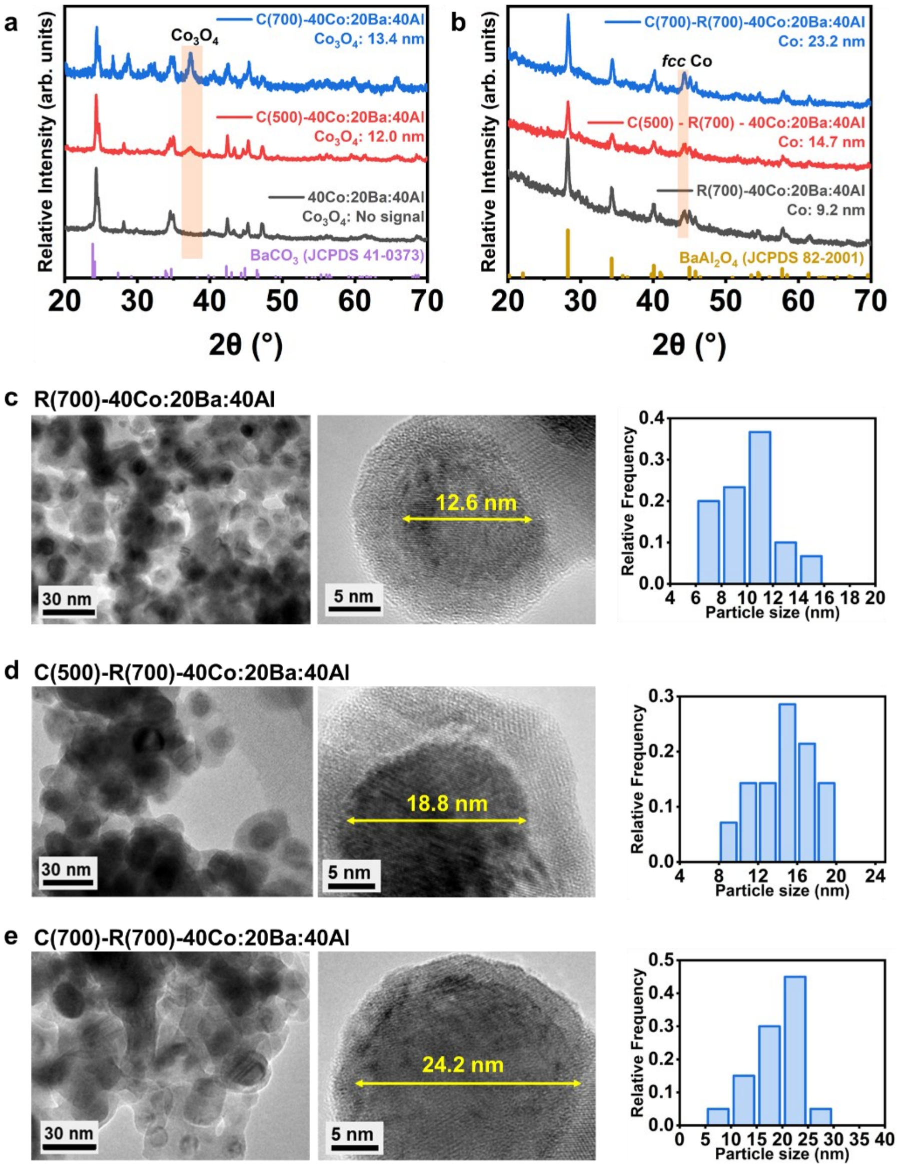

Supplementary Figure 18. Effect of Co_3O_4 precursor particle sizes on SMSI formation. **(a)** XRD patterns of Co_3O_4 + $BaCO_3$ + Al_2O_3 mixture precursors with different air-calcination temperatures. **(b)** XRD patterns of final obtained Co@$BaAl_2O_4$ core@shell structure with different air-calcination temperatures. **(c-e)** TEM images and Co NPs size distribution measured from low-magnitude TEM images of final obtained Co@$BaAl_2O_4$ core@shell structure with different air-calcination temperatures: **(c)** without calcination, **(d)** 500°C calcined, and **(e)** 700°C calcined. Source data are provided as a Source Data file.

To control the particle sizes of the precursor mixtures, we introduced an air calcination step before the H_2 thermal treatment. By adjusting this air calcination temperature, we synthesized mixture precursors with different Co_3O_4 sizes, and subjected them to subsequent H_2 thermal treatment. Supplementary Fig. 18a shows the XRD patterns of the mixture precursors calcined in air at different temperatures, i.e., C(temp.)-40Co:20Ba:40Al, and the crystallite sizes of Co_3O_4 calculated from the Debye-Scherrer equation. It can be observed that mixture precursors containing Co_3O_4 and $BaCO_3$ have been successfully synthesized after air calcination, and the crystallite sizes of Co_3O_4 increase as air calcination temperature increases from room temperature to 700 °C.

After H_2 thermal treatment at 700 °C, the corresponding products with encapsulation structures were obtained, donated as C(temp.)-R(700)-40Co:20Ba:40Al. C(temp.) and R(700) indicate the air-calcination and H_2 treatment processes, and the highest temperature (°C) reached in each process is listed in the brackets (700°C for H_2 treatment processes). Notably, as shown in Supplementary Fig. 18b-d and Supplementary Table 10, the Co NPs crystallite sizes also increase as air calcination temperature increases, from 6 ~ 12 nm in R(700)-40Co:20Ba:40Al to 15 ~ 25 nm in C(700)-R(700)-40Co:20Ba:40Al. This result indicates that air calcination temperatures can be regulated to adjust the size of Co_3O_4 precursors, to further alter the Co NP sizes in the final obtained core@shell structures.

Supplementary Table 10

Estimation of Co_3O_4 crystallite sizes in C(temp.)-40Co:20Ba:40Al and the associated Co NPs crystallite sizes in C(temp.)-R(700)-40Co:20Ba:40Al.

Sample	Phase (Peak selected for crystallite size calculation)	Crystallite size calculated using the Debye-Scherrer equation		Co NPs size distribution from TEM, nm	$BaAl_2O_4$ overlayer thickness from TEM, nm
		FWHM, °	Crystallite size, nm		
40Co:20Ba:40Al	No obvious Co_3O_4 (311) peak	/	/	/	/
C(500)-40Co:20Ba:40Al	Co_3O_4 (311)	0.702	12.0	/	/
C(700)-40Co:20Ba:40Al	Co_3O_4 (311)	0.628	13.4	/	/
R(700)-40Co:20Ba:40Al	Co (111)	0.927	9.2	6 ~ 12	c.a.4.0
C(500)-R(700)-40Co:20Ba:40Al	Co (111)	0.613	14.7	10 ~ 20	c.a. 4.1
C(700)-R(700)-40Co:20Ba:40Al	Co (111)	0.371	23.2	15 ~ 25	c.a. 3.5

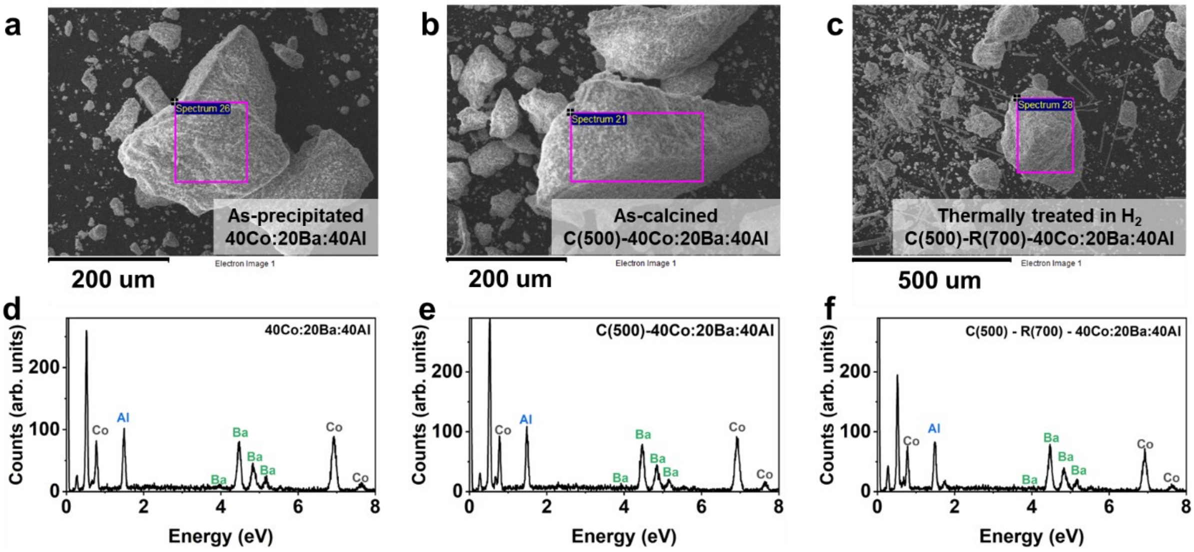

Supplementary Figure 19. Elemental composition analyses during the synthesis process. **(a)** SEM image of 40Co:20Ba:40Al, **(b)** SEM image of C(500)-40Co:20Ba:40Al, and **(c)** SEM image of C(500)-R(700)-40Co:20Ba:40Al. **(d)** EDS chemical composition analyses of 40Co:20Ba:40Al, **(e)** EDS chemical composition analyses of C(500)-40Co:20Ba:40Al, and **(f)** EDS chemical composition analyses of C(500)-R(700)-40Co:20Ba:40Al. It can be observed that the atomic ratio of Co, Ba, and Al remains unchanged at about 2:1:2 in each step during the whole synthesis process without significant loss, which is consistent with the original precursor metal composition. Source data are provided as a Source Data file.

Supplementary Table 11

The atomic ratio of Co, Ba, and Al elements in 40Co:20Ba:40Al, C(500)-40Co:20Ba:40Al, and C(500)-R(700)-40Co:20Ba:40Al measured by SEM-EDS.

Samples	Nominal atomic ratio			Atomic ratio by SEM-EDS		
	Co	Ba	Al	Co	Ba	Al
40Co:20Ba:40Al	40.00%	20.00%	40.00%	46.13%	19.85%	34.02%
C(500)-40Co:20Ba:40Al				46.17%	18.75%	35.08%
C(500)-R(700)-40Co:20Ba:40Al				40.93%	22.18%	36.89%

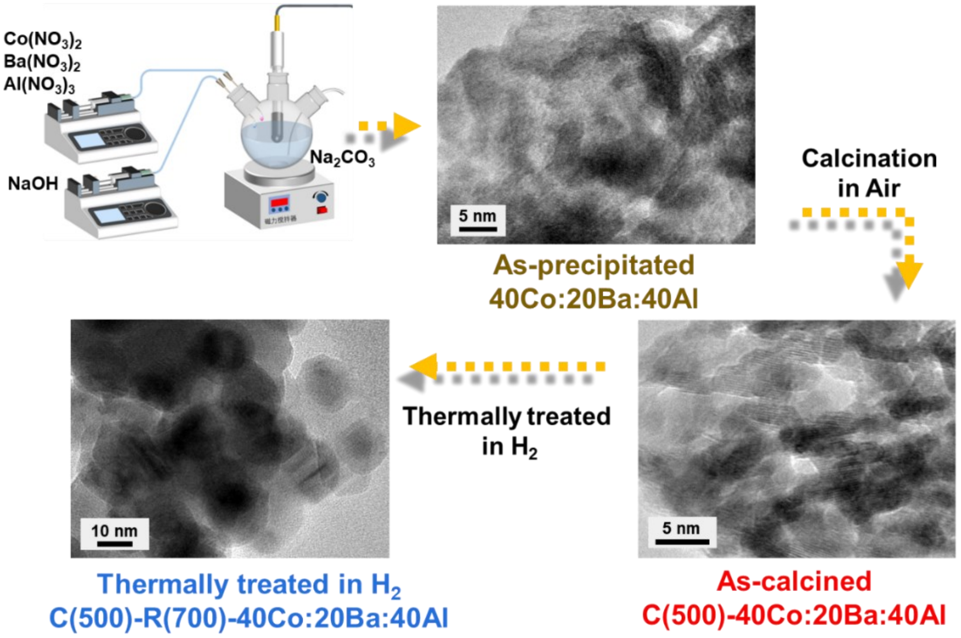

Supplementary Figure 20. Morphology evolution during the synthesis process. TEM images of the samples at different stages of the synthesis process (i.e., as-precipitated, as-calcined, and thermally treated in H_2).

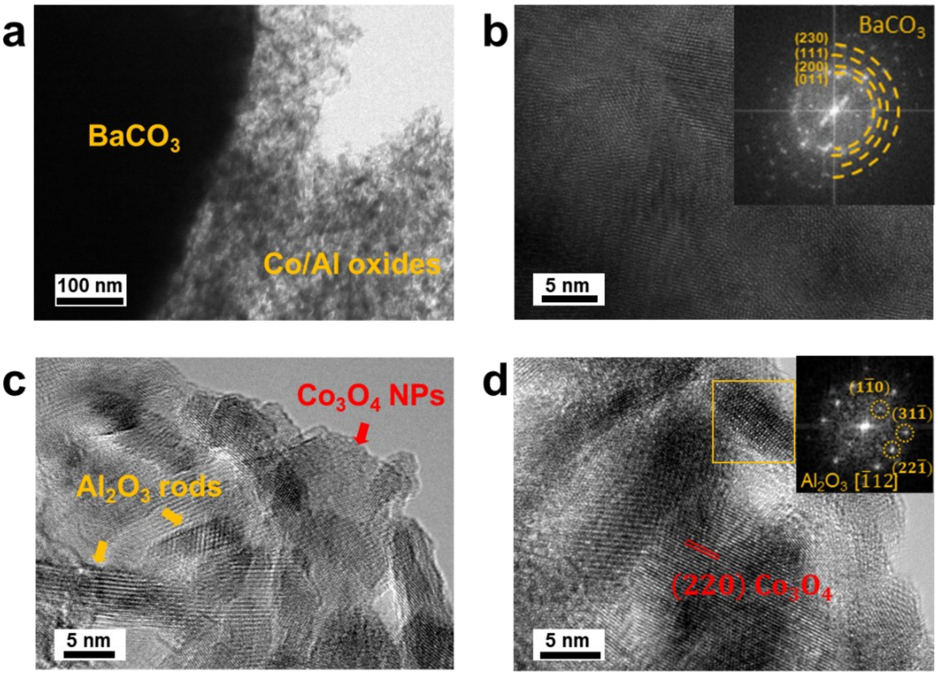

Supplementary Figure 21. Morphology of mix-metal precursor before H_2 thermal treatment. (a) Low magnification TEM image of the precursor C(500)-40Co:20Ba:40Al. **(b)** TEM image of $BaCO_3$ region. Inset is the corresponding FFT pattern of the whole area, which corresponds to the (230), (111), (220), and (011) planes of $BaCO_3$ phase. **(c-d)** High-resolution TEM images of Co/Al oxides region. Inset of (d) is the FFT pattern calculated from marked Al_2O_3 rods, which shows the Al_2O_3 phase diffraction pattern along [$\bar{1}$12] zone axis.

To better grasp the morphology evolution during the H_2 thermal treatment, starting morphology of the mix-metal precursor C(500)-40Co:20Ba:40Al before H_2 thermal treatment was characterised by TEM. As shown in Supplementary Fig. 21a, before H_2 thermal treatment, C(500)-40Co:20Ba:40Al is initially a mixture of plate-like Co_3O_4 and rod-like Al_2O_3 NPs, in contact with much larger bulk $BaCO_3$. A clear boundary is observed between the Co/Al-containing and Ba-containing areas. High-resolution TEM image and corresponding FFT shown in Supplementary Fig. 21b reveal the polycrystalline nature of $BaCO_3$ structure, consistent with the abovementioned XRD results (Supplementary Fig. 10a). The high-resolution TEM image shown in Supplementary Fig. 21c demonstrates that Al_2O_3 is in rod-like morphology and Co_3O_4 is in plate-like morphology, whose (220) lattice fringes are marked in Supplementary Fig. 21d.

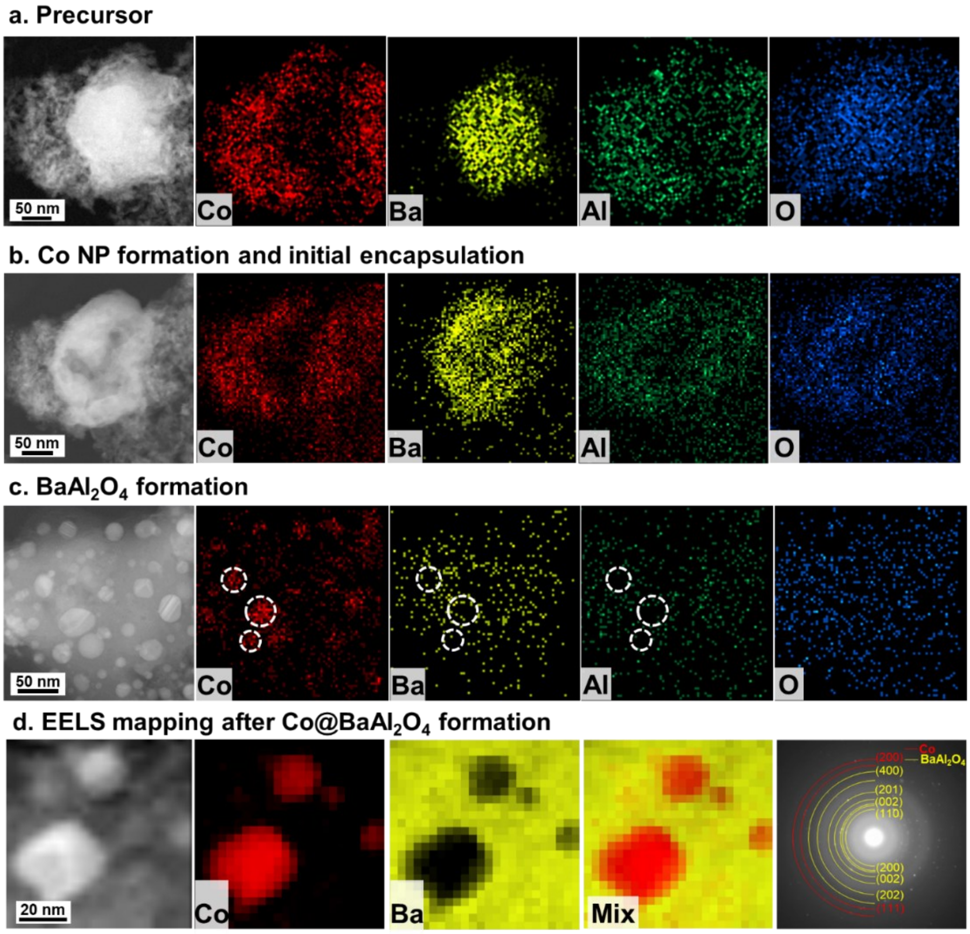

Supplementary Figure 22. Real-time observation of the element distribution evolution by in-situ STEM-EDS. EDS mapping in the same area of **(a)** the as-calcined precursor C(500)-40Co:20Ba:40Al, **(b)** the specimen after 500 °C in-situ reduction for 30 min, and **(c)** the specimen after 800 °C in-situ reduction for 30 min. **(d)** EELS mapping and corresponding selected area electron diffraction (SAED) pattern of the specimen after 800 °C in-situ reduction for 30 min.

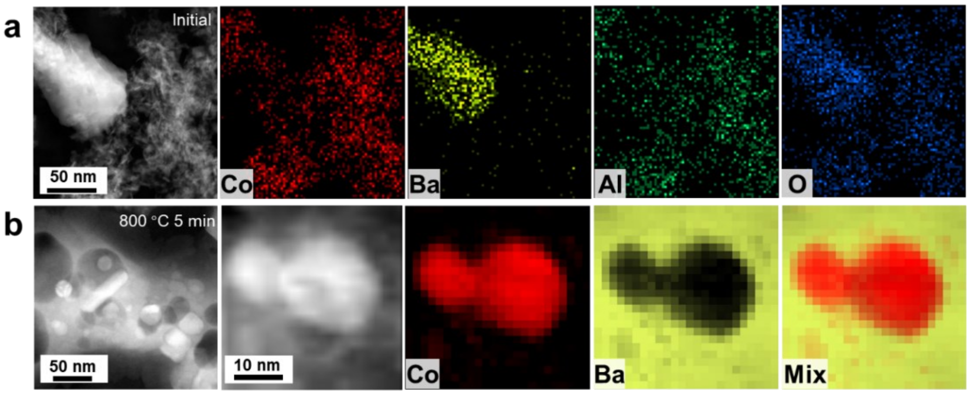

Supplementary Figure 23. Evaluation of the electron beam influence on the observed sample. (a) EDS mapping of the mixed metal precursor outside the in-situ tracked region. **(b)** EELS mapping of the specimen outside the in-situ tracked region after 800 °C reduction for 30 min.

The effect of the electron beam was carefully evaluated by comparing the sample state in the observation region with that out of the observation region. As shown in Supplementary Fig. 23a and S23b, Ba element diffusion and final encapsulation structure can be significantly observed, which are almost the same as our observation region, indicating that the influence of the electron beam on the observation region can be ignored.

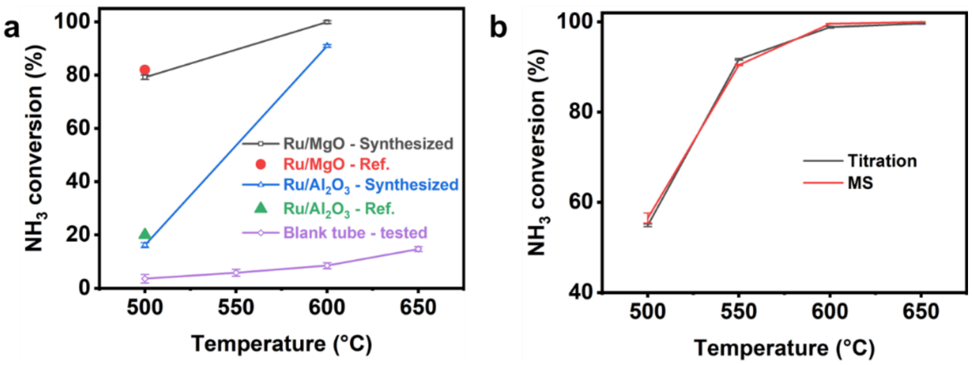

Supplementary Figure 24. The reliability of NH_3 decomposition performance testing equipment and the accuracy of product composition analyses. (a) NH_3 conversions comparison between reported benchmark catalysts and our verification experiment results, with error bars showing standard errors. **(b)** NH_3 conversions obtained from MS analyses and titration analyses, with error bars showing standard errors. Source data are provided as a Source Data file.

To verify the reliability of the performance testing equipment, two benchmark catalysts were synthesised following the reported methods, those are Ru/MgO by polyol method[66] and Ru/Al_2O_3 by co-precipitation method[67]. Their NH_3 decomposition performances were tested using our equipment at 500 to 650 °C and WHSV of 30,000 mL g_{cat}^{-1} h^{-1}. The obtained result was compared to the reported values. As shown in Supplementary Fig. 24a, our measured NH_3 conversions are almost the same as those reported in the literature, indicating that our performance testing equipment is reliable. In addition, to guarantee the accuracy of the reaction product analyses method, both MS analyses and back titration experiment were applied to analyse the NH_3 content in the outlet gas (Supplementary Fig. 24b). The experimental details of back titration analyses have been reported in our previous studies[2]. It can be observed that the results of the two analytical methods are consistent, only with a tiny difference of less than 3% in NH_3 conversion, hence confirming the accuracy of our analyses method for reaction product compositions.

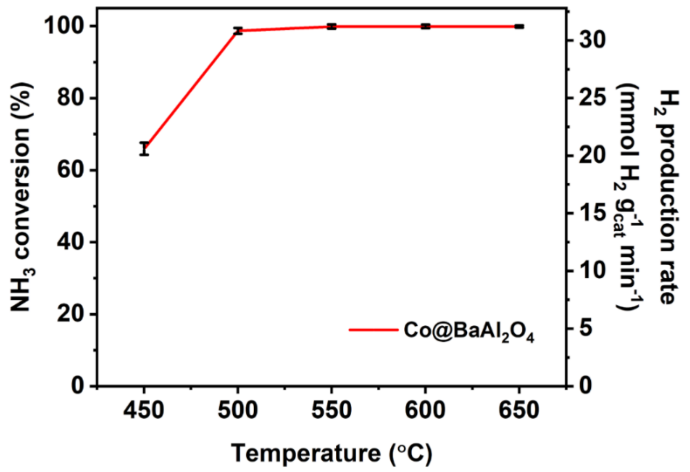

Supplementary Figure 25. Catalytic performances on NH_3 decomposition of $Co@BaAl_2O_4$ catalysts. NH_3 decomposition conversion and H_2 production rate over $Co@BaAl_2O_4$ catalysts as a function of reaction temperature. The performance was evaluated at weight hourly space velocity (WHSV) of 30,000 mL g_{cat}^{-1} h^{-1} and 1 atm, with error bars showing standard errors. Source data are provided as a Source Data file.

Supplementary Table 12. Catalytic performances on NH_3 decomposition of $Co@BaAl_2O_4$ and other reported core@shell catalysts at 1 atm.

Catalysts	WHSV, mL g_{cat}^{-1} h^{-1}	Temperature, °C	NH_3 conversion, %	H_2 production rate, mmol H_2 g_{cat}^{-1} min^{-1}	Year	Ref.
$Co@BaAl_2O_4$	30,000	450	66.0	20.6	This work	
		500	98.7	30.9		
		550	99.8	31.2		
		600	99.9	31.2		
		650	99.9	31.2		
$Ru@SiO_2$	30,000	500	90	28.1	2010	68
$LaCoO_x/Co@NC/SBA-15$	30,000	500	90	28.1	2023	69
$Ru@SiO_2$	30,000	500	82	25.6	2010	70
$Mo_2N/SBA-15/Rgo$	30,000	500	65	20.3	2021	71
$Co_{0.89}Fe_{2.11}O_4@mSiO_2$	22,800	500	70	16.6	2019	72
Ru/Rb-Y	30,000	500	50	15.6	2021	73
Ru/K-Y	30,000	500	45	14.1	2021	73
Ru@MIL-101	15,000	500	85	13.3	2018	74
Carbon embedded Fe	30,000	500	40	12.5	2020	75
$Ni@Al_2O_3$	24,000	500	50	12.5	2021	76

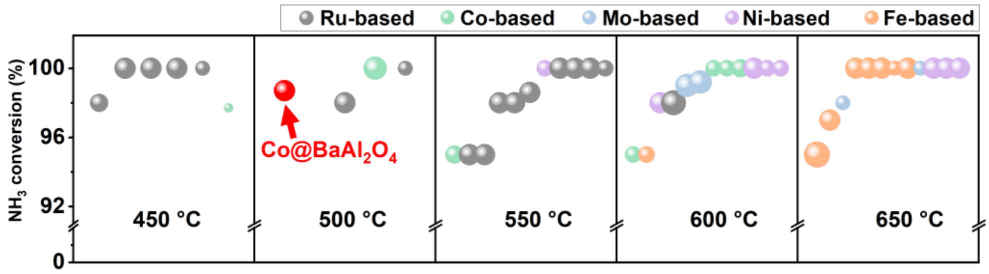

Supplementary Figure 26. Co@BaAl$_2$O$_4$ represents one of the most effective catalysts in both non-noble and noble catalysts records. Comparison of NH$_3$ conversion between the Co@BaAl$_2$O$_4$ (red balls) and the reported Ru-based and non-noble metal-based catalysts. The sizes of the balls represent the value of WHSV. The larger the ball diameter, the higher the WHSV. Detailed data can be found in Supplementary Table 13. Source data are provided as a Source Data file.

Supplementary Table 13

Selected catalysts from literature with high NH$_3$ decomposition performances (NH$_3$ conversion ≥ 95%) at 1 atm and high WHSV (> 15,000 mL g_{cat}^{-1} h^{-1}).

Catalysts	wt.%	WHSV (mL g_{cat}^{-1} h^{-1})	Temperature (°C)	NH$_3$ conversion (%)	Year	References
Ru/Al$_2$O$_3$	5.7	30,000	550	98	2007	67
Ru/Mg-Al$_2$O$_3$		30,000	550	98.6	2007	77
Ru/GC	5.0	30,000	550	95	2007	78
Ru@La$_2$O$_3$-ZrO$_2$		30,000	550	~ 100	2010	79
Ru@SiO$_2$		30,000	550	~ 100	2010	70
Ru/Pr$_6$O$_{11}$	5.0	30,000	450	~ 100	2010	80
Ru/La$_2$O$_3$	5.0	30,000	450	~ 100	2010	80
Ru/CNT		42,000	600	98	2010	81
Ru/C$_{12}$A$_7$:e$^-$	2.2	15,000	450	~ 100	2013	82
Ru/C$_{12}$A$_7$:O^{2-}	2.0	15,000	500	~ 100	2013	82
Ru/Al$_2$O$_3$	4.6	30,000	550	95	2018	83
Ru/K-Mg$_2$Al-LDO	4.6	30,000	550	~ 100	2018	83
Ru/La$_2$O$_3$	4.8	18,000	550	~ 100	2019	84
Ru/BaAl$_{12}$O$_{19}$	2.7	30,000	500	98	2019	85
Ru/CeO$_2$	1.0	22,000	450	98	2020	86
Ru/SiO$_2$-GUS	5.0	30,000	550	98	2021	87
Ru/MgO(111)		30,000	450	100	2022	88
Fe@micro-SiO$_2$		30,000	650	97	2010	89
Fe@meso-SiO$_2$		30,000	650	~ 100	2010	89
Fe@meso-SiO$_2$-Cs		30,000	650	~ 100	2010	89
Fe@SiO$_2$		30,000	650	~ 100	2010	68
Fe/FeN$_x$@pSiO$_2$	42	15,000	650	~ 100	2010	90
Fe$_5$Mo$_5$/C-ZrO$_2$	5	46,000	650	95	2012	91
FeLaO$_3$		18,000	600	95	2017	92
Fe/ZSM-5	5	30,000	650	~ 100	2018	93
CoLaO$_3$		18,000	600	95	2017	92
Co/Al$_2$O$_3$	90	18,000	600	~ 100	2018	94
(Co,Al)(Co,Al)$_2$O$_4$	50	18,000	600	~ 100	2015	95
Co/La-MgO	20	22,000	600	~ 100	2019	96
Co/MgO	20	22,000	550	95	2019	96
Co$_{55}$Mo$_{15}$Fe$_{10}$Ni$_{10}$Cu$_{10}$		36,000	500	~ 100	2019	3
Nano-Ni@SiO$_2$		30,000	650	~ 100	2011	97
Nano-Ni+La@SiO$_2$		30,000	600	98	2011	97
Nano-Ni+Ce@SiO$_2$		30,000	600	~ 100	2011	97
Ni$_{0.5}$Ce$_{0.5}$O$_x$		18,000	600	~ 100	2016	98
Ni$_{0.5}$Ce$_{0.1}$Al$_{0.4}$O$_x$		18,000	550	~ 100	2016	98

**Catalysts**	**wt.%**	**WHSV (mL g_{cat}^{-1} h^{-1})**	**Temperature (°C)**	**NH_3 conversion (%)**	**Year**	**References**
$Ni_{0.61}(Mg_{0.6}Al_{0.3}O_n)$	41.8	30,000	650	~ 100	2017	[99]
$Ni/MgAl_2O_4$-LDO		30,000	650	~ 100	2022	[100]
K–$CoNi_{alloy}$–MgO–CeO_2–SrO		6000	450	97.7	2022	[101]
Co_7Mo_3/MCM-41	5	36,000	600	99.2	2012	[102]
MoO_3		15,000	650	98	2013	[103]
CoMo-I/γ-Al_2O_3	5	36,000	600	99	2014	[104]
MoN		15,000	650	100	2014	[105]

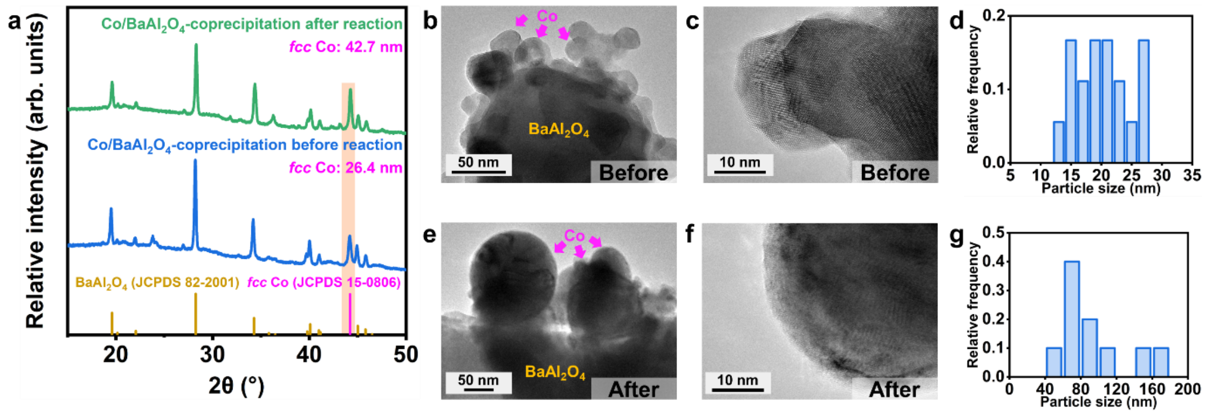

Supplementary Figure 27. Sintering of non-encapsulation structure in harsh thermal environments: Co/BaAl$_2$O$_4$-coprecipitation sample. Structure and configuration identification of Co/BaAl$_2$O$_4$-coprecipitation (see Supplementary Fig. 20 for synthesis details) before (blue) and after (green) 80-hour NH$_3$ decomposition reaction: **(a)** XRD patterns before and after the reaction. **(b-c)** TEM images before the reaction. **(d)** Co NP size distribution before the reaction measured from low magnification TEM. **(e-f)** TEM images after the reaction. **(g)** Co NP size distribution after the reaction measured from low magnification TEM images. Source data are provided as a Source Data file.

Supplementary Table 14

Estimation of Co NP crystallite sizes in Co/BaAl$_2$O$_4$-coprecipitation before and after 80-hour NH$_3$ decomposition reaction.

Sample	Phase (Peak selected for crystallite size calculation)	Co NP crystallite size from the Debye-Scherrer equation		Co NP size distribution from TEM, nm
		FWHM, °	Co NP crystallite size, nm	
Co/BaAl$_2$O$_4$- coprecipitation before reaction	Co (111)	0.325	26.4	15 ~ 28
Co/BaAl$_2$O$_4$-coprecipitation after reaction	Co (111)	0.201	42.7	40 ~ 120

Supplementary Fig. 27a shows the XRD patterns of Co/BaAl$_2$O$_4$-coprecipitation before and after the 80-hour NH$_3$ decomposition reaction. After 80-hour NH$_3$ decomposition reaction, fcc Co peaks in the XRD patterns become much narrower and sharper, indicating an increase in the Co NP crystallite size. Calculated from XRD patterns using the Debye-Scherrer equation and measured from the TEM images, the Co NP size increases significantly from 15 ~ 28 to 40 ~ 120 nm, revealing the severe aggregation of Co NPs in Co/BaAl$_2$O$_4$-coprecipitation during the reaction (Supplementary Fig. 27b-g and Supplementary Table 14).

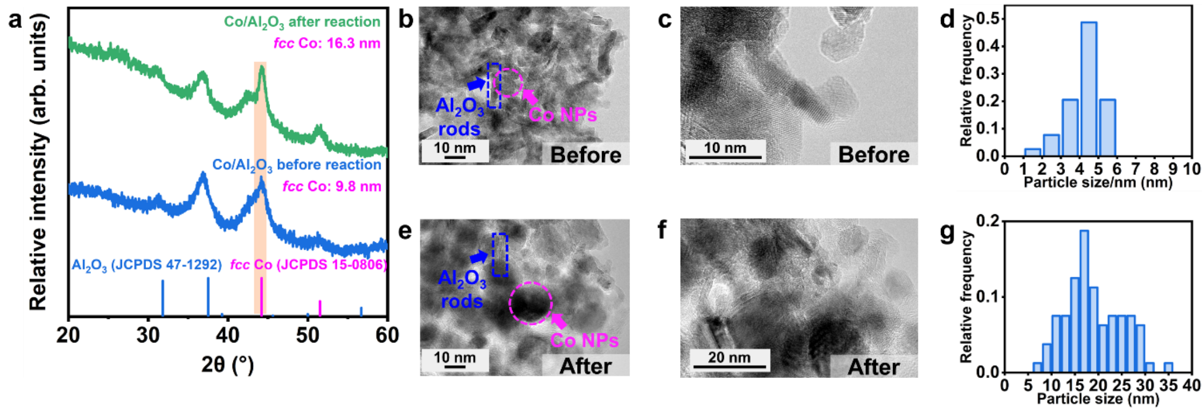

Supplementary Figure 28. Sintering of non-encapsulation structure in harsh thermal environments: Co/Al_2O_3 sample. Structure and configuration identification of Co/Al_2O_3 before (blue) and after (green) 80-hour NH_3 decomposition reaction: **(a)** XRD patterns before and after the reaction. **(b-c)** TEM images before the reaction. **(d)** Co NP size distribution before the reaction measured from low magnification TEM. **(e-f)** TEM images after the reaction. **(g)** Co NP size distribution after the reaction measured from low magnification TEM images. The Co/Al_2O_3 was synthesised by the following steps: (i) co-precipitation of $Co(NO_3)_2$ and $Al(NO_3)_3$ with the molar ratio of 2:3, i.e., 40Co:60Al; (ii) air calcination at 500 °C, i.e., C(500)-40Co:60Al; (iii) H_2 thermal treatment at 500 °C, i.e., C(500)-R(500)-40Co:60Al. Source data are provided as a Source Data file.

Supplementary Table 15

Estimation of Co NP crystallite size in Co/Al_2O_3 before and after 80-hour NH_3 decomposition reaction.

Sample	Phase (Peak selected for crystallite size calculation)	Co NP crystallite size from the Debye-Scherrer equation		Co NP size distribution from TEM, nm
		FWHM, °	Co NP crystallite size, nm	
Co/Al_2O_3 before reaction	Co (111)	0.589	9.8	3 ~ 6
Co/Al_2O_3 after reaction	Co (111)	0.603	16.3	15 ~ 30

Supplementary Fig. 28a shows the XRD patterns of Co/Al_2O_3 before and after the 80-hour NH_3 decomposition reaction. After 80-hour NH_3 decomposition reaction, fcc Co peaks in the XRD patterns become much narrower and sharper, indicating that the Co NPs are severely agglomerated during the reaction. Calculated from XRD patterns using the Debye-Scherrer equation and measured from the TEM images, the Co NP size increases significantly from 3 ~ 6 to 15 ~ 30 nm, revealing the severe aggregation of Co NPs in Co/Al_2O_3 during the reaction (Supplementary Fig. 28b-g and Supplementary Table 15).

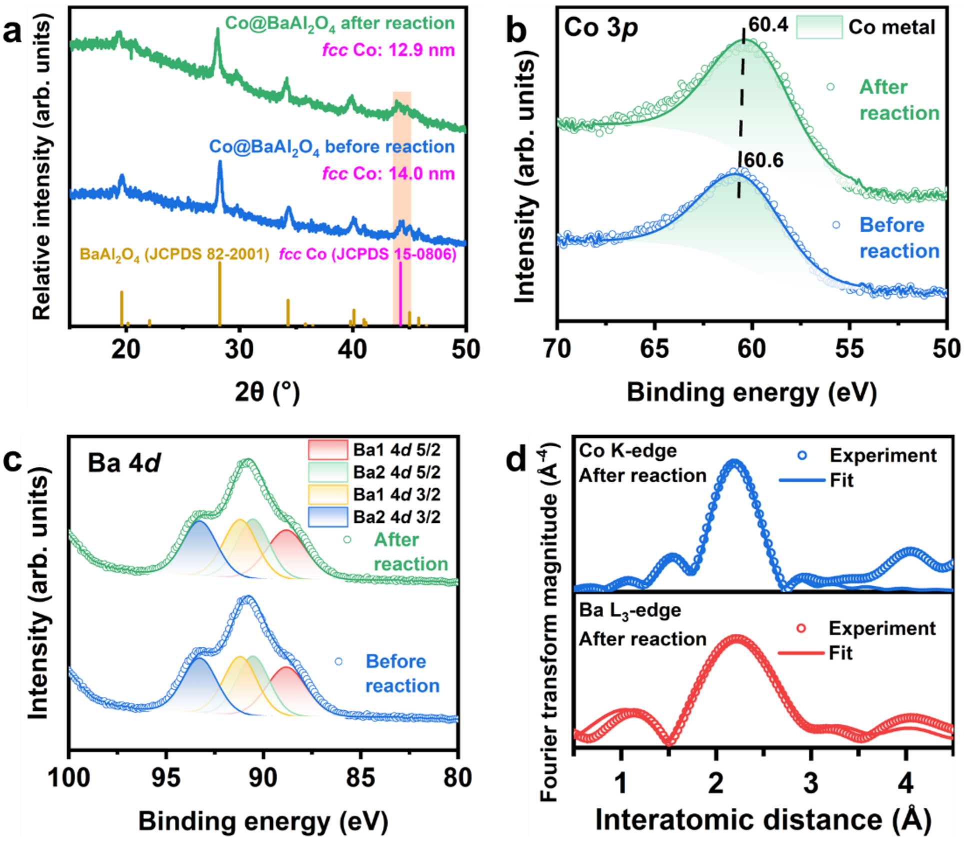

Supplementary Figure 29. Structural stability of Co@$BaAl_2O_4$ with encapsulation structure in harsh thermal environments. (a) XRD pattern of Co@$BaAl_2O_4$ catalysts before (blue) and after (green) 80-hour NH_3 decomposition reaction, **(b)** Co3*p* XPS spectra of Co@$BaAl_2O_4$ catalysts before (blue) and after (green) 80-hour NH_3 decomposition reaction, blank circles represent experimental XPS data, and solid lines are the convolution of Gaussians and Lorentzians, **(c)** Ba4*d* XPS spectra of Co@$BaAl_2O_4$ catalysts before (blue) and after (green) 80-hour NH_3 decomposition reaction, blank circles represent experimental XPS data, and solid lines are the convolution of Gaussians and Lorentzians, and **(d)** k^3-weighted Fourier transforms (FT) Co K-edge (upper panel) and Ba L_3-edge (lower panel) EXAFS of post-reaction Co@$BaAl_2O_4$ and corresponding EXAFS fitting: circles represent experimental data, and solid lines represent the EXAFS fitting results. Source data are provided as a Source Data file.

Supplementary Table 16

Estimation of Co NP crystallite sizes in Co@$BaAl_2O_4$ before and after 80-hour NH_3 decomposition reaction.

Sample	Phase (Peak selected for crystallite size calculation)	Co NP crystallite size from the Debye-Scherrer equation		Co NP size distribution from TEM, nm
		FWHM, °	Co NP crystallite size, nm	
Co@$BaAl_2O_4$ before reaction	Co (111)	0.613	14.0	10 ~ 20
Co@$BaAl_2O_4$ after reaction	Co (111)	0.667	12.9	10 ~ 20

Supplementary Table 17

Co K-edge EXAFS fitting parameters of post-reaction Co@$BaAl_2O_4$.

Stage	Bond	CN	R, Å	D-W factor (σ^2), Å^2	ΔE_0, eV	R-factor, %
Post-reaction Co@$BaAl_2O_4$	Co-Co	8.2 (2)	2.48 (1)	0.009 (1)	5.7	1.9

*Co K-edge EXAFS fitting results, in which CN is the average coordination number, R is the distance from the absorber atom, and σ^2 the Debye–Waller factor. R-factor denotes the quality factor of the fitting and ΔE_0 the energy shift from the absorption edge energy E_0.

Supplementary Table 18

Ba L_3-edge EXAFS fitting parameters of post-reaction Co@$BaAl_2O_4$.

Stage	Bond	CN	R, Å	D-W factor (σ^2), Å^2	ΔE_0, eV	R-factor, %
Post-reaction Co@$BaAl_2O_4$	Ba-O	6.0 (3)	2.84 (2)	0.009 (1)	6.8	1.9
	Ba-O	1.6 (2)	3.08 (3)	0.014 (4)		
	Ba-Al	5.0 (5)	3.52 (1)	0.009 (1)	8.2	
	Ba-Al	6.2 (3)	3.91 (1)	0.011 (3)		

*Ba L_3-edge EXAFS fitting results, in which CN is the average coordination number, R is the distance from the absorber atom, and σ^2 the Debye–Waller factor. R-factor denotes the quality factor of the fitting and ΔE_0 the energy shift from the absorption edge energy E_0.

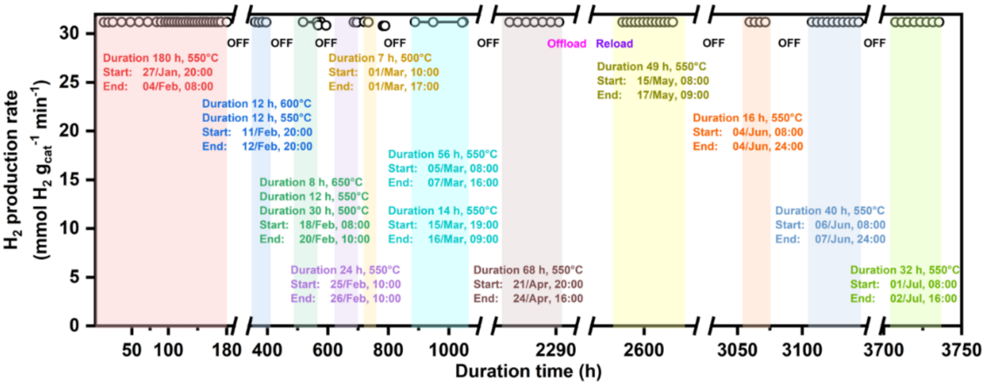

Supplementary Figure 30. Excellent environment tolerance and reusability for practical application. NH_3 conversion during the five-month-long testing events, including temperature fluctuations, system ONs/OFFs, and catalyst offload/reload. Source data are provided as a Source Data file.

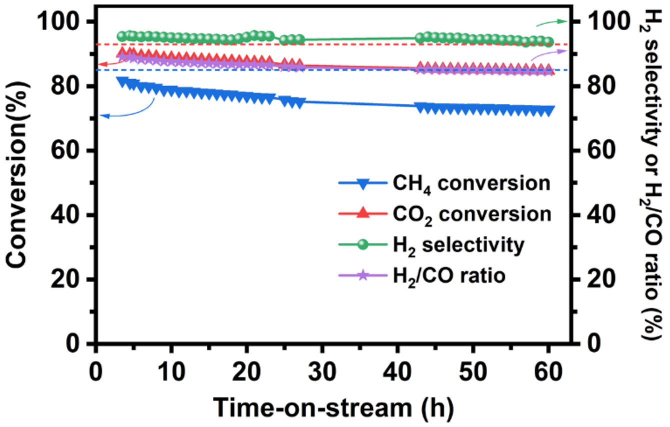

Supplementary Figure 31. Good stability of Co@$BaAl_2O_4$ with encapsulation structure in CH_4 dry-reforming reaction. CH_4 conversion, CO_2 conversion, H_2 selectivity and H_2/CO ratio in different time-on-stream during the CH_4 dry-reforming reaction over the Co@$BaAl_2O_4$ catalyst. The performance was evaluated at WHSV of 31,765 mL g_{cat}^{-1} h^{-1} and a temperature of 750 °C. The molar ratio of CH_4:CO_2:Ar in inlet gas is 0.45:0.45:0.10. Blue and red dotted lines indicate the thermodynamic equilibrium of CH_4 and CO_2 conversions, respectively. Source data are provided as a Source Data file.

Supplementary Table 19

Reported catalysts used for CH_4 dry-reforming and their catalytic performances at corresponding conditions.

Catalyst	Reactants	Condition	Performance and Stability	Time-on-stream	Year	Ref.
$Co@BaAl_2O_4$	$CH_4 : CO_2 : Ar = 0.45:0.45:0.1$	750 °C<br>31,765 $mL \cdot g_{cat}^{-1} \cdot h^{-1}$	Initial: $X(CO_2) = 90.1\%$; $X(CH_4) = 81.8\%$; $H_2/CO = 0.89$<br>Final: $X(CO_2) = 85.3\%$; $X(CH_4) = 73.6\%$; $H_2/CO = 0.847$	60 h	This work	
Ni/La_2O_3	$CH_4 : CO_2 = 1:1$	800°C<br>20,000 $mL \cdot g_{cat}^{-1} \cdot h^{-1}$	Initial: $X(CO_2) = 74.7\%$; $X(CH_4) = 64.6\%$<br>Final: $X(CO_2) = 84.5\%$; $X(CH_4) = 68.5\%$	24 h	2022	106
$Ni_3Co_9/Mg(Al)O$	$CH_4 : CO_2 : N_2 = 1:1:2$	750 °C<br>60,000 $mL \cdot g_{cat}^{-1} \cdot h^{-1}$	Initial: $X(CO_2) = 92.3\%$; $X(CH_4) = 85.6\%$; $H_2/CO = 0.66$<br>Final: $X(CO_2) = 92.3\%$; $X(CH_4) = 84.7\%$; $H_2/CO = 0.651$	25 h	2022	107
$Ni/ZrO_2@BN$	$CH_4 : CO_2 = 1:1$	750 °C<br>25,000 $mL \cdot g_{cat}^{-1} \cdot h^{-1}$	Initial: $X(CO_2) = 78.5\%$; $X(CH_4) = 70.0\%$; $H_2/CO = 0.964$<br>Final: $X(CO_2) = 80.7\%$; $X(CH_4) = 70.3\%$; $H_2/CO = 0.944$	200 h	2022	108
$2Ni/H\text{-}Al_2O_3$	$CH_4 : CO_2 : N_2 = 1:1:1$	700 °C<br>36,000 $mL \cdot g_{cat}^{-1} \cdot h^{-1}$	Initial: $X(CO_2) = 86.0\%$; $X(CH_4) = 77.6\%$<br>Final: $X(CO_2) = 82.6\%$; $X(CH_4) = 74.3\%$	130 h	2022	109
NMG-600	$CH_4 : CO_2 : N_2 = 1:1:1$	600 °C<br>54,000 $mL \cdot g_{cat}^{-1} \cdot h^{-1}$	Initial: $X(CO_2) = 53.2\%$; $X(CH_4) = 50.2\%$<br>Final: $X(CO_2) = 48.6\%$; $X(CH_4) = 45.3\%$	70 h	2021	110
$Ni_{9.6}\text{-}V@HSS$	$CH_4 : CO_2 : N_2 = 1:1:2$	700 °C<br>192,000 $mL \cdot g_{cat}^{-1} \cdot h^{-1}$	Initial: $X(CO_2) = 74.6\%$; $X(CH_4) = 71\%$<br>Final: $X(CO_2) = 71.8\%$; $X(CH_4) = 69.8\%$	50 h	2021	111
10Co/HAP_N	$CH_4 : CO_2 : N_2 = 20:20:60$	700 °C<br>31,765 $mL \cdot g_{cat}^{-1} \cdot h^{-1}$	Initial: $X(CO_2) = 66.7\%$; $X(CH_4) = 59.6\%$<br>Final: $X(CO_2) = 62.6\%$; $X(CH_4) = 51.7\%$	50 h	2020	112
Ni-Co/MgO/monolith	$CH_4 : CO_2 : N_2 = 30:30:10$	750 °C<br>84,000 $mL \cdot g_{cat}^{-1} \cdot h^{-1}$	Initial: $X(CO_2) = 89.1\%$; $X(CH_4) = 86.0\%$<br>Final: $X(CO_2) = 89.2\%$; $X(CH_4) = 85.5\%$	8 h	2020	113
$Ni@BO_x/h\text{-}BN$	$CH_4 : CO_2 : N_2 = 0.4:0.4:0.2$	750 °C<br>60,000 $mL \cdot g_{cat}^{-1} \cdot h^{-1}$	Initial: $X(CO_2) = 80.4\%$; $X(CH_4) = 72.5\%$<br>Final: $X(CO_2) = 79.9\%$; $X(CH_4) = 71.9\%$	40 h	2020	114
Ni/DMS	$CH_4 : CO_2 = 1:1$	700 °C<br>18,000 $mL \cdot g_{cat}^{-1} \cdot h^{-1}$	Initial: $X(CO_2) = 85.0\%$; $X(CH_4) = 76.7\%$<br>Final: $X(CO_2) = 84.1\%$; $X(CH_4) = 74.7\%$	145 h	2019	115
$2Ni_1/HAP\text{-}Ce$	$CH_4 : CO_2 : He = 10:10:30$	750 °C<br>60,000 $mL \cdot g_{cat}^{-1} \cdot h^{-1}$	Initial: $X(CO_2) = 89.8\%$; $X(CH_4) = 84.2\%$<br>Final: $X(CO_2) = 83.3\%$; $X(CH_4) = 75.5\%$	100 h	2019	116
Co-Ni/HAP_SIWI	$CH_4 : CO_2 : Ar = 20:20:50$	700 °C<br>15,882 $mL \cdot g_{cat}^{-1} \cdot h^{-1}$	Initial: $X(CO_2) = 81.3\%$; $X(CH_4) = 76.7\%$<br>Final: $X(CO_2) = 69.2\%$; $X(CH_4) = 61.8\%$	50 h	2018	117

Catalyst	Reactants	Condition	Performance and Stability	Time-on-stream	Year	Ref.
$Sn_{0.02}Ni/Ce\text{-}Al$	$CH_4 : CO_2 : N_2$ = 1:1:6	700 °C<br>60,000 $mL \cdot g_{cat}^{-1} \cdot h^{-1}$	Initial: $X(CO_2)$ = 90.7%; $X(CH_4)$ = 79.3%; H_2/CO =0.893<br>Final: $X(CO_2)$ = 66.8%; $X(CH_4)$ = 20.5%; H_2/CO = 0.760	20 h	2018	118
$Ru_{0.035}Ni_{0.035}Mg_{0.93}O\text{-}DR$	$CH_4 : CO_2$ = 25:25	760 °C<br>86,000 $mL \cdot g_{cat}^{-1} \cdot h^{-1}$	Initial: $X(CO_2)$= 88.9%; $X(CH_4)$= 82.2%; H_2/CO =0.847<br>Final: $X(CO_2)$ = 87.0%; $X(CH_4)$ = 80.7%; H_2/CO =0.842	100 h	2018	119
$7Ni3Co/SiO_2$	$He : CH_4 : CO_2$ = 1:1:1	750 °C<br>60,000 $mL \cdot g_{cat}^{-1} \cdot h^{-1}$	Initial: $X(CO_2)$ = 87.4 %; $X(CH_4)$ = 84.1%; H_2/CO = 0.814<br>Final: $X(CO_2)$ = 87.3%; $X(CH_4)$ = 83.3%; H_2/CO = 0.805	100 h	2017	120
$Ni/Ce_{0.8}Pr_{0.2}O_{2-\delta}$	$CH_4 : CO_2 : He$ = 20:20:60	750 °C<br>30,000 $mL \cdot g_{cat}^{-1} \cdot h^{-1}$	Initial: $X(CO_2)$ = 83.3%; $X(CH_4)$ = 74.7%<br>Final: $X(CO_2)$ = 82.6%; $X(CH_4)$ = 72.6%	25 h	2016	121
$Co_{0.3}Ni_{9.7}Mg_{90}O$	$CH_4:CO_2:N_2$ = 25:25:18	760 °C<br>60,000 $mL \cdot g_{cat}^{-1} \cdot h^{-1}$	Initial: $X(CO_2)$= 84.0%; $X(CH_4)$ = 76.7%<br>Final: $X(CO_2)$ = 72.3%; $X(CH_4)$ = 65.7%	1800 h	2015	122
145%Co–2%Ce/ZrO_2	$CH_4 : CO_2$ = 1:1	600 °C<br>20,000 $mL \cdot g_{cat}^{-1} \cdot h^{-1}$	Initial: $X(CH_4)$ = 39.9%<br>Final: $X(CH_4)$ = 38.5%	70 h	2015	123
Co-Mo/ZrO_2	$CH_4 : CO_2$ = 1:1	850 °C<br>4,800 $mL \cdot g_{cat}^{-1} \cdot h^{-1}$	Initial: $X(CO_2)$ = 98.02%; $X(CH_4)$ = 97.75%<br>Final: $X(CO_2)$ = 97.07%; $X(CH_4)$ = 97.50%	4 h	2014	124
Co/TiO_2	$CH_4 : CO_2$ = 1:1	750 °C<br>6,000 $mL \cdot g_{cat}^{-1} \cdot h^{-1}$	Initial: $X(CH_4)$ = 65.7%<br>Final: $X(CH_4)$ = 47.3%	24 h	2005	125

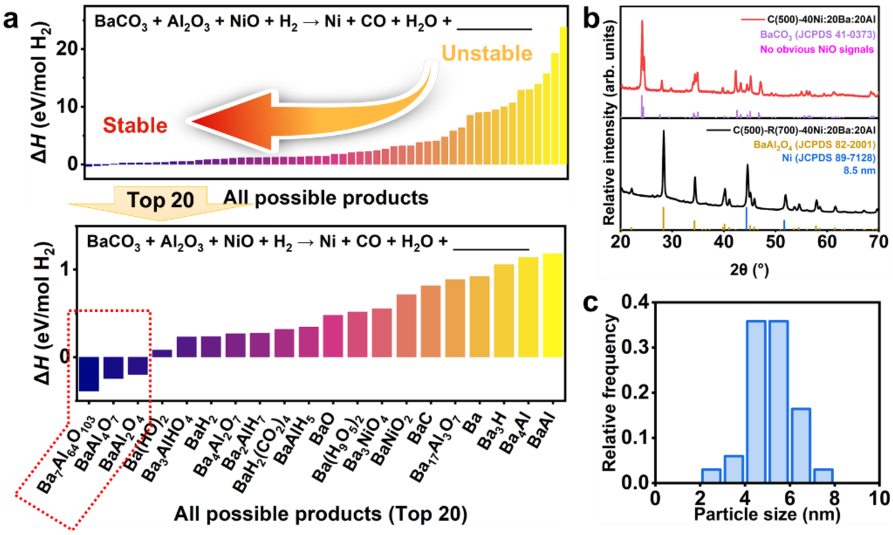

Supplementary Figure 32. Ni@BaAl$_2$O$_4$ core@shell synthesis. (a) All (upper panel) and top 20 (lower panel) possible products and corresponding ΔH of the solid-state reactions between BaCO$_3$ + Al$_2$O$_3$ + NiO + H$_2$ (Detailed data can be found in Source Data files). **(b)** XRD pattern of C(500)-40Ni:20Ba:40Al and Ni@BaAl$_2$O$_4$. **(c)** Size distribution of Ni NPs measured from low magnification TEM images of Ni@BaAl$_2$O$_4$. Source data are provided as a Source Data file.

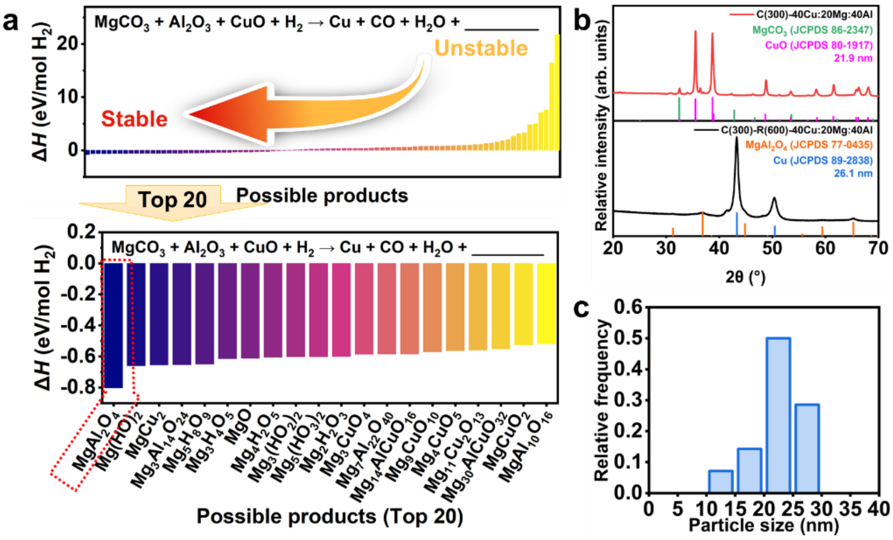

Supplementary Figure 33. Cu@MgAl$_2$O$_4$ core@shell synthesis. (a) All (upper panel) and top 20 (lower panel) possible products and corresponding reaction enthalpy change (ΔH) of the solid-state reactions between MgCO$_3$ + Al$_2$O$_3$ + CuO + H$_2$ (Detailed data can be found in Source Data files). **(b)** XRD pattern of C(300)-40Cu:20Mg:40Al and Cu@MgAl$_2$O$_4$. **(c)** Size distribution of Cu NPs measured from low magnification TEM images of Cu@MgAl$_2$O$_4$. Source data are provided as a Source Data file.

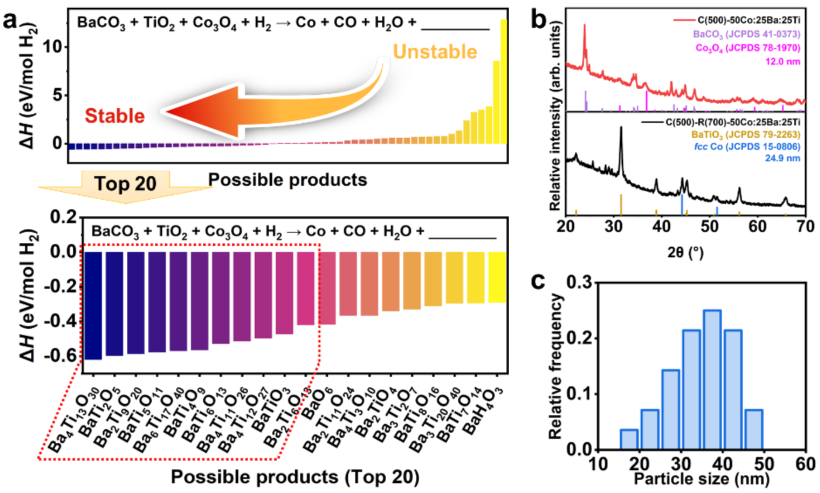

Supplementary Figure 34. Co@BaTiO$_3$ core@shell synthesis. (a) All (upper panel) and top 20 (lower panel) possible products and corresponding reaction enthalpy change (ΔH) of the solid-state reactions between BaCO$_3$ + TiO$_2$ + CoO + H$_2$ (Detailed data can be found in Source Data files). **(b)** XRD pattern of C(500)-50Co:25Ba:25Ti and Co@BaTiO$_3$. **(c)** Size distribution of Co NPs measured from low magnification TEM images of Co@BaTiO$_3$. Source data are provided as a Source Data file.

Supplementary Table 20

Estimation of M_T NPs crystallite sizes.

Sample	Phase (Peak selected for crystallite size calculation)	Crystallite size from the Debye-Scherrer equation		M_T NP size distribution from TEM, nm
		FWHM, °	Crystallite size, nm	
**Ni@BaAl$_2$O$_4$ core@shell**	Ni (111)	1.002	8.5	4 ~ 8
**Cu@MgAl$_2$O$_4$ core@shell**	Cu(111)	0.327	26.1	20 ~ 30
**Co@BaTiO$_3$ core@shell**	Co (111)	0.344	24.9	25 ~ 40

Like the example of BaCO$_3$ + Al$_2$O$_3$ + Co$_3$O$_4$ + H$_2$ (Supplementary Fig. 9), all the possible products and corresponding ΔH of the reactions MgCO$_3$ + Al$_2$O$_3$ + CuO + H$_2$ have been sorted and plotted in Supplementary Fig. 33a, which indicates that MgAl$_2$O$_4$ shows the lowest ΔH among all the product considered in MgCO$_3$ + Al$_2$O$_3$ + CuO + H$_2$ reactions. Hence MgAl$_2$O$_4$ is chosen as the target overlayer for Cu NPs. On the other hand, titanate overlayer formation can also be realised when considering the solid-state reaction between BaCO$_3$ and TiO$_2$. Different titanate products with stoichiometric ratios are evaluated using the rapid computational screening method (Supplementary Fig. 34a), in which BaTiO$_3$ is one of the most commonly reported ones[126]. Hence BaTiO$_3$, with Ba/Ti = 1/1, was chosen as another target overlayer for Co NPs.

Guided by our proposed rationale in Fig. 1b, we prepared 40Ni:20Ba:40Al, 40Cu:20Mg:40Al, and 50Co:25Ba:25Ti precursors. After the thermal treatment in H$_2$, the formation of M_T NPs (Ni, Cu, or Co) and the corresponding M_{AE}Al$_2$O$_4$ (BaAl$_2$O$_4$, MgAl$_2$O$_4$ or BaTiO$_3$) can be confirmed by the characteristic XRD patterns (Supplementary Figs. 32b, 33b, and 34b). STEM and energy dispersive X-ray (EDS) mapping clearly visualize the encapsulation structures of Ni@BaAl$_2$O$_4$, Cu@MgAl$_2$O$_4$, and Co@BaTiO$_3$, respectively, with the well-rounded alkaline earth metal aluminates overlayer in close contact with the M_T NPs (Fig. 4e). The encapsulated M_T NPs show relatively uniform size distributions in the range of 4~8, 20~30, and 24~40 nm, for Ni, Cu, and Co, respectively, in line with the crystal size derived from XRD results (Supplementary Figs. 32c, 33c, 34c, and Supplementary Table 20).

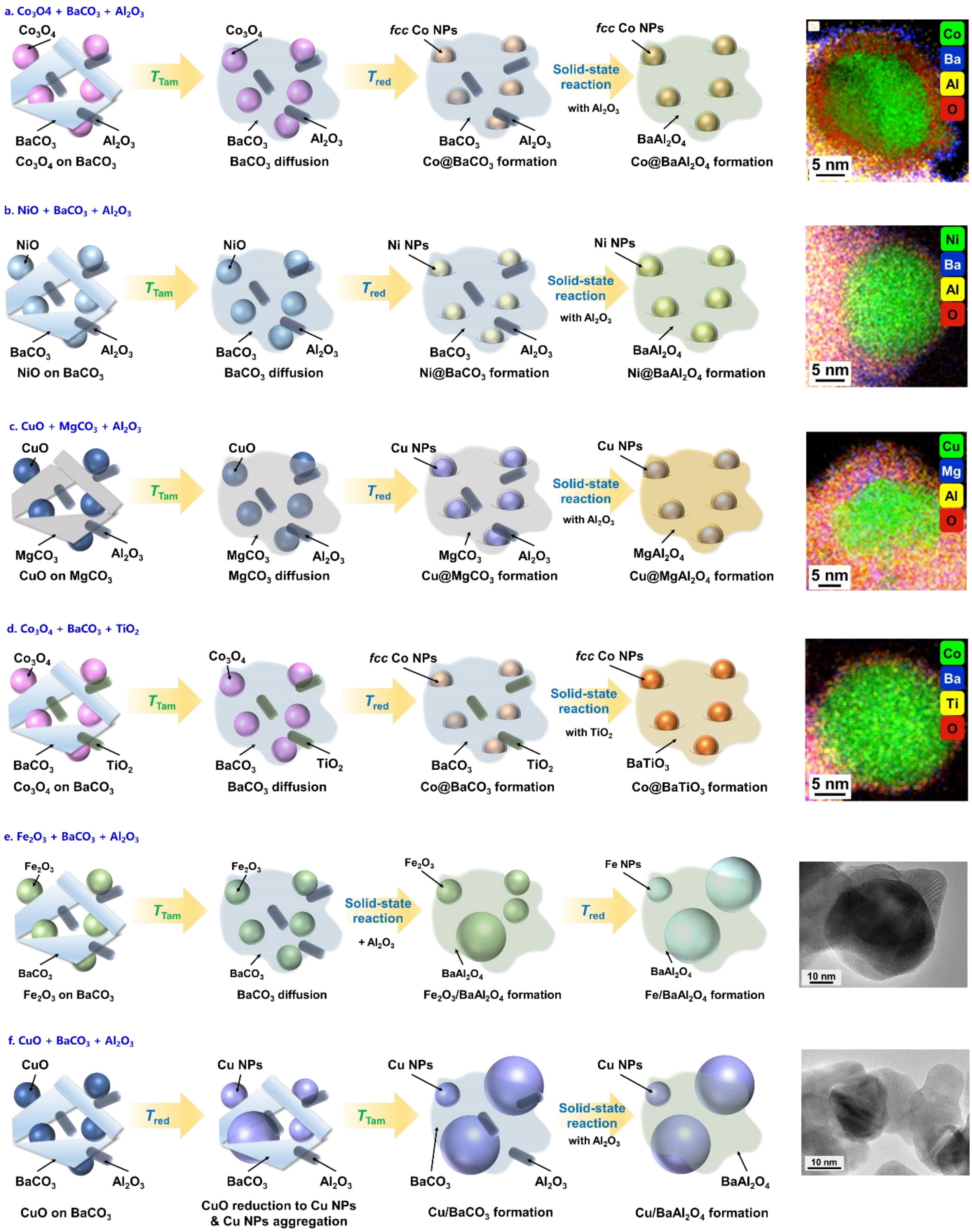

Supplementary Figure 35. Schematic illustration of the designed synthesis rationale for all the successful core@shell examples, as well as two control examples falling either outside or on the edge of the encapsulable window. **(a)** $Co_3O_4 + BaCO_3 + Al_2O_3$, **(b)** $NiO + BaCO_3 + Al_2O_3$, **(c)** $CuO + MgCO_3 + Al_2O_3$, **(d)** $Co_3O_4 + BaCO_3 + TiO_2$, **(e)** $Fe_2O_3 + BaCO_3 + Al_2O_3$, and **(f)** $CuO + BaCO_3 + Al_2O_3$.

Supplementary References

1 Protochips. *Atmosphere: TEM environmental gas cell*, <https://www.protochips.com/products/atmosphere/> (2023).

2 Zhai, L. *et al.* From lab to practical: An ammonia-powered fuel cell electric golf cart system. *Chem. Eng. J.* **452** (2023).

3 Xie, P. *et al.* Highly efficient decomposition of ammonia using high-entropy alloy catalysts. *Nat. Commun.* **10**, 4011 (2019).

4 Zhang, X. *et al.* Ru Nanoparticles on Pr_2O_3 as an Efficient Catalyst for Hydrogen Production from Ammonia Decomposition. *Catal. Lett.* **152**, 1170-1181 (2021).

5 Zhou, H. *et al.* Two-dimensional molybdenum carbide 2D-Mo_2C as a superior catalyst for CO_2 hydrogenation. *Nat. Commun.* **12**, 5510 (2021).

6 Gevers, L. E. *et al.* Unraveling the structure and role of Mn and Ce for NO_x reduction in application-relevant catalysts. *Nat. Commun.* **13**, 2960 (2022).

7 Pozzo, A. D., Armutlulu, A., Rekhtina, M., Abdala, P. M. & Müller, C. R. CO_2 uptake and cyclic stability of MgO-Based CO_2 sorbents promoted with alkali metal nitrates and their eutectic mixtures. *ACS Appl. Energy Mater.* **2**, 1295-1307 (2019).

8 Phromprasit, J., Powell, J. & Assabumrungrat, S. Metals (Mg, Sr and Al) modified CaO based sorbent for CO_2 sorption/desorption stability in fixed bed reactor for high temperature application. *Chem. Eng. J.* **284**, 1212-1223 (2016).

9 Antao, S. M. & Hassan, I. $BaCO_3$: high-temperature crystal structures and the Pmcn → R3m phase transition at 811°C. *Phys. Chem. Miner.* **34**, 573-580 (2007).

10 Argyle, M. & Bartholomew, C. Heterogeneous catalyst deactivation and regeneration: A review. *Catalysts* **5**, 145-269 (2015).

11 Hu, S. & Li, W.-X. Sabatier principle of metal-support interaction for design of ultrastable metal nanocatalysts. *Science* **374**, 1360-1365 (2021).

12 Seeburg, D. *et al.* Structural changes of highly active Pd/MeO_x (Me = Fe, Co, Ni) during catalytic methane combustion. *Catalysts* **8**, 42 (2018).

13 Ma, Z., Xiao, R. & Zhang, H. Montmorillonite-supported iron oxide for hydrogen storage by chemical looping. *Energy Technol.* **5**, 1399-1406 (2017).

14 Han, Q. *et al.* Improved catalytic performance of Au/α-Fe_2O_3-like-worm catalyst for low temperature CO oxidation. *Nanomaterials* **9** (2019).

15 Chamoumi, M. & Abatzoglou, N. $NiFe_2O_4$ production from α-Fe_2O_3 via improved solid state reaction: Application as catalyst in CH_4 dry reforming. *Can. J. Chem. Eng.* **94**, 1801-1808 (2016).

16 Musolino, M. G., Busacca, C., Mauriello, F. & Pietropaolo, R. Aliphatic carbonyl reduction promoted by palladium catalysts under mild conditions. *Appl. Catal. A: Gen.* **379**, 77-86 (2010).

17 Zhao, G. *et al.* Facile synthesis of Mn-doped Fe_2O_3 nanostructures: enhanced CO catalytic performance induced by manganese doping. *New J. Chem.* **40**, 3491-3498 (2016).

18 Liu, Q. *et al.* Construction of urchin-structured Fe_2O_3 microspheres supported potassium for diesel soot catalytic elimination. *Fuel* **306**, 121661 (2021).

19 Tahari, M. N. A. *et al.* Influence of hydrogen and various carbon monoxide concentrations on reduction behavior of iron oxide at low temperature. *Int. J. Hydrog. Energy* **44**, 20751-20759 (2019).

20 Zieliński, J., Zglinicka, I., Znak, L. & Kaszkur, Z. Reduction of Fe_2O_3 with hydrogen. *Appl. Catal. A: Gen.* **381**, 191-196 (2010).

21 Wei, X., Zhou, Y., Li, Y. & Shen, W. Polymorphous transformation of rod-shaped iron oxides and their catalytic properties in selective reduction of NO by NH_3. *RSC Adv.* **5**, 66141-66146 (2015).

22 Kim, S. S., Lee, S. M. & Hong, S. C. A study on the reaction characteristics of CO_2 decomposition using iron oxides. *J. Ind. Eng. Chem.* **18**, 860-864 (2012).

23 Gómez-Cuaspud, J. A. & Schmal, M. Effect of metal oxides concentration over supported cordierite monoliths on the partial oxidation of ethanol. *Appl. Catal. B: Environ.* **148-149**, 1-10 (2014).

24 Choya, A., Rivas, B. d., Gutiérrez-Ortiz, J. I., González-Velasco, J. R. & López-Fonseca, R. Synthesis, characterization and kinetic behavior of supported cobalt catalysts for oxidative after-treatment of methane lean mixtures. *Materials* **12**, 3174 (2019).

25 Xiong, M. *et al.* In situ tuning of electronic structure of catalysts using controllable hydrogen spillover for enhanced selectivity. *Nat. Commun.* **11**, 4773 (2020).

26 Silva, P. H., Oliveira, H. S. & Batista, M. S. Redox effects in Cu, Co or Fe in oxides nanocrystals with high catalytic activity for the acetonitrile combustion. *SN Appl. Sci.* **2** (2020).

27 Wang, Y., Zhang, C., Liu, F. & He, H. Well-dispersed palladium supported on ordered mesoporous Co_3O_4 for catalytic oxidation of o-xylene. *Appl. Catal. B: Environ.* **142-143**, 72-79 (2013).

28 Li, J. *et al.* Promotional role of ceria on cobaltosic oxide catalyst for low-temperature CO oxidation. *Catal. Sci. Technol.* **2**, 1865-1871 (2012).

29 Fang, J. *et al.* Activation of oxygen on the surface of the Co_3O_4 catalyst by single-atom Ag toward efficient catalytic benzene combustion. *J. Phys. Chem. C* **126**, 5873-5884 (2022).
30 Zigla, A. A. *et al.* Magnesium-modified Co_3O_4 catalyst with remarkable performance for toluene low temperature deep oxidation. *Catalysts* **12**, 411 (2022).
31 Wu, M. *et al.* Investigation of synergistic effects and high performance of La-Co composite oxides for toluene catalytic oxidation at low temperature. *Environ. Sci. Pollut. Res.* **26**, 12123-12135 (2019).
32 Li, M. *et al.* Effect of residual chlorine on the catalytic performance of Co_3O_4 for CO oxidation. *ACS Catal.* **9**, 11676-11684 (2019).
33 Yao, Y. *et al.* Enhanced low temperature CO oxidation by pretreatment: specialty of the Au–Co_3O_4 oxide interfacial structures. *Catal. Sci. Technol.* **6**, 2349-2360 (2016).
34 Wang, X., Wen, W., Mi, J., Li, X. & Wang, R. The ordered mesoporous transition metal oxides for selective catalytic reduction of NO_x at low temperature. *Appl. Catal. B: Environ.* **176-177**, 454-463 (2015).
35 Jha, A., Jeong, D.-W., Lee, Y.-L., Nah, I. W. & Roh, H.-S. Enhancing the catalytic performance of cobalt oxide by doping on ceria in the high temperature water–gas shift reaction. *RSC Adv.* **5**, 103023-103029 (2015).
36 Chen, K. *et al.* Hydroxyl groups attached to Co^{2+} on the surface of Co_3O_4: a promising structure for propane catalytic oxidation. *Catal. Sci. Technol.* **10**, 2573-2582 (2020).
37 Sun, Y.-M. *et al.* Producing hydrogen from steam reforming of bio-oil derived oxygenated model compounds by utilizing Ce-modified Ni/attapulgite catalysts. *Catal. Lett.* **152**, 324-339 (2021).
38 Xu, X. *et al.* Engineering Ni^{3+} cations in NiO Lattice at the atomic level by Li^+ doping: the roles of Ni^{3+} and oxygen species for CO oxidation. *ACS Catal.* **8**, 8033-8045 (2018).
39 Yen, H. & Kleitz, F. High-performance solid catalysts for H_2 generation from ammonia borane: progress through synergetic Cu–Ni interactions. *J. Mater. Chem. A* **1**, 14790-14796 (2013).
40 Jafarbegloo, M., Tarlani, A., Mesbah, A. W. & Sahebdelfar, S. One-pot synthesis of NiO–MgO nanocatalysts for CO_2 reforming of methane: The influence of active metal content on catalytic performance. *J. Nat. Gas Sci. Eng.* **27**, 1165-1173 (2015).
41 Chu, W., Yang, W. & Lin, L. Selective oxidation of methane to syngas over NiO/barium hexaaluminate. *Catal. Lett.* **74**, 139-143 (2001).
42 Wang, J. *et al.* Nb_2O_5 modified $NiAl_2O_4$ catalysts for hydrodeoxygenation of methyl palmitate to long-chain alkane. *Biomass Convers. Biorefin.* (2022).
43 Wang, Y. *et al. In situ* synthesis of Ni/NiO composites with defect-rich ultrathin nanosheets for highly efficient biomass-derivative selective hydrogenation. *J. Mater. Chem. A* **7**, 17834-17841 (2019).
44 Zhang, X. *et al.* Effect of surface composition and structure of the mesoporous Ni/KIT-6 catalyst on catalytic hydrodeoxygenation performance. *Catalysts* **9**, 889 (2019).
45 Jehng, J.-M. & Chen, C.-M. Amination of polyethylene glycol to polyetheramine over the supported nickel catalysts. *Catal. Lett.* **77**, 147-154 (2001).
46 Mock, S. A., Zell, E. T., Hossain, S. T. & Wang, R. Effect of reduction treatment on CO oxidation with CeO_2 nanorod-supported CuO_x catalysts. *ChemCatChem* **10**, 311-319 (2017).
47 Kerkar, R. D. & Salker, A. V. A route to develop the synergy between CeO_2 and CuO for low temperature CO oxidation. *Catal. Lett.* **150**, 2774-2783 (2020).
48 Pires, C. A., Santos, A. C. C. d. & Jordão, E. Oxidation of phenol in aqueous solution with copper oxide catalysts supported on γ-Al_2O_3, pillared clay and TiO_2: comparison of the performance and costs associated with each catalyst. *Brazilian J. Chem. Eng.* **32**, 837-848 (2015).
49 Chen, H., Xu, Y., Zhu, K. & Zhang, H. Understanding oxygen-deficient $La_2CuO_{4-\delta}$ perovskite activated peroxymonosulfate for bisphenol A degradation: The role of localized electron within oxygen vacancy. *Appl. Catal. B: Environ.* **284**, 119732 (2021).
50 Wang, Z. *et al.* Enhancing the ethynylation performance of CuO-Bi_2O_3 nanocatalysts by tuning Cu-Bi interactions and phase structures. *Catalysts* **9**, 35 (2019).
51 Karnaukhov, T. M., Veselov, G. B., Cherepanova, S. V. & Vedyagin, A. A. Sol-gel synthesis and characterization of the Cu-Mg-O system for chemical looping application. *Materials* **15**, 2021 (2022).
52 Li, X. *et al.* The support effects on the direct conversion of syngas to higher alcohol synthesis over copper-based catalysts. *Catalysts* **9**, 199 (2019).
53 Zhang, M. *et al.* Low-temperature catalytic oxidation of benzene over nanocrystalline Cu–Mn composite oxides by facile sol–gel synthesis. *New J. Chem.* **44**, 2442-2451 (2020).
54 Li, J. *et al.* One-dimensional Cu-based catalysts with layered Cu–Cu_2O–CuO walls for the Rochow reaction. *Nano Res.* **9**, 1377-1392 (2016).
55 Khan, W. U. *et al.* Catalytically active interfaces in titania nanorod-supported copper catalysts for CO oxidation. *Nano Res.* **13**, 533-542 (2020).
56 Huo, C., Ouyang, J. & Yang, H. CuO nanoparticles encapsulated inside Al-MCM-41 mesoporous materials via direct synthetic route. *Sci. Rep.* **4**, 3682 (2014).

57 Basahel, S. N. *et al.* Physico-chemical and catalytic properties of mesoporous CuO-ZrO_2 catalysts. *Catalysts* **6**, 57 (2016).
58 Jain, A. *et al.* Formation enthalpies by mixing GGA and GGA + *U* calculations. *Phys. Rev. B* **84**, 045115 (2011).
59 Allix, M. *et al.* Highly transparent $BaAl_4O_7$ polycrystalline ceramic obtained by full crystallization from glass. *Adv. Mat.* **24**, 5570-5575 (2012).
60 Fedorenko, O. Y., Lisachuk, H. V., Bilohubkina, K. V. & Kryvobok, R. V. in *Actual Problems of Natural Sciences: Modern Scientific Discussions* 373-403 (Baltija Publishing, 2021).
61 Rafiaei, S. M., Dini, G. & Bahrami, A. Synthesis, crystal structure, optical and adsorption properties of $BaAl_2O_4$: Eu^{2+}, Eu^{2+}/L^{3+} (L = Dy, Er, Sm, Gd, Nd, and Pr) phosphors. *Ceram. Int.* **46**, 20243-20250 (2020).
62 Vrankić, M., Gržeta, B., Lützenkirchen-Hecht, D., Bosnar, S. & Šarić, A. Chromium environment within Cr-doped $BaAl_2O_4$: Correlation of x-ray diffraction and x-ray absorption spectroscopy investigations. *Inorg. Chem.* **54**, 11127-11135 (2015).
63 Jain, S., Shah, J., Negi, N. S., Sharma, C. & Kotnala, R. K. Significance of interface barrier at electrode of hematite hydroelectric cell for generating ecopower by water splitting. *Int. J. Energy Res.* **43**, 4743-4755 (2019).
64 Casapu, M. *et al.* Formation and stability of barium aluminate and cerate in NOx storage-reduction catalysts. *Appl. Catal. B: Environ.* **63**, 232-242 (2006).
65 Ghavami, M., Soltan, J. & Chen, N. Synthesis of MnOx/Al2O3 Catalyst by Polyol Method and Its Application in Room Temperature Ozonation of Toluene in Air. *Catal. Lett.* **151**, 1418-1432 (2020).
66 Zhang, J., Xu, H., Ge, Q. & Li, W. Highly efficient Ru/MgO catalysts for NH_3 decomposition: Synthesis, characterization and promoter effect. *Catal. Commun.* **7**, 148-152 (2006).
67 Zheng, W., Zhang, J., Xu, H. & Li, W. NH_3 decomposition kinetics on supported Ru clusters: morphology and particle size effect. *Catal. Lett.* **119**, 311-318 (2007).
68 Yao, L. H., Li, Y. X., .Zhao, J., Ji, W. J. & Au, C. T. Core-shell structured nanoparticles (M@SiO_2, Al_2O_3, MgO; M=Fe, Co, Ni, Ru) and their application in CO_x-free H_2 production via NH_3 decomposition. *Catal. Today* **158**, 401-408 (2010).
69 Han, X. *et al.* Dual confinement of $LaCoO_x$ modified Co nanoparticles for superior and stable ammonia decomposition. *Appl. Catal. B: Environ.* **328** (2023).
70 Li, Y. *et al.* Core-shell structured microcapsular-like Ru@SiO_2 reactor for efficient generation of CO_x-free hydrogen through ammonia decomposition. *Chem. Commun.* **46**, 5298-5300 (2010).
71 Huo, L. *et al.* Spatial confinement and electron transfer moderating Mo N bond strength for superior ammonia decomposition catalysis. *Appl. Catal. B: Environ.* **294** (2021).
72 Huo, L. *et al.* Component synergy and armor protection induced superior catalytic activity and stability of ultrathin Co-Fe spinel nanosheets confined in mesoporous silica shells for ammonia decomposition reaction. *Appl. Catal. B: Environ.* **253**, 121-130 (2019).
73 Cha, J. *et al.* Highly monodisperse sub-nanometer and nanometer Ru particles confined in alkali-exchanged zeolite Y for ammonia decomposition. *Appl. Catal. B: Environ.* **283**, 119627 (2021).
74 Li, J. *et al.* Sub-nm ruthenium cluster as an efficient and robust catalyst for decomposition and synthesis of ammonia: Break the "size shackles". *Nano Res.* **11**, 4774-4785 (2018).
75 Akarçay, Ö., Kurtoğlu, S. F. & Uzun, A. Ammonia decomposition on a highly-dispersed carbon-embedded iron catalyst derived from Fe-BTC: Stable and high performance at relatively low temperatures. *Int. J. Hydrog. Energy* **45**, 28664-28681 (2020).
76 Gu, Y. *et al.* One-pot synthesis of supported Ni@Al2O3 catalysts with uniform small-sized Ni for hydrogen generation via ammonia decomposition. *Int. J. Hydrog. Energy* **46**, 4045-4054 (2021).
77 Tan, H. *et al.* Synthesis of Ru nanoparticles confined in magnesium oxide-modified mesoporous alumina and their enhanced catalytic performance during ammonia decomposition. *Catal. Commun.* **26**, 248-252 (2012).
78 Li, L., Zhu, Z. H., Yan, Z. F., Lu, G. Q. & Rintoul, L. Catalytic ammonia decomposition over Ru/carbon catalysts: The importance of the structure of carbon support. *Appl. Catal. A: Gen.* **320**, 166-172 (2007).
79 Lorenzut, B. *et al.* Embedded Ru@ZrO_2 Catalysts for H_2 Production by Ammonia Decomposition. *ChemCatChem* **2**, 1096-1106 (2010).
80 Nagaoka, K. *et al.* Influence of basic dopants on the activity of Ru/Pr_6O_{11} for hydrogen production by ammonia decomposition. *Int. J. Hydrog. Energy* **39**, 20731-20735 (2014).
81 Chen, J. *et al.* Effects of nitrogen doping on the structure of carbon nanotubes (CNTs) and activity of Ru/CNTs in ammonia decomposition. *Chem. Eng. J.* **156**, 404-410 (2010).
82 Hayashi, F. *et al.* Ammonia decomposition by ruthenium nanoparticles loaded on inorganic electride C12A7:e^-. *Chem. Sci.* **4**, 3124-3130 (2013).
83 Su, Q. *et al.* Layered double hydroxide derived Mg_2Al-LDO supported and K-modified Ru catalyst for hydrogen production via ammonia decomposition. *Catal. Lett.* **148**, 894-903 (2018).

84 Huang, C. *et al.* Ru/La_2O_3 catalyst for ammonia decomposition to hydrogen. *Appl. Surf. Sci.* **476**, 928-936 (2019).
85 Wang, Z., Cai, Z. & Wei, Z. Highly active ruthenium catalyst supported on barium hexaaluminate for ammonia decomposition to CO_x-Free hydrogen. *ACS Sustain. Chem. Eng.* **7**, 8226-8235 (2019).
86 Hu, X.-C. *et al.* Ceria-supported ruthenium clusters transforming from isolated single atoms for hydrogen production via decomposition of ammonia. *Appl. Catal. B: Environ.* **268**, 118424 (2020).
87 Wang, F. *et al.* The dispersed SiO_2 microspheres supported Ru catalyst with enhanced activity for ammonia decomposition. *Int. J. Hydrog. Energy* **46**, 20815-20824 (2021).
88 Fang, H. *et al.* Dispersed surface Ru ensembles on MgO(111) for catalytic ammonia decomposition. *Nat Commun* **14**, 647 (2023).
89 Li, Y., Liu, S., Yao, L., Ji, W. & Au, C.-T. Core-shell structured iron nanoparticles for the generation of CO-free hydrogen via ammonia decomposition. *Catal. Commun.* **11**, 368-372 (2010).
90 Feyen, M. *et al.* High-temperature stable, iron-based core-shell catalysts for ammonia decomposition. *Chemistry* **17**, 598-605 (2011).
91 Lorenzut, B., Montini, T., Bevilacqua, M. & Fornasiero, P. FeMo-based catalysts for H_2 production by NH_3 decomposition. *Appl. Catal. B: Environ.* **125**, 409-417 (2012).
92 Xun, Y. *et al.* Fe- and Co-doped lanthanum oxides catalysts for ammonia decomposition: Structure and catalytic performances. *J. Rare Earths* **35**, 15-23 (2017).
93 Hu, Z.-P., Chen, L., Chen, C. & Yuan, Z.-Y. Fe/ZSM-5 catalysts for ammonia decomposition to CO_x-free hydrogen: Effect of SiO_2/Al_2O_3 ratio. *Mol. Catal.* **455**, 14-22 (2018).
94 Zhang, Z.-S. *et al.* Promoted porous Co_3O_4-Al_2O_3 catalysts for ammonia decomposition. *Sci. China Chem.* **61**, 1389-1398 (2018).
95 Gu, Y.-Q. *et al. In situ* X-ray diffraction study of Co–Al nanocomposites as catalysts for ammonia decomposition. *J. Phys. Chem. C* **119**, 17102-17110 (2015).
96 Gu, Y.-Q. *et al.* Transition metal nanoparticles supported La-promoted MgO as catalysts for hydrogen production via catalytic decomposition of ammonia. *J. Energy Chem.* **38**, 41-49 (2019).
97 Yao, L. *et al.* Core-shell structured nickel and ruthenium nanoparticles: Very active and stable catalysts for the generation of CO_x-free hydrogen via ammonia decomposition. *Catal. Today* **164**, 112-118 (2011).
98 Yan, H. *et al.* Promoted multimetal oxide catalysts for the generation of hydrogen via ammonia decomposition. *J. Phys. Chem. C* **120**, 7685-7696 (2016).
99 Su, Q. *et al.* Layered double hydroxides derived $Ni_x(Mg_yAl_zO_n)$ catalysts: Enhanced ammonia decomposition by hydrogen spillover effect. *Appl. Catal. B: Environ.* **201**, 451-460 (2017).
100 Qiu, Y., Fu, E., Gong, F. & Xiao, R. Catalyst support effect on ammonia decomposition over Ni/$MgAl_2O_4$ towards hydrogen production. *Int. J. Hydrog. Energy* **47**, 5044-5052 (2022).
101 Tabassum, H. *et al.* Hydrogen generation via ammonia decomposition on highly efficient and stable Ru-free catalysts: approaching complete conversion at 450 °C. *Energy Environ. Sci.* **15**, 4190-4200 (2022).
102 Duan, X., Qian, G., Zhou, X., Chen, D. & Yuan, W. MCM-41 supported Co Mo bimetallic catalysts for enhanced hydrogen production by ammonia decomposition. *Chem. Eng. J.* **207-208**, 103-108 (2012).
103 ValeriaTagliazucca, Schlichte, K., Schüth, F. & Weidenthaler, C. Molybdenum-based catalysts for the decomposition of ammonia: In situ X-ray diffraction studies, microstructure, and catalytic properties. *J. Catal.* **305**, 277-289 (2013).
104 Jia, J. *et al.* Towards an efficient CoMo/γ-Al_2O_3 catalyst using metal amine metallate as an active phase precursor: Enhanced hydrogen production by ammonia decomposition. *Int. J. Hydrog. Energy* **39**, 12490-12498 (2014).
105 Tagliazucca, V., Leoni, M. & Weidenthaler, C. Crystal structure and microstructural changes of molybdenum nitrides traced during catalytic reaction by *in situ* X-ray diffraction studies. *Phys. Chem. Chem. Phys.* **16**, 6182-6188 (2014).
106 Sorcar, S. *et al.* Design of coke-free methane dry reforming catalysts by molecular tuning of nitrogen-rich combustion precursors. *Mater. Today Chem.* **24** (2022).
107 Duan, X. *et al.* Nickel–cobalt bimetallic catalysts prepared from hydrotalcite-like compounds for dry reforming of methane. *Int. J. Hydrog. Energy* **47**, 24358-24373 (2022).
108 Deng, J. *et al.* Cooperatively enhanced coking resistance via boron nitride coating over Ni-based catalysts for dry reforming of methane. *Appl. Catal. B: Environ.* **302** (2022).
109 Zhang, Y. *et al.* The tailored role of "defect" sites on γ-alumina: A key to yield an efficient methane dry reforming catalyst with superior nickel utilization. *Appl. Catal. B: Environ.* **315** (2022).
110 Kim, K. Y. *et al.* Layered double hydroxide-derived intermetallic $Ni_3GaC_{0.25}$ catalysts for dry reforming of methane. *ACS Catal.* **11**, 11091-11102 (2021).
111 Lu, Y. *et al.* Enhanced catalytic performance of Ni_x-V@HSS catalysts for the DRM reaction: The study of interfacial effects on Ni-VO_x structure with a unique yolk-shell structure. *J. Catal.* **396**, 65-80 (2021).
112 Tran, T. Q., Pham Minh, D., Phan, T. S., Pham, Q. N. & Nguyen Xuan, H. Dry reforming of methane over

calcium-deficient hydroxyapatite supported cobalt and nickel catalysts. *Chem. Eng. Sci.* **228** (2020).
113 Leba, A. & Yıldırım, R. Determining most effective structural form of nickel-cobalt catalysts for dry reforming of methane. *Int. J. Hydrog. Energy* **45**, 4268-4283 (2020).
114 Dong, J. *et al.* Reaction-induced strong metal-support interactions between metals and inert boron nitride nanosheets. *J. Am. Chem. Soc.* **142**, 17167-17174 (2020).
115 Peng, H. *et al.* Catalysts in coronas: A surface spatial confinement strategy for high-performance catalysts in methane dry reforming. *ACS Catal.* **9**, 9072-9080 (2019).
116 Akri, M. *et al.* Atomically dispersed nickel as coke-resistant active sites for methane dry reforming. *Nat Commun* **10**, 5181 (2019).
117 Phan, T. S. *et al.* Hydroxyapatite supported bimetallic cobalt and nickel catalysts for syngas production from dry reforming of methane. *Appl. Catal. B: Environ.* **224**, 310-321 (2018).
118 Stroud, T. *et al.* Chemical CO_2 recycling via dry and bi reforming of methane using Ni-Sn/Al_2O_3 and Ni-Sn/CeO_2-Al_2O_3 catalysts. *Appl. Catal. B: Environ.* **224**, 125-135 (2018).
119 Zhou, H. *et al.* A single source method to generate Ru-Ni-MgO catalysts for methane dry reforming and the kinetic effect of Ru on carbon deposition and gasification. *Appl. Catal. B: Environ.* **233**, 143-159 (2018).
120 Bian, Z. & Kawi, S. Highly carbon-resistant Ni–Co/SiO_2 catalysts derived from phyllosilicates for dry reforming of methane. *J. CO_2 Util.* **18**, 345-352 (2017).
121 Vasiliades, M. A. *et al.* Dry reforming of methane over 5 wt% Ni/$Ce_{1-x}Pr_xO_{2-\delta}$ catalysts: Performance and characterisation of active and inactive carbon by transient isotopic techniques. *Appl. Catal. B: Environ.* **197**, 168-183 (2016).
122 Fan, X. *et al.* Tuning the composition of metastable $Co_xNi_yMg_{100-x-y}(OH)(OCH_3)$ nanoplates for optimizing robust methane dry reforming catalyst. *J. Catal.* **330**, 106-119 (2015).
123 Paksoy, A. I., Caglayan, B. S. & Aksoylu, A. E. A study on characterization and methane dry reforming performance of Co–Ce/ZrO_2 catalyst. *Appl. Catal. B: Environ.* **168-169**, 164-174 (2015).
124 Du, X. *et al.* Dry reforming of methane over ZrO_2-supported Co–Mo carbide catalyst. *Appl. Petrochem. Res.* **4**, 137-144 (2014).
125 Takanabe, K., Nagaoka, K., Nariai, K. & Aika, K. Influence of reduction temperature on the catalytic behavior of Co/TiO_2 catalysts for CH_4/CO_2 reforming and its relation with titania bulk crystal structure. *J. Catal.* **230**, 75-85 (2005).
126 Selvaraj, M., Venkatachalapathy, V., Mayandi, J., Karazhanov, S. & Pearce, J. M. Preparation of meta-stable phases of barium titanate by sol-hydrothermal method. *AIP Advances* **5** (2015).
