# Supplementary material for: Synthesis of core@shell catalysts guided by Tammann temperature
Source: Nat Commun. 2024 Jan 10;15:420. doi: 10.1038/s41467-024-44705-5 (PMC10782006; doi:10.1038/s41467-024-44705-5)
Supplement: Supplementary file 3 — Description of Additional Supplementary Files [file 41467_2024_44705_MOESM3_ESM.pdf]

Description of Additional Supplementary Files

File Name: Supplementary Movie 1
Description: in-situ STEM observations at the $BaCO_3$ diffusion stage when the temperature is above $T_{Tam}(BaCO_3)$

File Name: Supplementary Movie 2
Description: in-situ STEM observations at the Co NPs formation stage when the temperature is above the $T_{red}(Co_3O_4)$

File Name: Supplementary Movie 3
Description: in-situ STEM observations at the Co@$BaAl_2O_4$ formation stage after the solid-state reaction between $BaCO_3$ and Al_2O_3

File Name: Supplementary Data 1
Description: Possible products and corresponding reaction enthalpy change (ΔH) of the solid-state reactions between $BaCO_3$ + Al_2O_3 + Co_3O_4 + H_2

File Name: Supplementary Data 2
Description: Possible products and corresponding reaction enthalpy change (ΔH) of the solid-state reactions between $BaCO_3$ + Al_2O_3 + NiO + H_2

File Name: Supplementary Data 3
Description: Possible products and corresponding reaction enthalpy change (ΔH) of the solid-state reactions between $MgCO_3$ + Al_2O_3 + CuO + H_2

File Name: Supplementary Data 4
Description: Possible products and corresponding reaction enthalpy change (ΔH) of the solid-state reactions between $BaCO_3$ + TiO_2 + Co_3O_4 + H_2
